# Enantioselective synthesis and racemization dynamics of trithia[5]helicenes derived from the dithieno[2,3-*b*:3′,2′-*d*]-thiophene unit

**DOI:** 10.1039/d5sc06132f

**Published:** 2025-09-23

**Authors:** Wei Fu, Martin Simon, Christopher Golz, Manuel Alcarazo

**Affiliations:** a Institut für Organische und Biomolekulare Chemie, Georg-August-Universität Göttingen Tammannstr 2 37077 Göttingen Germany manuel.alcarazo@chemie.uni-goettingen.de

## Abstract

A series of configurationally stable trithia[5]helicenes having the dithieno[2,3-*b*:3′,2′-*d*)-thiophene unit embedded in their skeletons has been prepared *via* a Au-catalyzed double alkyne hydroarylation reaction. The transformation proceeds with excellent yield, exquisite regioselectivity and high enantioselectivity. The absolute configuration of the newly prepared helicenes has been established by X-ray crystallography, and their inversion barriers were experimentally determined. Preliminary studies on the post-synthetic functionalization of the new structures prepared indicate that only the two peripheral thiophene units are oxidized to the corresponding sulfones, while their bromination occurs selectively at positions 12 and 15 of the bis-benzannulated bis(benzo[4,5]thieno)[2,3-*b*:3′,2′-*d*]thiophene core.

## Abstract 2

Two successive Au-catalyzed alkyne hydroarylation events allow the assembly of trithia[5]helicenes with high enantioselectivity. The racemization barrier of the structures prepared was experimentally determined (Δ*G*^≠^ = 35.6 kcal mol^−1^ at 175 °C).

## Graphical abstract

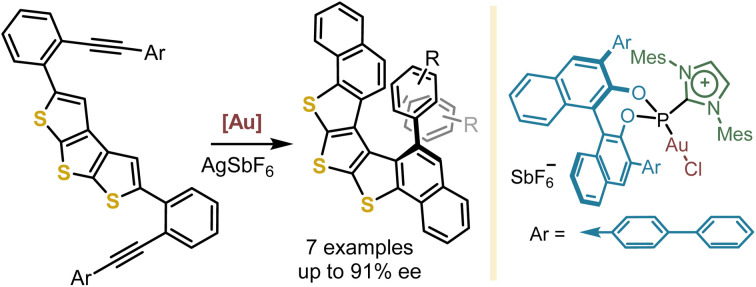

## Introduction

Due to their broad range of applications in areas of technological interests such as the development of organic semiconductors,^1^ dyes,^2^ liquid crystals,^3^ and photoactive compounds,^4^ thiahelicenes, have attracted a lot of attention.^5^ Their exceptional properties derive from the formal substitution of one or more benzene rings by thiophenes in the original polyacene skeleton. This modification confers: (i) lower oxidation potentials than carbohelicenes of analogue structure;^6^ (ii) predictable derivatization of their skeleton, either by site-selective introduction of substituents on their external rim,^7^ or by sulfur/(hetero)atom exchange;^8^ and (iii) new directional C–S, S–S and S–π interactions, which in addition to the omnipresent π-stacking, are essential for the charge carrier transport.^9^ These interactions have their origin in the polarizability of the S-atom in thiophene-based structures, and readily exert control on the internal structure of the crystals and films formed by thiahelicenes.^10^ On the other hand, lower racemization barriers are systematically observed for thiahelicenes when compared with carbohelicenes of the same order.^7*a*,11^ This is a consequence of the diminished wedge angle (*φ*) of thiophene (45°) when compared with that of benzene (60°), which increases the helix diameter, and reduces the overlap between the helicene's arms. It is probably for that reason that only mono- and dithia[*n*]helicenes (*n* = 5, 6) have been synthesized through catalytic enantioselective routes to date.^7*a*,*b*^

**Fig. 1:**
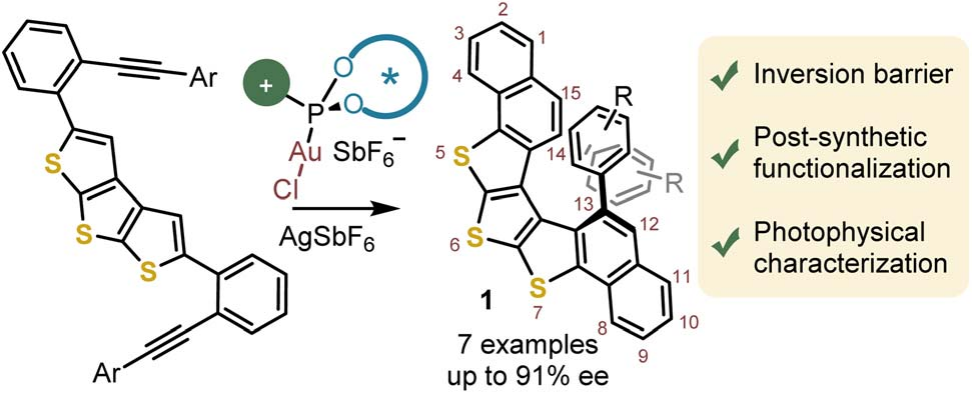
Enantioselective synthesis of helicenes derived from bis-benzannulated bis(benzo[4,5]thieno)[2,3-*b*:3′,2′-*d*]thiophene.

Being aware of that limitation, but also of the specific role that oligothiophenes play in the design of optoelectronic devices,^12^ we decided to evaluate whether the enantioselective Au-catalyzed alkyne hydroarylation method developed by our group for the synthesis of [4]-, [5]-, and [6]helicenes could be extended to helical structures containing three consecutively annulated thiophene units embedded in their skeletons.^13^ Specifically, we envisaged that a double intramolecular hydroarylation reaction on a dithieno[2,3-*b*:3′,2′-*d*)-thiophene core (DTT) has the potential to deliver helically chiral bis(benzo[4,5]thieno)[2,3-*b*:3′,2′-*d*]thiophene architectures provided that appropriate substituents are installed at the entrance of the fjord region.^9*b*,14^ This article reports the practical realization of this objective through the enantioselective synthesis of trithia[5]helicenes of general formula 1 (Fig. 1). Preliminary results on the racemization dynamics and post-synthetic functionalization of these structures are also described.

**Scheme 1:**
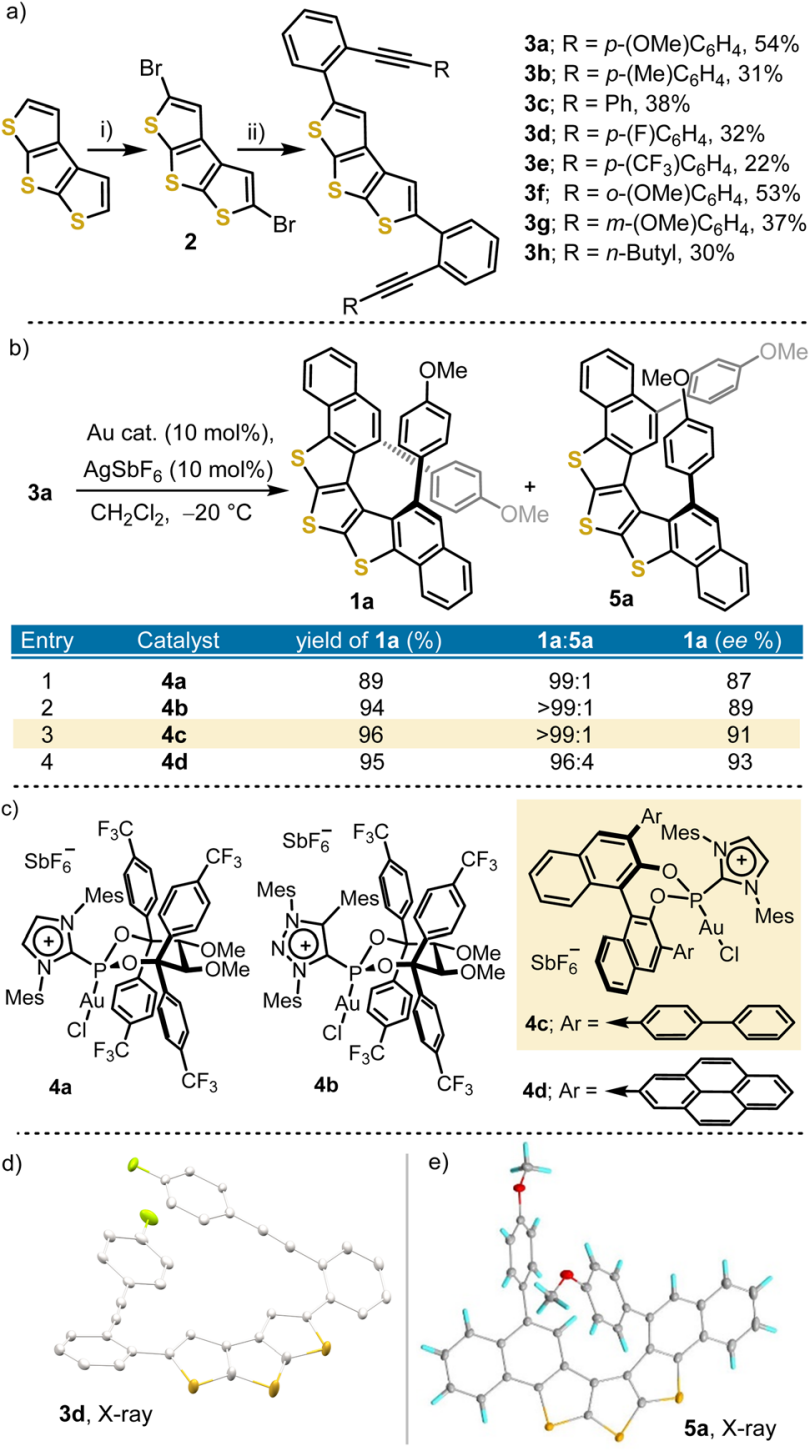
(a) Synthesis of diyne precursors 3a–h. Reagents and conditions: (i) NBS (2.0 equiv.), HCCl_3_/AcOH, r. t., 68%; (b) boronic acid (3.0 equiv.), Pd_2_dba_3_ (8.0 mol%), SPhos (16.0 mol%), and Cs_2_CO_3_ (4.0 equiv.), THF/H_2_O (10 : 1, 0.02 M), 80 °C, 24 h; (b and c) catalyst screening for substrate 3a; (d) X-ray structure of 3d; (e) X-ray structure of 5a. Anisotropic displacements are shown at the 50% probability level. Solvent molecules are removed for clarity.

Our study started with the preparation of model substrates 3a–h, which were synthesized from DTT *via* initial treatment with bromine to obtain the 2,5-dibromo derivative 2,^15^ and subsequent Suzuki coupling between that tricyclic substrate and the corresponding alkynyl-substituted boronic acids (Scheme 1a). Once obtained, compound 3a was taken as a model and submitted to the effect of four Au-precatalysts 4a–d, which based on our experience were considered the most promising ones for the key enantioselective cyclization. Au complexes 4a–b share a (2*R*,3*R*)-2,3-dimethoxy-1,1,4,4-tetrakis(4-(trifluoromethyl)phenyl)butane-1,4-diol backbone, but they differ in the heterocycle that carries the positive charge; an imidazolium one in 4a, and a 1,2,3-triazolium unit in 4b. Both pre-catalysts have demonstrated excellent enantioinduction for the assembly of carbo[4]-, and carbo[6]helicenes.^13*a*,*c*^ Additionally, BINOL-derived complexes 4c–d were included in the screening due to their suitability to promote asymmetric hydroarylation reactions that lead to carbo[5]-, oxa[5]-, and thia[5]helicenes, respectively.^13*b*,16^ All assays were carried out at −20 °C employing dichloromethane as a solvent (Scheme 1b).

Under these conditions, both precatalysts based on the BINOL scaffold were slightly superior in terms of enantioselectivity (Scheme 1b; entries 3 and 4); however, 4d also promoted the minor formation of a side species 5a, which was later isolated and identified as the 1,2-aryl migration product by X-ray diffraction analysis (Scheme 1e). The formation of 5a resembles the previously proposed halide walk phenomenon that has been rationalized by assuming the formation of a Au vinylidene intermediate.^17^ Hence, precatalyst 4c, bearing *p*-biphenyl groups attached to the 3,3′-positions of the BINOL subunit, and able to furnish the desired cyclisation towards 1a with complete conversion, excellent regioselectivity (1a : 5a; >99 : 1), and high enantioselectivity (91%) was used to further investigate the scope of the transformation.

**Fig. 2:**
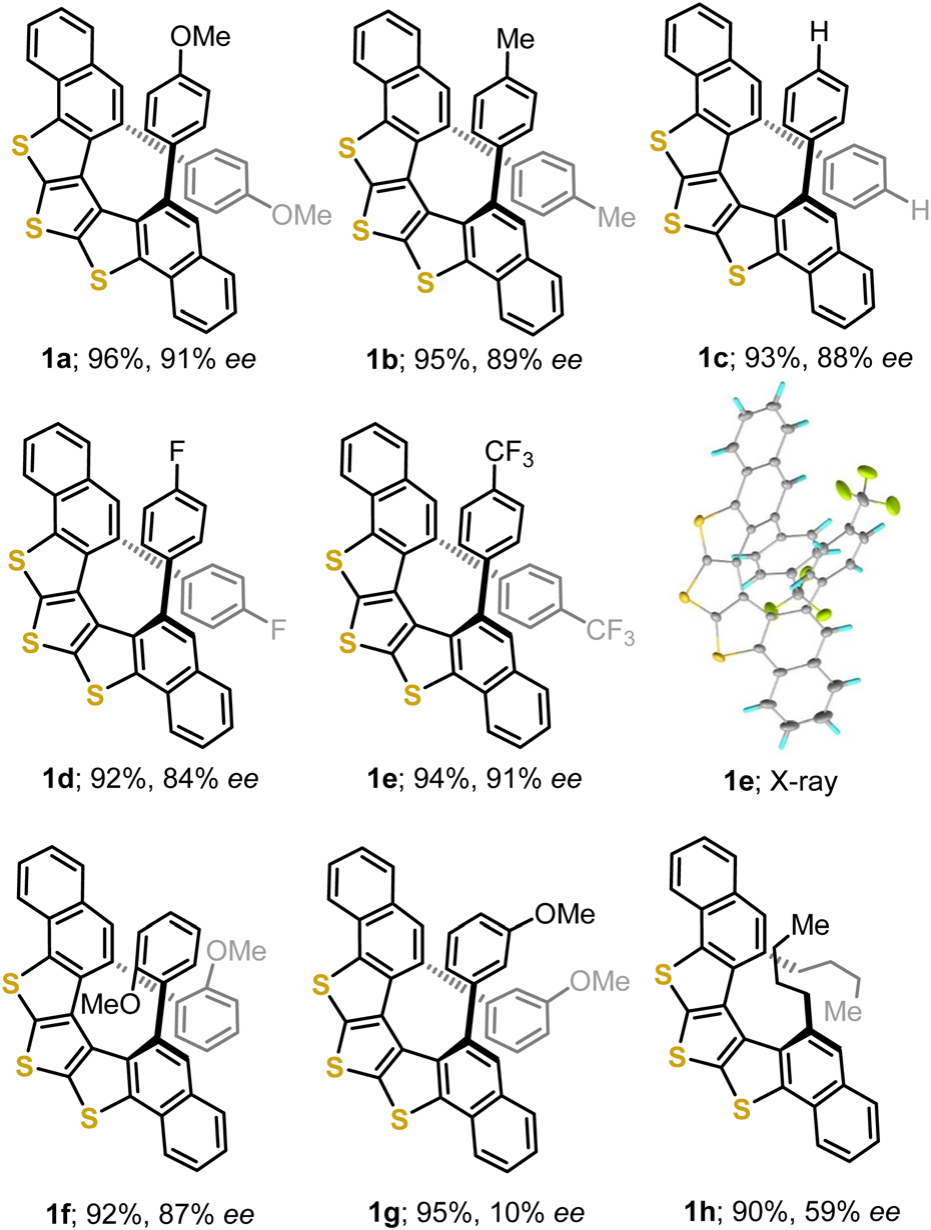
Scope of the asymmetric Au-catalysed cyclisation. For the X-ray structure of 1e, anisotropic displacements are shown at the 50% probability level.

The cyclization of diynes 3a–e led to helicenes 1a–e, in excellent yields (92–96%) and high enantioselectivities (84–91% ee) when the substituents are located at the *para*-position of the terminal aromatic rings (Fig. 2). Substituents located at the *o*-position of the terminal phenyl rings are also tolerated 1f (92%, 87% ee), but a *m*-substitution drastically erodes the enantioselectivity of the transformation 1g (95%, 10% ee). Similarly, diyne 3h, bearing *n*-butyl groups at both alkyne termini, delivers trithia[5]helicene 1h in high yield but only moderate enantioselectivity (90%, 60% ee). Substituents able to coordinate Au, such as pyridines, inhibit the cyclisation. These limitations of catalyst 4c had been already detected during the synthesis of carbo[5]- oxa[5]-, and thia[5]helicenes,^13*b*,16^ and will probably need a new catalyst design to be addressed.

**Fig. 3:**
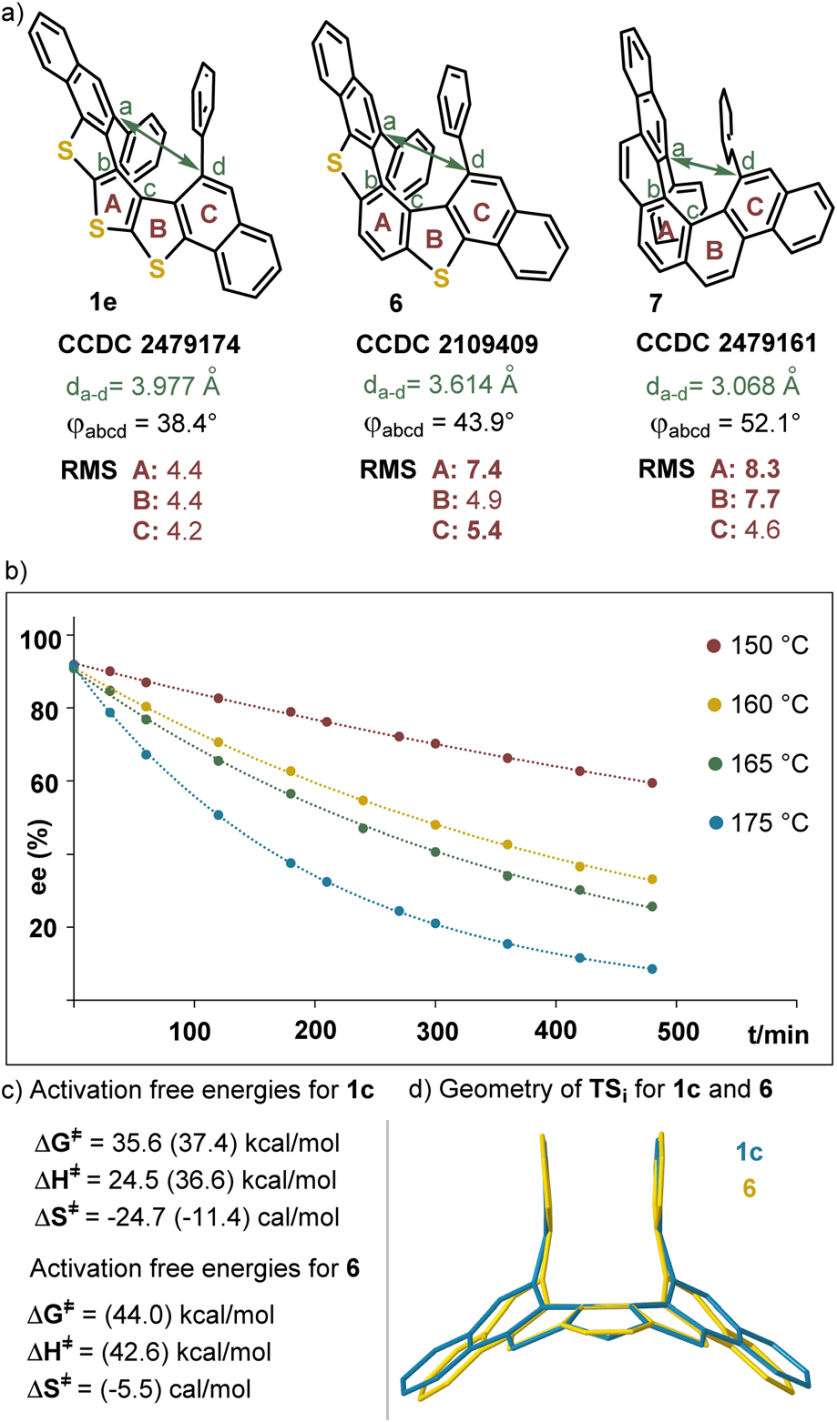
(a) Geometric parameters from the structures of 1e, 6 and 7. RMS are in μm; (b) time-course evolution of the ee of 1a at different temperatures; (c) experimental activation-free energies of racemization for 1a at 175 °C, and calculated values (in parentheses) at the B3LYP-D3/def2-TZVP level of theory in DCM using the conductor-like polarizable continuum model method;^18^ (d) calculated transition state structures for inversion of 1c (blue) and 6 (yellow).

The connectivity of the DTT-containing [5]helicenes was unambiguously confirmed through the X-ray crystallographic analysis of single crystals of 1e and *rac*-1f (Fig. 2 and SI); from the analysis of 1e the absolute configuration of that compound was determined to be *P*. Both the Flack (0.008(9)) and the Hooft parameters (0.008(9)) support this assignment. A comparison of the heteroaromatic skeleton of 1e, dithia[5]helicene 6, and [5]helicene 7, all diaryl substituted at the entrance of their fjord region is quite informative as well (Fig. 3). As a consequence of the smaller wedge angle (*φ*) of thiophene, the progressive incorporation of several of these heterocyclic units makes the helix wider and reduces both, the overlap between the arms, and the helical pitch. This translates into an increased distance between the atoms located at the entrance of the fjord (*d*_a–d_), and a reduction of the distortion from ideal planarity of the (hetero)arene core of the helix, as measured through the root mean square deviations (RMS) of each arene moiety.

The racemization dynamics of the newly prepared trithia[5]helicene skeleton has been evaluated using 1a as a model substrate. Initially, we determined the racemization rate constants at different temperatures (150–175 °C), and subsequently the activation-free energy (Δ*G*^≠^) for the helix inversion was calculated through an Eyring plot (Fig. 3b, c and SI). As expected from the geometric parameters already discussed, the racemization of 1a (Δ*G*^≠^ = 35.6 kcal mol^−1^ at 175 °C) is easier than that of 6, a compound that does not show any sign of racemization after being heated at 180 °C for 48 hours.^7*b*^ The situation is even more evident when the free energies of racemization calculated at the B3LYP-D3/def2-TZVP level of theory are compared (1c, Δ*G*^≠^ = 37.4 kcal mol^−1^; 6, 44.0 kcal mol^−1^) (Fig. 3c),^18,19^ and the structures of the corresponding transition states TS_i_ are shown overlapped. Dithia[5]helicene 6 (yellow) has to twist more significantly than 1c (blue) in order to reach the transition state for enantiomer interconversion (Fig. 3d).

**Scheme 2:**
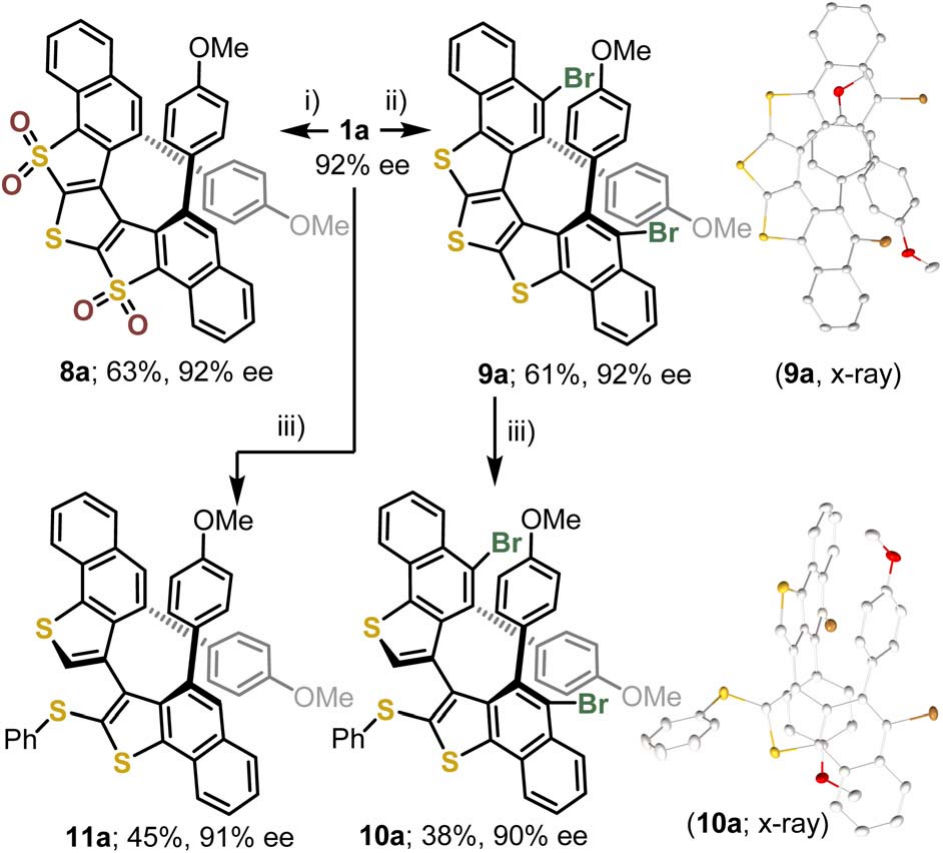
Post-synthetic modification of trithia[5]helicenes. Reagents and conditions: (i) *m*-CPBA (5.0 equiv.), CH_2_Cl_2_ (0.1 M), −10 °C, 12 h; (ii) NBS (3.0 equiv.), HCCl_3_ (0.02 M), 35 °C, 24 h; (iii) PhB(OH)_2_ (4.0 equiv.), Pd(dppf)Cl_2_ (10.0 mol%), KF (4.0 equiv.), tol : MeOH (1 : 1), 110 °C, μW, 12 h.

The possibility of functionalizing the trithia[5]helicenes prepared was evaluated using 1a as a model. Initially, the oxidation of the DTT unit was attempted with an excess of *m*-chloroperbenzoic acid (*m*-CPBA; 5.0 equiv.). This reaction cleanly delivers bis-sulfone 8a, in which the S-atoms at positions 5 and 7 have been oxidized, but not the central one (Scheme 2). A monosulfone intermediate could never be cleanly obtained; mixtures of mono- and bis-sulfone were observed even if only 2.0 equiv. of *m*-CPBA were used for the oxidation. On the other hand, the tri-sulfone was never detected even if the oxidation conditions were forced (*m*-CPBA; 10.0 equiv.). Subsequently, the bromination of 1a with *N*-bromosuccinimide was evaluated. Under the conditions applied (NBS, 3.0 equiv.), the electrophilic bromination occurred in a synthetically useful 61% yield to deliver 9a, in which the two bromo substituents are selectively incorporated at positions 12 and 15 of the bis-benzannulated bis(benzo[4,5]thieno)[2,3-*b*:3′,2′-*d*]thiophene core. Considering the simplicity of the protocol applied, this is a remarkable result; however, selective monobromination of 1a was not achieved. No erosion of the enantiopurity was detected in any of these post-synthetic functionalization steps.

Finally, we attempted a double Suzuki coupling on 9a employing phenylboronic acid as a nucleophilic partner, but instead of the expected incorporation of two phenyl groups at positions 12 and 15, product 10a was isolated. In 10a the two bromine substituents remain intact while the central thiophene unit has been opened. The formation of 10a can be rationalized through the initial oxidative addition of a C–S bond from the central thiophene to Pd^0^, protodemetallation of the formed Pd–C bond, B to Pd transmetallation of the phenyl group, and final reductive coupling to form 10a and recover the Pd^0^-catalyst (Scheme 2). The transformation of 9a into 10a occurred with complete helical-to-axial chirality transfer. When 1a is submitted to the same reaction conditions, identical reactivity is observed and 11a is obtained (Scheme 2).

**Fig. 4:**
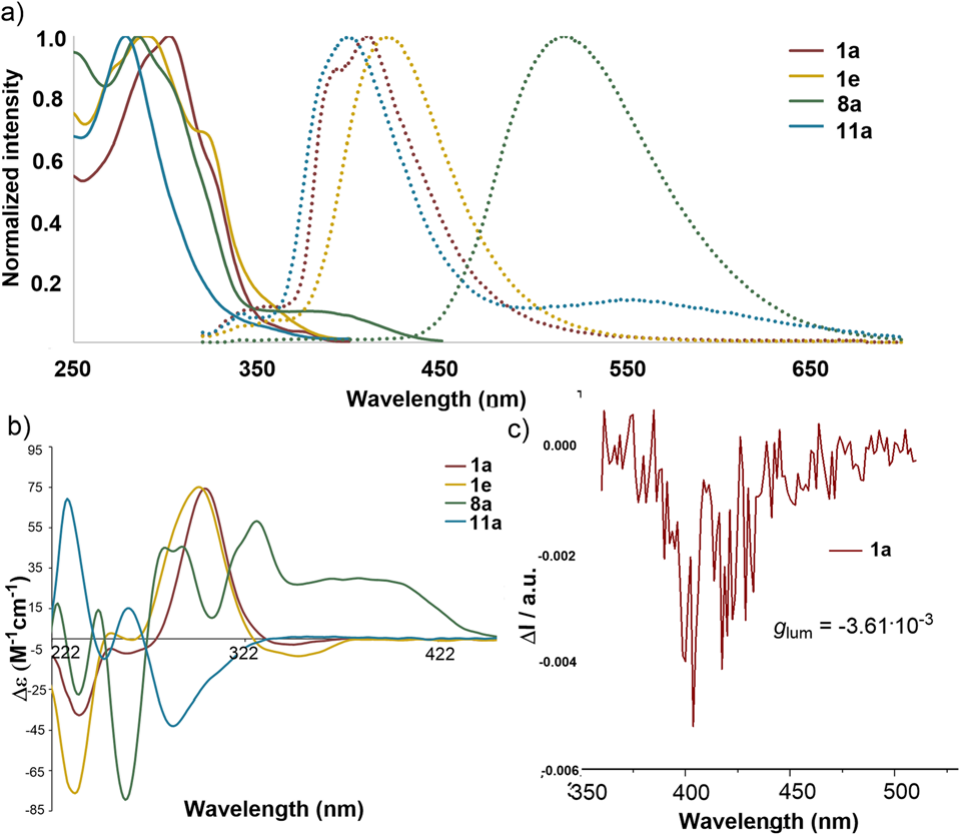
Photophysical and chiroptical properties of the trithia[5]helicene derivatives. (a) UV/vis (continuous line) and fluorescence spectra (dotted line) of selected compounds in CH_2_Cl_2_ (1 × 10^−5^ M); (b) CD spectra in CH_2_Cl_2_ (1 × 10^−5^ M); (c) CPL spectra of 1a.

Next, the photophysical properties of selected trithia[5]helicenes 1a, 1e, 8a, and 11a were examined. The UV-vis spectra of these compounds exhibited similar absorption peaks with maxima between 280 and 305 nm (11a, 280 nm; 8a, 286 nm; 1e, 288 nm; 1a, 304 nm). Their fluorescence emission wavelengths range from 350 to 650 nm, with disulfone-based helicene 8a showing a notable redshift and having its emission maxima at 540 nm (Fig. 4a). The fluorescence quantum yields are low in all cases (1a, *Φ*_F_ = 0.6%; 1e, *Φ*_F_ = 0.6%; 8a, *Φ*_F_ = 1.4%, and 10a, *Φ*_F_ = 0.8%). Previous reports on structurally related [5]helicenes describe similarly low fluorescence quantum yields for thiahelicenes; this is attributed to a rapid intersystem crossing process.^20^

Inspection of the circular dichroism (CD)-spectra for 1a–h (Fig. 4b and the SI) manifests clear similarities in shape and sign and suggests that all trithia[5]helicenes synthesized share the same absolute configuration. Finally, the circular polarized luminescence spectrum (CPL) of 1a was characterized by a luminescence dissymmetry factor (*g*_lum_) of 3.61 × 10^−3^ (Fig. 4c).

## Conclusions

A high yielding method for the enantioselective synthesis of trithia[5]helicenes through a Au-catalysed double hydroarylation reaction has been developed. The use of BINOL-derived α-cationic phosphonite ancillary ligands proved to be essential to provide high levels of enantioinduction. The absolute configuration of the newly prepared helices was determined to be *P* through X-ray crystallography, and the racemization barrier of the structures prepared was experimentally determined (Δ*G*^≠^ = 35.6 kcal mol^−1^ at 175 °C for 1a). Moreover, preliminary studies on the post-synthetic functionalization of the initially obtained trithia[5]helicenes show that their dibromination with NBS occurs regioselectively at positions 12 and 15. Oxidation of 1a to the corresponding disulfone 8a results in a substantial red-shift of the emission maxima by ≈130 nm, but all compounds studied show low fluorescence quantum yields (*Φ*_F_ = 0.6–1.4%).

## Author contributions

W. F. and M. A. conceived the project and designed the experiments. W. F. performed the experiments and analysed the results. M. S. carried out HPLC purifications and determined the enantiomeric excesses. C. G. performed the X-ray crystallographic analysis. All authors discussed the results, and M. A. wrote the manuscript.

## Conflicts of interest

There are no conflicts to declare.

## Supplementary Material

SC-OLF-D5SC06132F-s001

SC-OLF-D5SC06132F-s002

## Data Availability

All data associated with this article are available in the SI. CCDC 2479173–2479180 contain the supplementary crystallographic data for this paper.^21*a*–*h*^ Supplementary information is available: experimental procedures, NMR spectra and HPLC chromatograms. See DOI: https://doi.org/10.1039/d5sc06132f.
