# Supplementary material for: Enantioselective synthesis and racemization dynamics of trithia[5]helicenes derived from the dithieno[2,3-b:3′,2′-d]-thiophene unit
Source: Chem Sci. 2025 Sep 23;16(41):19172–7. doi: 10.1039/d5sc06132f (PMC12455664; doi:10.1039/d5sc06132f)
Supplement: SC-016-D5SC06132F-s001 [file SC-016-D5SC06132F-s001.pdf]

Supplementary Information (SI) for Chemical Science.
This journal is © The Royal Society of Chemistry 2025

Enantioselective synthesis and racemization dynamics of trithia[5]helicenes derived from the dithieno[2,3-*b*:3,2-*d*]thiophene unit

Wei Fu,[a] *Martin Simon,*[a] *Christopher Golz,*[a] *and Manuel Alcarazo*[a,*]

[a] W. Fu, M. Simon, C. Golz, M. Alcarazo
Institut für Organische und Biomolekulare Chemie
Georg-August-Universität Göttingen
Tammannstr 2, 37077 Göttingen, Germany
E-mail: manuel.alcarazo@chemie.uni-goettingen.de

Table of Contents

MATERIALS AND METHODS 4
SYNTHESIS AND CHARACTERIZATION 6
General procedure for the synthesis of alkyne precursors **3ah** 6
Synthesis and characterization of helicenes 11
General procedure for the synthesis of thiahelicenes 11
Synthesis of **8a**: 17
Synthesis of **9a** 18
Procedure for the synthesis of **10a** and **11a** 18
SPECTROSCOPIC DATA 21
Compound **3a** 21
Compound **3b** 22
Compound **3c** 23
Compound **3d** 24
Compound **3e** 26
Compound **3f** 28
Compound **3g** 29
Compound **3h** 30
Compound **S2d** 31
Compound **S2h** 33
Compound **1a** 34
Compound **1b** 35
Compound **1c** 36
Compound **1d** 37
Compound **1e** 39
Compound **1f** 41
Compound **1g** 42
Compound **1h** 43
Compound **5a** 44
Compound **8a** 45
Compound **9a** 46
Compound **11a** 47
Compound **10a** 48
HPLC-Chromatograms 49
Chiroptical Properties 61
Circular Dichroism 61
X-RAY CRYSTALLOGRAPHIC ANALYSIS 66
General 66

Refinement details 67
Computational Studies 75
Racemization Experiments 81
REFERENCES 83

MATERIALS AND METHODS

Unless stated otherwise, all reactions were carried out using pre-dried glassware under an inert atmosphere (nitrogen or argon) using standard Schlenk techniques, or in a MBraun UNIlab plus glovebox. After quenching, the reaction mixtures were concentrated under reduced pressure by rotary evaporation at 25–55 °C. Purified compounds were further dried under high vacuum. Yields refer to purified and spectroscopically pure compounds.

Solvents: Dry and degassed solvents (dichloromethane and toluene,) were obtained from a MBraun Solvent Purification System (MB-SPS-800) or by distillation over the appropriate drying agent and stored under a protective gas atmosphere.

Chromatography: Thin layer chromatography (TLC) was performed using polygram SIL G/UV254 TLC plates from Macherey Nagel and visualized by UV irradiation and/or phosphomolybdic acid or $KMnO_4$ dip. Flash column chromatography was performed using Macherey Nagel 60 (40-63 µm) silica gel.

Starting materials: Commercially available reagents were purchased from Acros Organics, ABCR, Alfa Aesar Sigma Aldrich, Thermo Fisher Scientific, Tokyo Chemical Industry, and used as received. Silver hexafluoroantimonate ($AgSbF_6$) was purchased from Sigma-Aldrich, transferred into a glovebox and finely ground using a pestle and mortar. The chiral TADDOL-based Au(I) complexes **4a**[1] and **4b**[2], the chiral BINOL-based Au(I) complexes **4c**-**d**[3], and the cationic non-chiral Au(I)-precatalyst **S3**[4] were prepared as previously reported.

NMR: Spectra were recorded on Bruker Avance Neo 600, Avance Neo 400, Avance III HD 400, Avance III 400 or Avance III HD 300 spectrometers. ^{1}H and ^{13}C chemical shifts (δ) are reported in ppm relative to TMS using the solvent signals as reference in $CDCl_3$ (^{1}H: 7.26 ppm, ^{13}C: 77.16 ppm) or CD_2Cl_2 (^{1}H: 5.32 ppm, ^{13}C: 53.84 ppm). Coupling constants (*J*) are given in Hertz (Hz). Data is reported as follows: s = singlet, d = doublet, t = triplet, q = quartet, m = multiplet, br = broad; coupling constants in Hz; integration.

HRMS: Spectra were recorded using Bruker Daltonik maXis Q-TOF (ESI), Bruker Daltonik micrOTOF (ESI), Thermo Scietific LTQ Orbitrap XL (ESI), Thermo Scietific Exactive GC-Orbitrap-MS (EI) or Jeol AccuTOF (EI) instruments. Dimensionless mass-to-charge ratios (*m*/*z*) are given.

IR: Infrared spectra were recorded on a FT/IR-4600 spectrometer and reported in wavenumbers (cm^{-1}).

Melting point: Melting points were measured with a Büchi M-560 apparatus with a heating rate of 5 °C/min.

Chiral HPLC: chiral HPLC measurements were performed using a Shimadzu Prominence-i LC2030C 3D Plus with integrated downstream UV/Vis PDA detector. System control and chromatogram analysis were carried out with LabSolutions software version 5.92. Enantioselective separations were conducted on a Chiralpak® IA-3 (150 mm, i.d. 4.6 mm, particle size 3 µm) or a Chiralpak® IC-3 (150 mm, i.d. 4.6 mm, particle size 3 µm) column, which were bought from Daicel Chiral Technologies. The solvents used (*n*-hexane, *iso*-propanol, ethyl acetate) were purchased from Fisher Scientific or Sigma-Aldrich in HPLC-grade quality. Specific conditions, such as eluent mixtures, flow rates and temperatures are provided for each compound individually.

Specific rotations: were collected using Jasco P-2000 polarimeters at the stated temperature under a Na/Hg lamp, λ = 589 nm (c in g/mL).

Chiroptical Properties: **Circular dichroism spectra** were measured on a Jasco J-1500 spectrometer using a 10 mm quartz sample cell using sample concentrations of 10 µM in CH_2Cl_2. **Circularly polarized luminescence** spectra were performed in an Olis DSM172 spectrophotometer using a 1.0 cm path-length quartz cell. A fixed wavelength LED (270 nm) was used as the excitation source. In all the cases a fixed slit-width of 1 mm and 0.5 s of integration time were selected and shown spectra are the result of accumulating 30 scans.

UV/Vis spectra were measured using a Jasco J-1500 spectrometer using a 10 mm quartz sample cell. Fluorescence spectra were measured using a Jasco FP-8500 spectrofluorometer using a 10 × 2 mm quartz sample cell. The standard was a solution of 9,10-diphenylanthracene in cyclohexane. Sample concentrations in CH_2Cl_2 were adapted to have an absorbance lower than 0.1 at the excitation wavelength (λ_{ex}). **Fluorescence lifetime** measurements were conducted using an Edinburgh Instruments FS5 Spectrofluorometer using a SC-05 cuvette holder. For Irradiation an Edinburgh Instruments EPLED-310 (λ= 310 ± 10 nm, P= 40 µW) was used using a pulse period of 50 or 100 nm. Emission was detected by a PMT-900 detector. Operation of the spectrofluorometer and analysis was performed using the Fluoracle software. The detection wavelength of the instrument was set to the maximum of the emission of the sample with a shutter length of 20-25 nm and using 1024 channels and a time range of 50-100 ns. The measurement was conducted for 30 minutes or until a photon count of 10000 was achieved. Measurements were conducted using quartz cuvettes. For the samples a 4x10 mm cuvette was used with the light source pointing along the long axis of the cuvette. For the IRF, a scattering non-emissive sample (Prepared from LUDOX® AM colloidal silica 30 wt.% suspension in water purchased from Sigma Aldrich) in a 10 × 10 mm cuvette was used.

SYNTHESIS AND CHARACTERIZATION

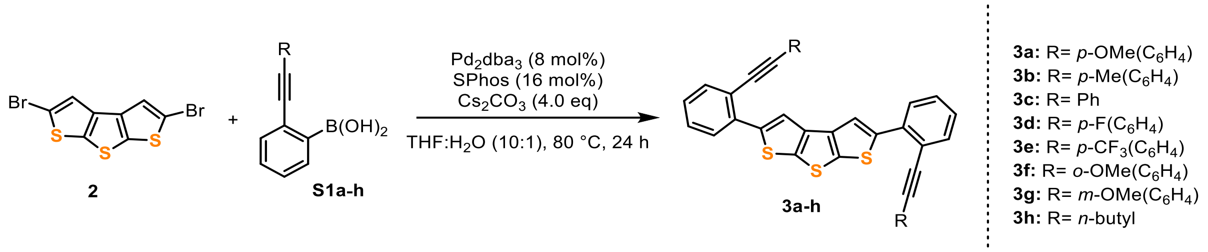

*Scheme S 1: Synthesis of alkynes **3a-h**.*

General procedure for the synthesis of alkyne precursors **3ah**

Under Argon atmosphere, **2** (0.25 mmol), boronic acid **S1** (3.0 equiv.), Pd_2dba_3 (8 mol%), SPhos (16 mol%) and Cs_2CO_3 (4.0 equiv.) were added to a Schlenk flask equipped with a magnetic stir bar. Then, THF and H_2O (degassed with Argon for 15 min, 10:1, 0.02 M) were added and the flask was sealed with a septum-cap and stirred at 80 °C for 24 h. After this, the reaction mixture was filtered through a silica plug eluting with DCM and the solvent was removed under reduced pressure. The residue was purified by chromatography on silica gel eluting with the indicated solvent mixture to afford the desired alkyne product.[3]

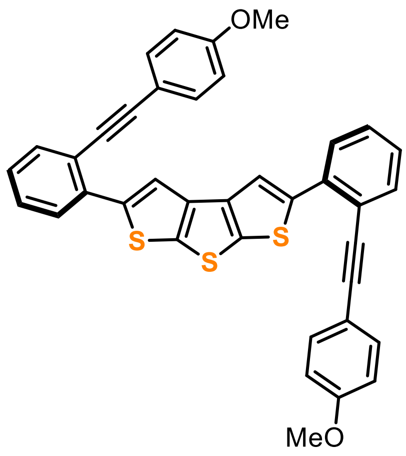

2,5-bis(2-((4-methoxyphenyl)ethynyl)phenyl)dithieno[2,3-*b*:3',2'-*d*]thiophene **3a**: Prepared following the general procedure from **2** (86.3 mg, 0.25 mmol, 1.0 equiv.) and boronic acid **S1a** (189.1 mg, 0.75 mmol, 3.0 equiv.). The column chromatography was eluted with Hexane/EtOAc (10/1 (v/v)); **3a** was obtained as a yellow solid (82 mg, 54%). **^{1}H-NMR** (300 MHz, CD_2Cl_2): δ = 7.99 (s, 2H), 7.67 (t, J = 5.7 Hz, 4H), 7.43 (d, J = 8.8 Hz, 4H), 7.41 – 7.29 (m, 4H), 6.85 (d, J = 8.8 Hz, 4H), 3.78 (s, 6H). **^{13}C{^{1}H}-NMR** (101 MHz, CD_2Cl_2) δ = 160.4 (2Cq), 145.3 (2Cq), 139.5 (2Cq), 139.0 (2Cq), 136.0 (2Cq), 133.9 (2CH), 133.2 (4CH), 129.1 (2CH), 128.9 (2CH), 127.9 (2CH), 121.2 (2Cq), 118.7 (2CH), 115.5 (2Cq), 114.5 (4CH), 94.9 (2C≡C), 88.4 (2C≡C), 55.7 ($2OCH_3$).

IR (ATR): $\tilde{\nu}$ = 2931, 2834, 2211, 1605, 1591, 1567, 1509, 1474, 1455, 1414, 1328, 1303, 1287, 1244, 1174, 1146, 1105, 1063, 1031, 940, 912, 862, 824, 781, 751, 686, 657, 640, 593, 569, 531, 485, 461 cm^{-1}

HRMS-ESI (m/z) calculated for $C_{38}H_{24}NaO_2S_3^+$ $[M+Na]^+$: 631.0831; found, 631.0826.

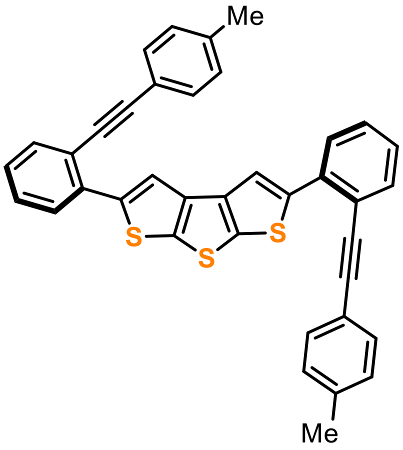

2,5-bis(2-(*p*-tolylethynyl)phenyl)dithieno[2,3-*b*:3',2'-*d*]thiophene **3b**: Prepared following the general procedure from **2** (86.3 mg, 0.25 mmol, 1.0 equiv.) and boronic acid **S1b** (177.1 mg, 0.75 mmol, 3.0 equiv.). The column chromatography was eluted with Hexane/DCM (5/1 (v/v)); **3b** was obtained as a yellow solid (45 mg, 31%).
^{1}H-NMR (300 MHz, $CDCl_3$): δ = 7.94 (s, 2H), 7.66 (ddd, J = 7.7, 5.1, 1.8 Hz, 4H), 7.42 – 7.36 (m, 4H), 7.32 (qd, J = 7.5, 7.0, 1.5 Hz, 4H), 7.12 (d, J = 7.9 Hz, 4H), 2.34 (s, 6H). **^{13}C{^{1}H}-NMR** (101 MHz, $CDCl_3$) δ = 145.0 (2Cq), 139.1 (2Cq), 138.81 (2Cq), 138.79 (2Cq), 136.0 (2Cq), 133.8 (2CH), 131.5 (4CH), 129.4 (4CH), 128.9 (2CH), 128.7 (2CH), 127.5 (2CH), 120.9 (2Cq), 120.3 (2Cq), 118.4 (2CH), 94.8 (2C≡C), 88.9 (2C≡C), 21.7 ($2CH_3$).

IR (ATR): $\tilde{\nu}$ = 3026, 2916, 2859, 2210, 1902, 1591, 1560, 1509, 1474, 1437, 1364, 1328, 1274, 1201, 1180, 1160, 1103, 1062, 1036, 1019, 944, 911, 869, 812, 751, 707, 693, 658, 647, 596, 570, 534, 516, 499, 491, 460, 416, 403 cm^{-1}

HRMS-EI (m/z) calculated for $C_{38}H_{24}S_3^+$ [M]$^+$: 576.1035; found, 576.1030.

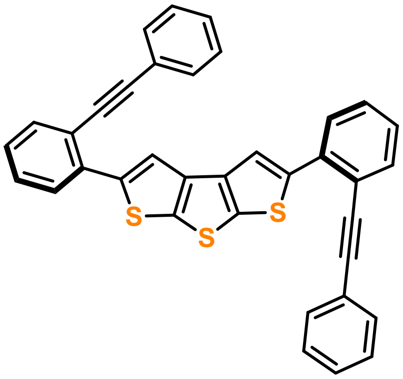

2,5-bis(2-(phenylethynyl)phenyl)dithieno[2,3-*b*:3',2'-*d*]thiophene **3c**: Prepared following the general procedure from **2** (86.3 mg, 0.25 mmol, 1.0 equiv.) and boronic acid **S1c** (166.5 mg, 0.75 mmol, 3.0 equiv.). The column chromatography was eluted with Hexane/EtOAc (10/1 (v/v)); **3c** was obtained as a yellow solid (52 mg, 38%).
^{1}H-NMR (300 MHz, $CDCl_3$): δ = 7.94 (s, 2H), 7.66 (td, J = 7.8, 1.6 Hz, 4H), 7.54 – 7.45 (m, 4H), 7.39 (ddd, J = 15.6, 7.8, 1.7 Hz, 4H), 7.34 – 7.29 (m, 6H). **^{13}C{^{1}H}-NMR** (101 MHz, $CDCl_3$) δ = 144.9 (2Cq), 139.2 (2Cq), 138.8 (2Cq), 136.2 (2Cq), 133.9 (2CH), 131.6 (4CH), 129.0 (2CH), 128.9 (2CH), 128.61 (2CH), 128.59 (4CH), 127.5 (2CH), 123.3 (2Cq), 120.7 (2Cq), 118.4 (2CH), 94.5 (2C≡C), 89.5 (2C≡C).

IR (ATR): $\tilde{\nu}$ = 3055, 3022, 2213, 1955, 1883, 1806, 1593, 1571, 1560, 1528, 1490, 1472, 1440, 1363, 1328, 1278, 1201, 1176, 1158, 1095, 1068, 1047, 1026, 999, 982, 946, 909, 868, 820, 747, 712, 686, 664, 650, 623, 596, 578, 548, 531, 511, 490, 458, 434, 423 cm^{-1}

HRMS-ESI (m/z) calculated for $C_{36}H_{20}NaS_3^+$ [M+Na]$^+$: 571.0619; found, 571.0621.

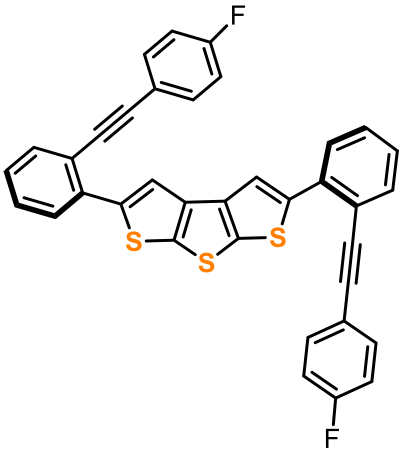

2,5-bis(2-((4-fluorophenyl)ethynyl)phenyl)dithieno[2,3-*b*:3',2'-*d*]thiophene **3d**: Prepared following the general procedure from **2** (86.3 mg, 0.25 mmol, 1.0 equiv.) and boronic acid **S1d** (180 mg, 0.75 mmol, 3.0 equiv.). The column chromatography was eluted with Hexane/EtOAc (20/1 (v/v)); **3d** was obtained as a yellow solid (46 mg, 32%). **^{1}H-NMR** (300 MHz, $CDCl_3$): δ = 7.90 (s, 2H), 7.69 – 7.61 (m, 4H), 7.49 – 7.42 (m, 4H), 7.41 – 7.28 (m, 4H), 7.00 (t, *J* = 8.8 Hz, 4H).**^{13}C{^{1}H}-NMR** (101 MHz, $CDCl_3$) δ = 162.7 (d, *J* = 250 Hz, 2Cq), 144.5 (2Cq), 139.2 (2Cq), 138.7 (2Cq), 136.1 (2Cq), 133.9 (2CH), 133.5 (2CH), 133.4 (2CH), 129.0 (d, *J* = 7 Hz, 4CH), 127.6 (2CH), 120.6 (2Cq), 119.4 (d, *J* = 4 Hz, 2Cq), 118.3 (2CH), 115.9 (d, *J* = 22 Hz, 4CH), 93.3 (2C≡C), 89.2 (2C≡C).

^{19}F-NMR (282 MHz, $CDCl_3$) δ = -110.38 (s).

IR (ATR): $\tilde{\nu}$ = 2026, 2017, 1926, 1900, 1614, 1600, 1558, 1505, 1474, 1453, 1438, 1428, 1402, 1374, 1331, 1263, 1224, 1169, 1153, 1091, 1065, 1048, 1013, 944, 832, 814, 795, 753, 699, 663, 627, 587, 565, 531, 518, 492, 480, 470, 454, 439, 424,409 cm^{-1}

HRMS-ESI (m/z) calculated for $C_{36}H_{18}F_2NaS_3^+$ $[M+Na]^+$: 607.0431; found, 607.0421.

Melting point: 225–226 °C

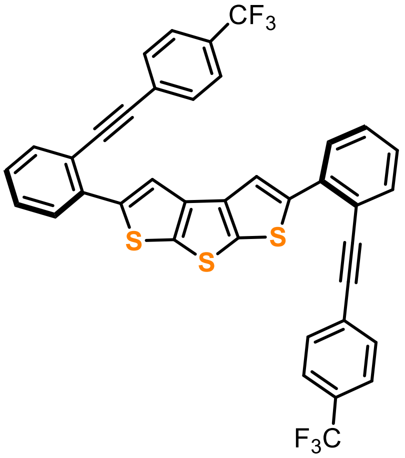

2,5-bis(2-((4-(trifluoromethyl)phenyl)ethynyl)phenyl)dithieno[2,3-*b*:3',2'-*d*]thiophene **3e**: Prepared following the general procedure from **2** (86.3 mg, 0.25 mmol, 1.0 equiv.) and boronic acid **S1e** (217.5 mg, 0.75 mmol, 3.0 equiv.). The column chromatography was eluted with Hexane/DCM (10/1 (v/v)); **3e** was obtained as a yellow solid (38 mg, 22%). **^{1}H-NMR** (400 MHz, $CDCl_3$): δ = 7.90 (s, 2H), 7.69 (dd, *J* = 7.8, 1.5 Hz, 2H), 7.65 (dd, *J* = 7.8, 1.4 Hz, 2H), 7.58 – 7.52 (m, 8H), 7.44 (td, *J* = 7.6, 1.5 Hz, 2H), 7.35 (td, *J* = 7.6, 1.3 Hz, 2H).**^{13}C{^{1}H}-NMR** (101 MHz, $CDCl_3$) δ = 144.8 (2Cq), 139.3 (2Cq), 138.7 (2Cq), 136.4 (2Cq), 134.1 (2CH), 131.7 (4CH), 130.1 (q, *J* = 33 Hz, 2Cq), 129.5 (2CH), 129.1 (2CH), 127.7 (2CH), 127.1 (2Cq), 125.5 (q, *J* = 4 Hz, 4CH), 124.0 (q, *J* = 272 Hz, 2Cq), 120.1 (2Cq), 118.4 (2CH), 92.8 (2C≡C), 91.9 (2C≡C).

^{19}F-NMR (377 MHz, CDCl3) δ = -62.78 (s).

IR (ATR): $\tilde{\nu}$ = 2215, 1921, 1613, 1595, 1571, 1560, 1515, 1494, 1475, 1454, 1441, 1405, 1362, 1320, 1259, 1163, 1122, 1102, 1064, 1016, 949, 913, 837, 811, 747, 711, 687, 650, 593, 555, 530, 513, 492, 460, 439, 408 cm^{-1}

HRMS-ESI (m/z) calculated for $C_{38}H_{18}F_6NaS_3^+$ $[M+Na]^+$: 707.0367; found, 707.0362.

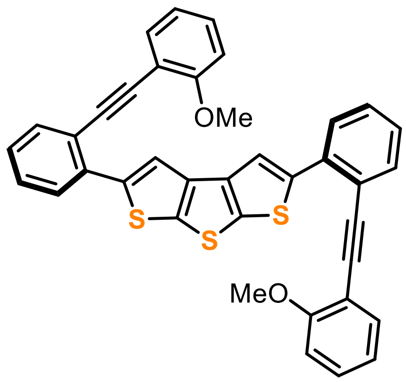

2,5-bis(2-((2-methoxyphenyl)ethynyl)phenyl)dithieno[2,3-*b*:3',2'-*d*]thiophene **3f**: Prepared following the general procedure from **2** (86.3 mg, 0.25 mmol, 1.0 equiv.) and boronic acid **S1f** (189.1 mg, 0.75 mmol, 3.0 equiv.). The column chromatography was eluted with Hexane/DCM (5/1 (v/v)); **3f** was obtained as a yellow solid (81 mg, 53%).

^{1}H-NMR (300 MHz, $CDCl_3$): δ = 8.15 (s, 2H), 7.70 (d, J = 5.8 Hz, 2H), 7.62 (d, J = 9.2 Hz, 2H), 7.42 (d, J = 7.6 Hz, 2H), 7.37 (t, J = 7.5 Hz, 2H), 7.30 (t, J = 6.7 Hz, 2H), 7.25 – 7.18 (m, 2H), 6.86 (t, J = 7.5 Hz, 2H), 6.78 (d, J = 8.4 Hz, 2H), 3.76 (s, 6H). **^{13}C{^{1}H}-NMR** (101 MHz, $CDCl_3$) δ = 160.2 (2Cq), 144.8 (2Cq), 139.2 (2Cq), 138.8 (2Cq), 135.8 (2Cq), 134.2 (2CH), 133.4 (2CH), 130.1 (2CH), 128.9 (2CH), 128.7 (2CH), 127.3 (2CH), 121.0 (2Cq), 120.6 (2CH), 118.9 (2CH), 112.5 (2Cq), 110.8 (2CH), 93.3 (2C≡C), 91.2 (2C≡C), 56.0 ($2OCH_3$).

IR (ATR): $\tilde{\nu}$ = 3057, 2934, 2833, 2211, 1899, 1592, 1573, 1494, 1474, 1432, 1362, 1327, 1297, 1275, 1242, 1203, 1180, 1160, 1112, 1096, 1045, 1021, 944, 934, 910, 866, 848, 826, 783, 745, 704, 682, 663, 649, 587, 569, 538, 518, 460 cm^{-1}

HRMS-ESI (m/z) calculated for $C_{38}H_{25}O_2S_3^+$ $[M+H]^+$: 609.1011; found, 609.1008.

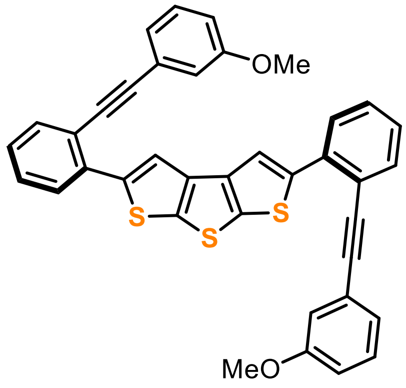

2,5-bis(2-((3-methoxyphenyl)ethynyl)phenyl)dithieno[2,3-*b*:3',2'-*d*]thiophene **3g**: Prepared following the general procedure from **2** (86.3 mg, 0.25 mmol, 1.0 equiv.) and boronic acid **S1g** (189.1 mg, 0.75 mmol, 3.0 equiv.). The column chromatography was eluted with Hexane/EtOAc (10/1 (v/v)); **3g** was obtained as a yellow solid (56 mg, 37%).

^{1}H-NMR (300 MHz, CD_2Cl_2): δ = 7.98 (s, 2H), 7.70 (d, J = 1.3 Hz, 2H), 7.67 (d, J = 1.7 Hz, 2H), 7.43 (td, J = 7.5, 1.7 Hz, 2H), 7.36 (td, J = 7.4, 1.6 Hz, 2H), 7.23 (t, J = 7.9 Hz, 2H), 7.09 (dt, J = 7.6, 1.2 Hz, 2H), 7.01 (dd, J = 2.7, 1.4 Hz, 2H), 6.87 (ddd, J = 8.4, 2.7, 1.1 Hz, 2H), 3.71 (s, 6H). **^{13}C{^{1}H}-NMR** (101 MHz, CD_2Cl_2) δ = 159.9 (2Cq), 145.2 (2Cq), 139.6 (2Cq), 139.0 (2Cq), 136.4 (2Cq), 134.1 (2CH), 130.0 (2CH), 129.3 (2CH), 129.2 (2CH), 128.0 (2CH), 124.5 (2Cq), 124.1 (2CH), 120.9 (2Cq), 118.9 (2CH), 116.6 (2CH), 115.4 (2CH), 94.5 (2C≡C), 89.4 (2C≡C), 55.6 ($2OCH_3$).

IR (ATR): $\tilde{\nu}$ = 2996, 2934, 2207, 1921, 1744, 1661, 1596, 1530, 1485, 1446, 1431, 1361, 1323, 1282, 1259, 1221, 1187, 1138, 1126, 1095, 1047, 1040, 992, 950, 930, 919, 903, 875, 856, 843, 833, 826, 782, 773, 750, 696, 683, 589, 574, 564, 523, 502, 457 cm^{-1}

HRMS-ESI (m/z) calculated for $C_{38}H_{25}O_2S_3^+$ $[M+H]^+$: 609.1011; found, 609.1016.

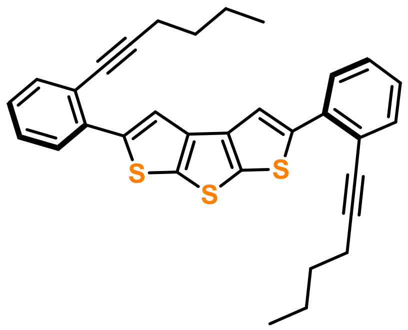

2,5-bis(2-(hex-1-yn-1-yl)phenyl)dithieno[2,3-*b*:3',2'-*d*]thiophene **3h**: Prepared following the general procedure from **2** (86.3 mg, 0.25 mmol, 1.0 equiv.) and boronic acid **S1h** (151.5 mg, 0.75 mmol, 3.0 equiv.). The column chromatography was eluted with Hexane/DCM (5/1 (v/v)); **3h** was obtained as a yellow oil (38 mg, 30%).

^{1}H-NMR (300 MHz, $CDCl_3$): *δ* = 7.85 (s, 2H), 7.57 (dd, *J* = 7.9, 1.4 Hz, 2H), 7.53 (dd, *J* = 7.7, 1.4 Hz, 2H), 7.32 (td, *J* = 7.6, 1.7 Hz, 2H), 7.28 – 7.20 (m, 2H), 2.46 (t, *J* = 7.0 Hz, 4H), 1.66 – 1.55 (m, 4H), 1.52 – 1.37 (m, 4H), 0.89 (t, *J* = 7.3 Hz, 6H).

^{13}C{^{1}H}-NMR (101 MHz, $CDCl_3$) δ = 145.1 (2Cq), 138.7 (2Cq), 136.0 (2Cq), 134.1 (2CH), 128.9 (2CH), 128.1 (2CH), 127.4 (2CH), 121.7 (2Cq), 118.3 (2CH), 95.9 (2C≡C), 80.4 (2C≡C), 30.60 ($2CH_2$), 22.3 ($2CH_2$), 19.7 ($2CH_2$), 13.8 ($2CH_3$) (One Cq signal was not observed).

IR (ATR): $\tilde{\nu}$ = 2927, 2869, 2857, 2225, 1592, 1559, 1522, 1494, 1478, 1464, 1438, 1426, 1376, 1363, 1326, 1237, 1200, 1157, 1103, 905, 868, 826, 754, 728, 711, 682, 663, 654, 648, 598, 571, 567, 555, 548, 542, 536, 513, 506, 498, 463, 455, 449, 434, 420, 415, 402 cm^{-1}

HRMS-ESI (m/z) calculated for $C_{32}H_{29}S_3^+$ $[M+H]^+$: 509.1426; found, 509.1428.

Synthesis and characterization of helicenes

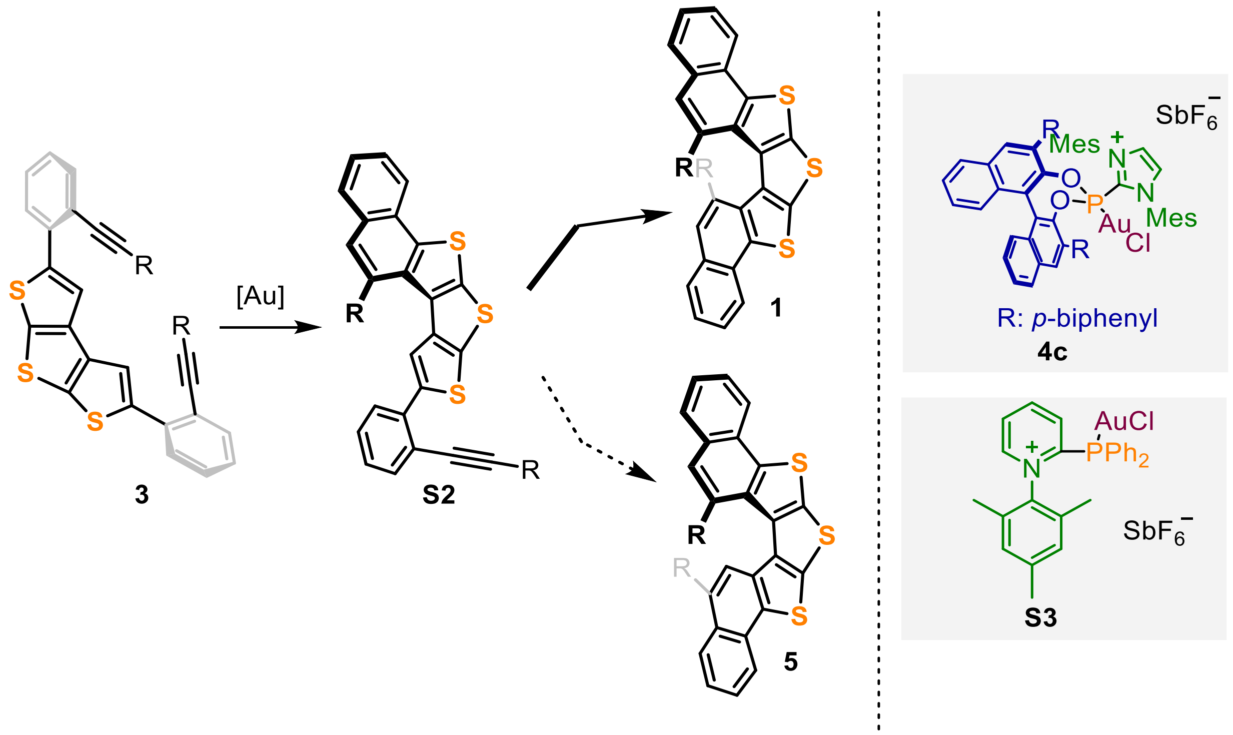

Scheme S 4: Synthesis of thiahelicenes.

General procedure for the synthesis of thiahelicenes

To a flame-dried Schlenk flask, the chiral gold catalyst **4c** (2.9 mg, 2 µmol, 10 mol%) was added as solid. Then, the flask was fitted with a silicon septum and evacuated and back-filled with argon twice. Then, a solution of alkyne **3** (20 µmol, 0.05 M) in dry DCM were added to the flask and the reaction mixture allowed to cool down to –20 °C. Then, a freshly prepared solution of $AgSbF_6$ (10 mol%, 0.05 M in DCM) was added dropwise. After stirring for 48 hours at the indicated temperatures, the mixtures were filtered through a silica plug eluting with DCM and the solvent removed under reduced pressure to afford the desired product. The enantiomeric excesses were determined by CSP-HPLC.

Racemic references of the helicenes were obtained employing gold catalyst **S3** (r.t., 24 h) under otherwise identical conditions. In some of these reactions monocyclised side-products **S2** were obtained:

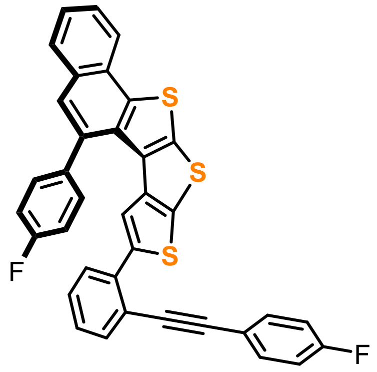

S2d: Prepared following the general procedure from **3d** (11.6 mg, 20 µmol), the product was obtained as a colorless solid in 38% yield after purification by preparative HPLC (acetonitrile:H_2O 95:5→100:0 over 5 min, flow rate 1 mL/min at 22 °C).

^{1}H-NMR (300 MHz, CD_2Cl_2): δ = 8.15 (d, *J* = 8.1 Hz, 1H), 7.97 (d, *J* = 7.6 Hz, 1H), 7.68 (s, 1H), 7.67 – 7.56 (m, 3H), 7.55 – 7.44 (m, 5H), 7.41 (dd, *J* = 7.2, 2.0 Hz, 1H), 7.36 – 7.28 (m, 2H), 7.28 – 7.20 (m, 2H), 7.10 – 6.99 (m, 2H).**^{13}C{APT}-NMR** (126 MHz, CD_2Cl_2) δ = 163.3 (d, *J* = 247 Hz, Cq), 163.1 (d, *J* = 249 Hz, Cq), 142.8 (Cq), 141.4 (Cq), 140.3 (Cq), 139.9 (Cq), 139.0 (Cq), 138.9 (d, *J* = 3 Hz,

Cq), 136.4 (Cq), 135.2 (Cq), 134.9 (Cq), 134.0 (CH), 133.8 (d, J = 8 Hz, 2CH); 132.2 (d, J = 8 Hz, 2CH), 130.4 (Cq), 129.7 (Cq), 129.2 (CH), 129.1 (CH), 128.9 (CH), 128.8 (Cq), 127.8 (CH), 127.5 (CH), 127.4 (CH), 126.7 (CH), 123.2 (CH), 121.2 (CH), 120.3 (Cq), 119.8 (d, J = 4 Hz, Cq), 116.2 (d, J = 2 Hz, 2CH), 116.0 (d, J = 3 Hz, 2CH), 93.7 (C≡C), 89.3 (C≡C).

^{19}F-NMR (282 MHz, CD_2Cl_2) δ = -111.11 (s), -115.07 (s).

IR (ATR): $\tilde{\nu}$ = 3062, 2922, 2851, 2212, 2085, 1895, 1874, 1711, 1648, 1619, 1600, 1556, 1504, 1495, 1444, 1403, 1376, 1358, 1338, 1318, 1304, 1260, 1223, 1197, 1154, 1136, 1090, 1055, 1015, 975, 955, 914, 877, 826, 799, 787, 750, 740, 686, 665, 630, 601, 585, 560, 530, 516, 504, 491, 460, 435 cm^{-1}

HRMS-EI (m/z) calculated for $C_{36}H_{18}F_2S_3^+$ $[M]^+$: 584.0533; found, 584.0536.

Melting point: 219–220°C

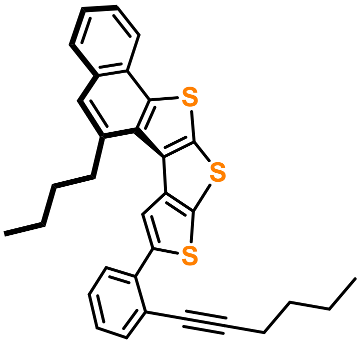

S2h: Prepared following the general procedure from **3h** (10.2 mg, 20 µmol), the product was obtained as a yellow solid in 52% yield after purification by preparative HPLC (acetonitrile:THF 95:5→85:15 over 20 min, flow rate 1 mL/min at 22 °C).

^{1}H-NMR (300 MHz, CD_2Cl_2): δ = 8.17 (s, 1H), 8.10 – 8.03 (m, 1H), 7.88 (dd, J = 6.9, 2.6 Hz, 1H), 7.60 (s, 1H), 7.57 (d, J = 6.5 Hz, 2H), 7.54 – 7.47 (m, 2H), 7.36 (td, J = 7.6, 1.7 Hz, 1H), 7.29 (dd, J = 7.6, 1.5 Hz, 1H), 3.54 (t, J = 7.6 Hz, 2H), 2.46 (t, J = 7.1 Hz, 2H), 1.98 – 1.86 (m, 2H), 1.63 – 1.56 (m, 2H), 1.50 – 1.34 (m, 2H), 0.97 (t, J = 7.3 Hz, 3H), 0.86 (t, J = 7.3 Hz, 3H).

^{13}C{APT}-NMR (101 MHz, $CDCl_3$) δ = 143.8 (Cq), 141.1 (Cq), 139.6 (Cq), 139.3 (Cq), 138.9 (Cq), 136.1 (Cq), 135.7 (Cq), 134.8 (Cq), 134.3 (CH), 130.9 (Cq), 130.6 (Cq), 129.0 (CH), 128.2 (Cq), 128.12 (CH), 128.09 (CH), 127.5 (CH), 126.2 (CH), 125.9 (CH), 125.2 (CH), 123.0 (CH), 121.9 (Cq), 121.6 (CH), 96.0 (C≡C), 80.3 (C≡C), 36.3 (CH_2), 33.2 (CH_2), 30.7 (CH_2), 22.3 (CH_2), 22.2 (CH_2), 19.8 (CH_2), 14.3 (CH_3), 13.8 (CH_3).

IR (ATR): $\tilde{\nu}$ = 2957, 2925, 2868, 2223, 1707, 1624, 1591, 1556, 1516, 1496, 1478, 1454, 1441, 1378, 1350, 1342, 1327, 1307, 1264, 1242, 1219, 1202, 1162, 1128, 1102, 1076, 1047, 943, 931, 872, 841, 821, 741, 673, 651, 638, 574, 538, 523, 510, 487, 457, 416 cm^{-1}

HRMS-ESI (m/z) calculated for $C_{32}H_{29}S_3^+$ $[M+H]^+$: 509.1426; found, 509.1425.

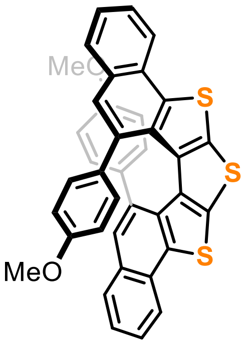

Trithia[5]helicene (+)-1a: Prepared following the general procedure from **3a** (12.2 mg, 20 µmol). The product was isolated as a yellow solid in 96% yield (11.7 mg, 19.2 µmol) with 91% *ee*. Using achiral gold catalyst **S3**, the racemic product was obtained in 62% yield after purification by preparative HPLC (acetonitrile:H_2O 95:5→100:0 over 5 min, flow rate 1 mL/min at 22 °C).

^{1}H-NMR (300 MHz, $CDCl_3$): δ = 8.04 (d, J = 8.3 Hz, 2H), 7.83 (d, J = 7.9 Hz, 2H), 7.57 – 7.45 (m, 4H), 7.44 (s, 2H), 7.11 (d, J = 8.5 Hz, 4H), 6.31 (d, J = 7.1 Hz, 4H), 3.21 (s, 6H).**^{13}C{^{1}H}-NMR** (101 MHz, $CDCl_3$) δ = 159.0 (2Cq), 140.4 (2Cq), 137.1 (2Cq), 136.7 (2Cq), 134.3 (2Cq), 131.6 (2Cq), 130.9 (2Cq), 130.7 (4CH), 129.9 (2Cq), 128.7 (2CH), 127.7 (2Cq), 126.2 (2CH), 125.8 (2CH), 125.0 (2CH), 122.7 (2CH), 112.3 (4CH), 55.2 (2OCH_3).

IR (ATR): $\tilde{\nu}$ = 2931, 2832, 1604, 1569, 1546, 1510, 1492, 1459, 1448, 1439, 1416, 1400, 1369, 1348, 1339, 1315, 1283, 1244, 1211, 1175, 1144, 1112, 1086, 1024, 1002, 971, 946, 913, 893, 880, 853, 821, 808, 798, 775, 760, 743, 727, 708, 691, 659, 640, 629, 547, 522, 509, 470, 457, 422, 409 cm^{-1}

HRMS-ESI (m/z) calculated for $C_{38}H_{25}O_2S_3^+$ $[M+H]^+$: 609.1011; found, 609.0999.

$[\alpha]_{21}^{D}$: +1376° (c = 0.03 in DCM) for 91% *ee*.

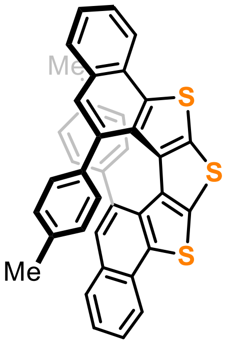

Trithia[5]helicene (+)-1b: Prepared following the general procedure from **3b** (11.5 mg, 20 µmol). The product was isolated as a yellow solid in 95% yield (10.9 mg, 19 µmol) with 89% *ee*. Using achiral gold catalyst **S3**, the racemic product was obtained in 56% yield after purification by preparative HPLC (acetonitrile:H_2O 95:5→100:0 over 5 min, flow rate 1 mL/min at 22 °C).

^{1}H-NMR (300 MHz, $CDCl_3$): δ = 8.05 (d, J = 6.6 Hz, 2H), 7.81 (d, J = 7.2 Hz, 2H), 7.58 – 7.45 (m, 4H), 7.43 (s, 2H), 7.06 (d, J = 8.0 Hz, 4H), 6.59 (d, J = 7.6 Hz, 4H), 1.91 (s, 6H).**^{13}C{^{1}H}-NMR** (101 MHz, $CDCl_3$) δ = 140.3 (2Cq), 137.2 (2Cq), 136.8 (4Cq), 136.0 (2Cq), 134.6 (2Cq), 130.9 (2Cq), 129.9 (2Cq), 129.5 (4CH), 128.6 (2CH), 127.9 (2Cq), 127.4 (4CH), 126.2 (2CH), 125.8 (2CH), 125.4 (2CH), 122.8 (2CH), 20.8 (2CH_3).

IR (ATR): $\tilde{\nu}$ = 3045, 2918, 2856, 1904, 1681, 1651, 1579, 1546, 1513, 1493, 1446, 1400, 1370, 1348, 1312, 1279, 1256, 1219, 1188, 1156, 1143, 1113, 1089, 1026, 1008, 976, 965, 838, 815, 796, 762, 737, 722, 642, 629, 602, 586, 568, 555, 534, 518, 502, 491, 453, 411 cm^{-1}

HRMS-EI (m/z) calculated for $C_{38}H_{24}S_3^+$ $[M]^+$: 576.1035; found, 576.1037.

$[\alpha]_{22}^{D}$: +1229° (c = 0.045 in DCM) for 89% *ee*.

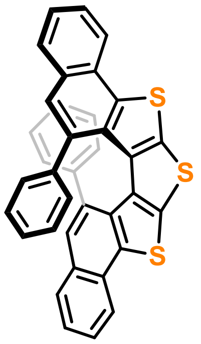

Trithia[5]helicene (+)-1c: Prepared following the general procedure from **3c** (11 mg, 20 µmol). The product was isolated as a yellow solid in 93% yield (10.2 mg, 18.6 µmol) with 88% *ee*. Using achiral gold catalyst **S3**, the racemic product was obtained in 45% yield after purification by preparative HPLC (acetonitrile:H_2O 95:5→100:0 over 5 min, flow rate 1 mL/min at 22 °C).

^{1}H-NMR (300 MHz, $CDCl_3$): δ = 8.08 (ddd, *J* = 8.1, 1.5, 0.7 Hz, 2H), 7.89 – 7.81 (m, 2H), 7.56 (ddd, *J* = 8.1, 7.0, 1.5 Hz, 2H), 7.49 (ddd, *J* = 8.3, 7.0, 1.4 Hz, 2H), 7.46 (s, 2H), 7.29 – 7.11 (m, 4H), 6.94 – 6.73 (m, 6H).**^{13}C{^{1}H}-NMR** (101 MHz, $CDCl_3$) δ = 140.8 (2Cq), 139.2 (2Cq), 138.2 (2Cq), 136.8 (2Cq), 135.1 (2Cq), 131.0 (2Cq), 130.0 (2Cq), 129.8 (4CH), 129.1 (2CH), 128.2 (2Cq), 127.2 (2CH), 127.1 (4CH), 126.8 (2CH), 126.5 (2CH), 126.2 (2CH), 123.0 (2CH).

IR (ATR): $\tilde{\nu}$ = 3052, 2919, 1947, 1691, 1597, 1580, 1548, 1489, 1445, 1405, 1372, 1349, 1310, 1254, 1212, 1181, 1159, 1144, 1077, 1025, 1008, 978, 954, 946, 913, 887, 848, 830, 790, 767, 857, 744, 726, 709, 691, 651, 622, 606, 589, 568, 554, 519, 500, 481, 458, 441, 421, 407 cm^{-1}

HRMS-ESI (m/z) calculated for $C_{36}H_{20}NaS_3^+$ [M]$^+$: 571.0619; found, 571.0612.

$[\alpha]_{22}^{D}$: +1160° (*c* = 0.04 in DCM) for 88% *ee*.

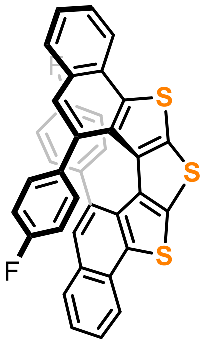

Trithia[5]helicene (+)-1d: Prepared following the general procedure from **3d** (11.6 mg, 20 µmol). The product was isolated as a yellow solid in 93% yield (10.7 mg, 18.4 µmol) with 84% *ee*. Using achiral gold catalyst **S3**, the racemic product was obtained in 50% yield after purification by preparative HPLC (acetonitrile:H_2O 95:5→100:0 over 5 min, flow rate 1 mL/min at 22 °C).

^{1}H-NMR (300 MHz, CD_2Cl_2): δ = 8.09 (d, *J* = 8.1 Hz, 2H), 7.88 (d, *J* = 7.9 Hz, 2H), 7.59 (td, *J* = 8.2, 7.6, 1.6 Hz, 2H), 7.56 – 7.49 (m, 2H), 7.48 (s, 2H), 7.25 – 7.10 (m, 4H), 6.66 – 6.40 (m, 4H).**^{13}C{^{1}H}-NMR** (126 MHz, CD_2Cl_2) δ = 162.4 (d, *J* = 247 Hz, 2Cq), 141.2 (2Cq), 138.5 (2Cq), 136.3 (2Cq), 135.5 (d, *J* = 3 Hz, 2Cq), 133.7 (2Cq), 131.4 (d, *J* = 8 Hz, 4CH), 131.0 (2Cq), 129.6 (2Cq), 129.1 (2CH), 128.2 (2Cq), 127.1 (2CH), 126.5 (2CH), 126.3 (2CH), 123.0 (2CH), 114.0 (d, *J* = 21 Hz, 4CH).

^{19}F-NMR (282 MHz, CD_2Cl_2) δ = -116.63 (s).

IR (ATR): $\tilde{\nu}$ = 3049, 2923, 2848, 1600, 1508, 1492, 1446, 1399, 1345, 1337, 1310, 1254, 1219, 1158, 1097, 1024, 1009, 959, 945, 912, 882, 856, 830, 818, 804, 777, 766, 740, 726, 690, 639, 627, 600, 587, 567, 554, 530, 521, 503, 493, 458, 444, 437, 419, 408 cm^{-1}

HRMS-EI (m/z) calculated for $C_{36}H_{18}F_2S_3^+$ [M]$^+$: 584.0533; found, 584.0536.

$[\alpha]_{22}^{D}$: +886° (*c* = 0.045 in DCM) for 84% *ee*.

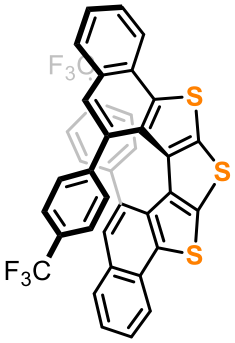

Trithia[5]helicene (+)-1e: Prepared following the general procedure from **3e** (13.7 mg, 20 µmol). The product was isolated as a yellow solid in 94% yield (12.9 mg, 18.8 µmol) with 91% *ee*. Using achiral gold catalyst **S3**, the racemic product was obtained in 75% yield after recrystallization from hexane and dichloromethane.

^{1}H-NMR (300 MHz, CD_2Cl_2): δ = 8.10 (d, J = 8.1 Hz, 2H), 7.86 (d, J = 7.3 Hz, 2H), 7.62 (ddd, J = 8.3, 7.0, 1.4 Hz, 2H), 7.57 – 7.47 (m, 4H), 7.40 – 7.20 (m, 4H), 7.17 – 6.92 (m, 4H). **^{13}C{^{1}H}-NMR** (101 MHz, $CDCl_3$) δ = 142.3 (2Cq), 141.4 (2Cq), 138.8 (2Cq), 136.0 (2Cq), 132.7 (2Cq), 130.8 (2Cq), 130.1 (4CH), 129.4 (2CH), 129.2 (2Cq), 128.8 (2Cq), 128.6 (2Cq), 127.6 (2CH), 126.7 (2CH), 126.6 (2CH), 140.3 (q, J = 273 Hz, 2Cq), 124.1 (q, J = 4 Hz, 4CH), 122.9 (2CH).

^{19}F-NMR (282 MHz, CD_2Cl_2) δ = -63.09 (s).

IR (ATR): $\tilde{\nu}$ = 3058, 1922, 1614, 1585, 1547, 1494, 1444, 1417, 1400, 1322, 1257, 1165, 1119, 1108, 1066, 1016, 1009, 973, 946, 913, 886, 856, 839, 770, 760, 749, 739, 720, 710, 696, 665, 638, 618, 603, 587, 578, 560, 552, 519, 496, 483, 471, 459, 433, 409 cm^{-1}

HRMS-ESI (m/z) calculated for $C_{38}H_{19}F_6S_3^+$ $[M+H]^+$: 685.0548; found, 685.0585.

Melting point: 348–349°C

$[\alpha]_{22}^{D}$: +1307° (c = 0.034 in DCM) for 91% *ee*.

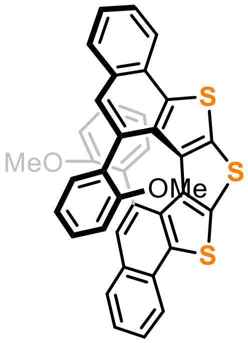

Trithia[5]helicene (+)-1f: Prepared following the general procedure from **3f** (12.2 mg, 20 µmol). The product was isolated as a yellow solid in 92% yield (11.2 mg, 18.4 µmol) with 87% *ee*. Using achiral gold catalyst **S3**, the racemic product was obtained in 70% yield after recrystallization from acetonitrile and water.

^{1}H-NMR (300 MHz, $CDCl_3$): δ = 8.04 (d, J = 8.4 Hz, 2H), 7.82 (d, J = 7.9 Hz, 2H), 7.59 – 7.39 (m, 8H), 6.89 (t, J = 7.7 Hz, 2H), 6.77 (td, J = 7.8, 7.4, 1.8 Hz, 2H), 6.01 (d, J = 8.2 Hz, 2H), 2.83 (s, 6H). **^{13}C{^{1}H}-NMR** (101 MHz, $CDCl_3$) δ = 156.0 (2Cq), 138.8 (2Cq), 136.0 (2Cq), 133.3 (2CH), 132.2 (2Cq), 131.3 (2Cq), 130.8 (2Cq), 129.0 (2CH), 128.8 (2CH), 128.2 (2Cq), 127.8 (2Cq), 127.2 (2CH), 126.3 (2CH), 125.4 (2CH), 122.8 (2CH), 119.3 (2CH), 109.4 (2CH), 53.9 ($2OCH_3$).

IR (ATR): $\tilde{\nu}$ = 3050, 2984, 2931, 2826, 1599, 1581, 1492, 1460, 1431, 1412, 1372, 1358, 1339, 1312, 1300, 1284, 1250, 1178, 1163, 1132, 1111, 1084, 1026, 1006, 995, 971, 945, 935, 914, 886, 854, 838, 796, 765, 742, 711, 696, 664, 647, 618, 609, 598, 580, 525, 518, 479, 438, 420, 411 cm^{-1}

HRMS-ESI (m/z) calculated for $C_{38}H_{25}O_2S_3^+$ $[M+H]^+$: 609.1011; found, 609.1010.

Melting point: 370–372°C

$[\alpha]_{23}^{D}$: +1035° (c = 0.06 in DCM) for 87% *ee*.

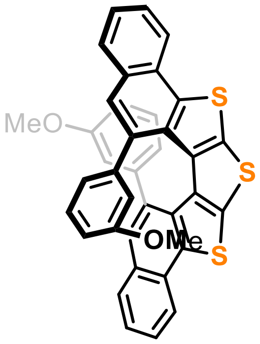

Trithia[5]helicene (+)-1g: Prepared following the general procedure from **3f** (12.2 mg, 20 µmol). The product was isolated as a yellow solid in 95% yield (11.6 mg, 19 µmol) with 10% *ee*. Using achiral gold catalyst **S3**, the racemic product was obtained in 40% yield after purification by preparative HPLC (acetonitrile:H_2O 90:10→100:0 over 10 min, flow rate 1 mL/min at 22 °C).

^{1}H-NMR (300 MHz, $CDCl_3$): δ = 8.06 (d, J = 8.0 Hz, 2H), 7.89 – 7.80 (m, 2H), 7.56 (dd, J = 6.9, 1.4 Hz, 2H), 7.52 (s, 2H), 7.48 (ddd, J = 8.2, 6.9, 1.4 Hz, 2H), 6.88 (bs, 4H), 6.64 (bs, 2H), 6.33 (ddd, J = 7.8, 2.6, 1.3 Hz, 2H), 3.21 (s, 6H).**^{13}C{^{1}H}-NMR** (101 MHz, $CDCl_3$) δ = 158.1 (2Cq), 140.6 (2Cq), 140.1 (2Cq), 137.5 (2Cq), 136.7 (2Cq), 134.5 (2Cq), 130.8 (2Cq), 129.9 (2Cq), 128.9 (2CH), 128.04 (2Cq), 127.98 (2CH), 126.6 (2CH), 126.2 (2CH), 126.0 (2CH), 122.8 (2CH), 122.3 (2CH), 115.2 (2CH), 112.9 (2CH), 54.7 ($2OCH_3$).

IR (ATR): $\tilde{\nu}$ = 3046, 2927, 2838, 1590, 1572, 1547, 1488, 1455, 1427, 1400, 1369, 1322, 1282, 1251, 1220, 1207, 1198, 1178, 1160, 1143, 1091, 1042, 1027, 1008, 997, 972, 935, 902, 881, 867, 843, 805, 785, 757, 741, 714, 694, 665, 626, 602, 572, 563, 550, 532, 465, 440, 407 cm^{-1}

HRMS-ESI (m/z) calculated for $C_{38}H_{25}O_2S_3^+$ $[M+H]^+$: 609.1011; found, 609.1012.

$[\alpha]^D_{23}$: +95° (c = 0.05 in DCM) for 10% *ee*.

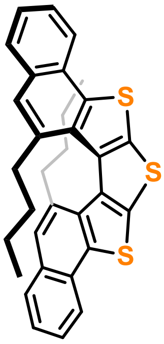

Trithia[5]helicene (+)-1h: Prepared from **3h** (10.2 mg, 20 µmol) following the general procedure but prolonging the reaction time to 72 h. The product was isolated as a yellow solid in 90% yield (9.2 mg, 18 µmol) with 59% *ee*. Using achiral gold catalyst **S3**, the racemic product was obtained in 40% yield after purification by preparative HPLC (acetonitrile:THF 95:5→85:15 over 20 min, flow rate 1 mL/min at 22 °C). 58% of intermediate **S2h** was still present in the reaction mixture.

^{1}H-NMR (300 MHz, $CDCl_3$): δ = 8.11 – 8.04 (m, 2H), 7.95 – 7.88 (m, 2H), 7.63 (s, 2H), 7.54 (tt, J = 7.0, 5.2 Hz, 4H), 3.38 (ddd, J = 13.8, 9.2, 6.3 Hz, 2H), 3.06 (ddd, J = 14.4, 9.4, 5.8 Hz, 2H), 1.28 – 1.14 (m, 2H), 1.14 – 0.97 (m, 2H), 0.89 – 0.76 (m, 2H), 0.75 – 0.61 (m, 2H), 0.39 (t, J = 7.3 Hz, 6H).

^{13}C{^{1}H}-NMR (101 MHz, $CDCl_3$) δ = 140.3 (2Cq), 138.7 (2Cq), 138.3 (2Cq), 135.3 (2Cq), 132.0 (2Cq), 131.5 (2Cq), 128.2 (2CH), 128.0 (2Cq), 126.0 (2CH), 125.9 (2CH), 124.5 (2CH), 123.0 (2CH), 35.7 ($2CH_2$), 34.5 ($2CH_2$), 22.1 ($2CH_2$), 13.5 ($2CH_3$).

IR (ATR): $\tilde{\nu}$ = 2953, 2925, 2858, 1709, 1580, 1496, 1463, 1450, 1407, 1376, 1354, 1340, 1305, 1262, 1220, 1192, 1141, 975, 944, 898, 884, 844, 768, 738, 671, 646, 604, 574, 520, 485, 466, 437, 412 cm^{-1}

HRMS-ESI (m/z) calculated for $C_{32}H_{29}S_3^+$ $[M+H]^+$: 509.1426; found, 509.1425.

$[\alpha]^D_{23}$: +480° (c = 0.1 in DCM) for 59% *ee*.

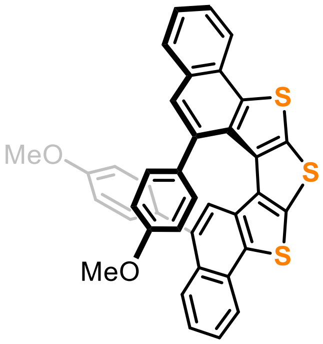

5a: Obtained following the general procedure from **3a** (12.2 mg, 20 µmol). When using the non-chiral gold catalyst **S3**, the product was obtained as a colorless solid in 36% yield after purification by preparative HPLC (acetonitrile:H_2O 95:5→100:0 over 5 min, flow rate 1 mL/min at 22 °C).

^{1}H-NMR (300 MHz, $CDCl_3$): δ = 8.12 (d, J = 8.4 Hz, 1H), 8.08 (d, J = 8.1 Hz, 1H), 7.92 (t, J = 7.6 Hz, 2H), 7.81 (s, 1H), 7.62 – 7.49 (m, 3H), 7.45 – 7.36 (m, 3H), 7.21 (d, J = 8.2 Hz, 2H), 7.06 (d, J = 8.3 Hz, 3H), 6.30 – 6.21 (m, 2H), 3.98 (s, 3H), 3.56 (s, 3H). **^{13}C{^{1}H}-NMR** (126 MHz, $CDCl_3$) δ = 159.2 (Cq), 158.9 (Cq), 142.2 (Cq), 139.7 (Cq), 139.1 (Cq), 138.9 (Cq), 136.9 (Cq), 136.1 (Cq), 135.7 (Cq), 135.3 (Cq), 134.5 (Cq), 133.1 (Cq), 131.7 (2CH), 130.99 (Cq), 130.97 (Cq), 129.2 (Cq), 129.0 (2CH), 128.8 (Cq), 128.72 (Cq), 128.65 (CH), 128.1 (Cq), 127.5 (CH), 127.0 (CH), 126.7 (CH), 126.4 (CH), 126.2 (CH), 125.6 (CH), 123.5 (CH), 123.1 (CH), 122.7 (CH), 114.9 (2CH), 113.4 (2CH), 55.6 (OCH_3), 55.2 (OCH_3).

IR (ATR): $\tilde{\nu}$ = 2927, 2832, 1606, 1510, 1462, 1452, 1401, 1382, 1331, 1302, 1290, 1242, 1213, 1174, 1107, 1092, 1033, 998, 975, 918, 900, 888, 859, 827, 802, 793, 742, 699, 678, 666, 638, 614, 600, 550, 523, 512, 499, 471, 456, 442, 424, 413 cm^{-1}

HRMS-ESI (m/z) calculated for $C_{36}H_{24}NaO_2S_3^+$ [M+Na]$^+$: 631.0831; found, 631.0840.

Melting point: 202–223°C

Synthesis of **8a**:

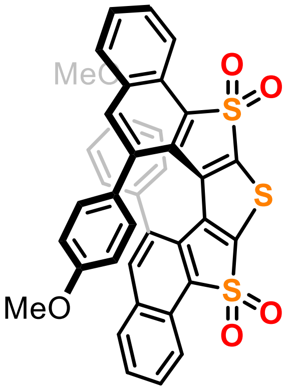

m-CPBA (contains ca. 30% water, 29 mg, 117.5 µmol, 4.7 equiv.) was added to a solution of (+)-**1a** (15.2 mg, 25 µmol, 92% ee) in dry CH_2Cl_2 (0.25 mL). The reaction mixture was stirred at –10 °C under nitrogen overnight, then poured into brine (2 × 5 mL). The aqueous phase was extracted with CH_2Cl_2 (3 × 5 mL), the combined organic phases were washed with brine, dried over Na_2SO_4, and concentrated under reduced pressure. The residue obtained was purified by HPLC (acetonitrile:H_2O 80:20→100:0 over 20 min, flow rate 1 mL/min at 22 °C) to deliver compound **8a** as a yellow solid in 63% yield (10.6 mg, 15.7 µmol) with 92% ee.

^{1}H-NMR (300 MHz, $CDCl_3$): δ = 8.30 (d, J = 7.3 Hz, 2H), 7.84 (d, J = 7.6 Hz, 2H), 7.71 (s, 2H), 7.70 – 7.63 (m, 2H), 7.62 – 7.55 (m, 2H), 7.18 (d, J = 9.0 Hz, 4H), 6.43 (d, J = 8.6 Hz, 4H), 3.04 (s, 6H).

^{13}C{^{1}H}-NMR (101 MHz, $CDCl_3$) δ = 159.7 (2Cq), 144.9 (2Cq), 139.1 (2Cq), 138.0 (2Cq), 133.9 (2Cq), 132.79 (2CH), 132.77 (2Cq), 130.9 (4CH), 129.1 (2CH), 128.8 (2CH), 128.3 (2CH), 128.2 (2Cq), 126.7 (2Cq), 124.9 (2Cq), 122.7 (2CH), 113.1 (4CH), 54.9 (2OCH_3).

IR (ATR): $\tilde{\nu}$ = 2930, 2836, 5169, 1605, 1516, 1504, 1462, 1435, 1310, 1250, 1176, 1161, 1142, 1085, 1030, 1000, 962, 953, 904, 852, 827, 773, 749, 726, 709, 673, 631, 610, 592, 585, 568, 561, 553, 538, 529, 514, 488, 480, 470, 441, 432, 418, 403 cm^{-1}

HRMS-ESI (m/z) calculated for $C_{38}H_{24}Br_2NaO_6S_3^+$ $[M+Na]^+$: 695.0627; found, 695.0624.

$[\boldsymbol{\alpha}]_{25}^{D}$: +1062° (*c* = 0.03 in DCM) for 92% *ee*

Synthesis of **9a**

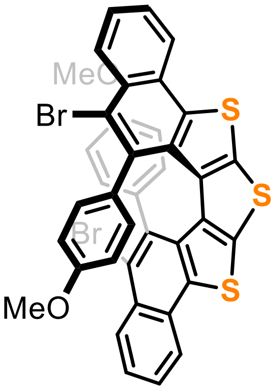

Under darkness, to a mixture of **1a** (30.4 mg, 0.05 mmol) in $CHCl_3$ (2.5 mL, 0.02 M), N-bromosuccinimide (27 mg, 0.15 mmol, 3.0 equiv.) was added in 3 portions (during one hour) at 35 °C, and the resulting suspension stirred at the same temperature for another 20 hours. Then, the reaction mixture was poured into H_2O (5 mL), the aqueous phase was extracted with CH_2Cl_2 (3 × 5 mL), the combined organic phases were dried over Na_2SO_4, filtered, and the solvent removed under reduced pressure. The residue was purified by chromatography on silica gel eluting (eluted with Hexane/EtOAc (15/1 (v/v)), affording the brominated product **9a** (23.4 mg, 30.5 µmol, 61%) as a yellow solid. The enantiopurity was determined by HPLC (92% ee).

^{1}H-NMR (400 MHz, $CDCl_3$): δ = 8.55 – 8.36 (m, 4H), 8.01 – 7.91 (m, 2H), 7.64 – 7.53 (m, 4H), 6.81 (bs, 2H), 6.21 (bs, 2H), 5.86 (bs, 2H), 3.04 (s, 6H).

^{13}C{^{1}H}-NMR (101 MHz, $CDCl_3$) δ = 158.4 (2Cq), 139.7 (2Cq), 136.8 (2Cq), 136.5 (2Cq), 135.2 (2CH), 134.6 (2Cq), 133.9 (2CH), 131.8 (2Cq), 130.3 (2Cq), 129.7 (2Cq), 129.5 (2CH), 128.5 (2Cq), 127.1 (2CH), 126.8 (2CH), 122.9 (2CH), 120.2 (2Cq), 111.4 (4CH), 54.8 ($2OCH_3$).

IR (ATR): $\tilde{\nu}$ = 3001, 2962, 2928, 2832, 1721, 1603, 1573, 1548, 1509, 1482, 1455, 1387, 1358, 1329, 1308, 1293, 1247, 1177, 1166, 1132, 1111, 1095, 1030, 999, 971, 948, 940, 905, 878, 846, 825, 814, 801, 779, 755, 742, 729, 716, 708, 693, 653, 630, 610, 600, 588, 556, 537, 518, 476, 453, 443, 419, 403 cm^{-1}

HRMS-ESI (m/z) calculated for $C_{38}H_{22}Br_2NaO_2S_3^+$ $[M+Na]^+$: 786.9041; found, 786.9020.

Melting point: 276–277 °C

$[\boldsymbol{\alpha}]_{25}^{D}$: +556° (*c* = 0.033 in DCM) for 91% *ee*

Procedure for the synthesis of **10a** and **11a**

10a: Under a nitrogen atmosphere, a mixture of (+)-**9a** (26.8 mg, 35 µmol, 1.0 equiv.), $PhB(OH)_2$ (17.2 mg, 0.14 mmol, 4.0 equiv.), Pd(dppf)Cl_2 (2.6 mg, 3.5 µmol, 10 mol%), KF (8.1 mg, 0.14 mmol, 4:0 equiv.) in toluene (0.5 mL) and methanol (0.5 mL) was stirred at 110 °C overnight using microwave reactor. After cooling down to room temperature, DCM (10 mL) was added, and the organic phase was washed with water (2 × 5 mL). The combined organic phase was dried over Na_2SO_4, filtered, and the solvent removed under reduced pressure. The residue obtained was purified by HPLC (acetonitrile:H_2O 95:5→100:0 over 5 min, flow rate 1

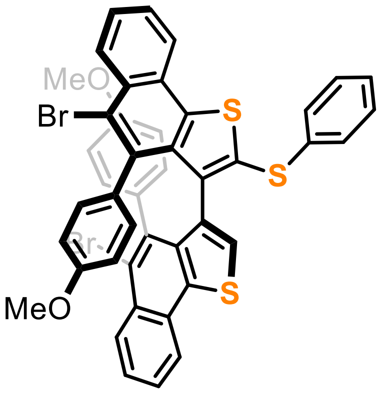

mL/min at 22 °C) to deliver compound **10a** as a white solid in 38% yield (11.2 mg, 13.3 µmol) with 90% ee.

^{1}H-NMR (300 MHz, $CDCl_3$): δ = 8.44 (ddd, J = 8.2, 5.4, 2.9 Hz, 2H), 8.06 (dt, J = 7.6, 2.7 Hz, 1H), 7.95 – 7.89 (m, 1H), 7.69 – 7.54 (m, 4H), 7.44 – 7.27 (m, 5H), 7.04 – 6.94 (m, 2H), 6.92 (s, 1H), 6.73 (dd, J = 8.5, 2.3 Hz, 1H), 6.51 (dd, J = 8.5, 2.3 Hz, 1H), 6.44 (dd, J = 8.5, 2.7 Hz, 1H), 6.39 (dd, J = 8.4, 2.7 Hz, 1H), 5.72 (t, J = 3.2 Hz, 1H), 5.69 (t, J = 3.1 Hz, 1H), 3.16 (s, 3H), 3.14 (s, 3H).

^{13}C{^{1}H}-NMR (126 MHz, $CDCl_3$) δ = 157.7 (Cq), 157.6 (Cq), 139.0 (Cq), 138.4 (Cq), 137.7 (Cq), 137.12 (Cq), 137.06 (Cq), 136.7 (Cq), 136.2 (Cq), 135.9 (Cq), 133.3 (Cq), 131.1 (CH), 130.9 (CH), 130.73 (2CH), 130.65 (CH), 130.34 (CH), 130.25 (Cq), 129.7 (Cq), 129.52 (2CH), 129.47 (Cq), 129.4 (Cq), 129.3 (CH), 129.2 (CH), 128.9 (Cq), 128.1 (Cq), 127.8 (CH), 127.4 (CH), 127.3 (CH), 127.2 (CH), 127.1 (CH), 126.6 (CH), 123.6 (CH), 123.5 (CH), 123.4 (Cq), 122.9 (Cq), 114.2 (2CH), 108.9 (CH), 108.7 (CH), 54.59 (OCH_3), 54.57 (OCH_3) (one Cq signal was not observed due to overlap).

IR (ATR): $\tilde{\nu}$ = 3053, 2995, 2927, 2832, 1607, 1556, 1509, 1476, 1461, 1439, 1424, 1368, 1290, 1259, 1243, 1368, 1290, 1259, 1243, 1105, 1057, 1033, 1001, 971, 951, 901, 885, 869, 827, 800, 774, 750, 689, 644, 624, 590, 559, 523, 505, 478, 463, 445, 419 cm^{-1}

HRMS-ESI (m/z) calculated for $C_{44}H_{28}Br_2NaO_2S_3^+$ $[M+Na]^+$: 864.9510; found, 864.9493.

Melting point: 131–132 °C

$[\alpha]_{23}^{D}$: -35° (c = 0.04 in DCM) for 90% *ee*

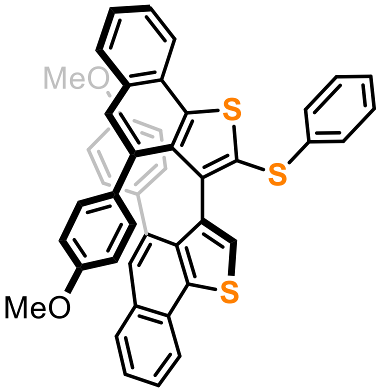

11a: Under a nitrogen atmosphere, a mixture of (+)-**1a** (12.2 mg, 0.02 mmol, 1.0 equiv.), $PhB(OH)_2$ (9.8 mg, 0.08 mmol, 4.0 equiv.), $Pd(dppf)Cl_2$ (1.5 mg, 2 µmol, 10 mol%), KF (4.6 mg, 0.08 mmol, 4:0 equiv.) in toluene (0.5 mL) and methanol (0.5 mL) was stirred at 110 °C overnight using microwave reactor. After cooling down to room temperature, DCM (10 mL) was added, and the organic phase was washed with water (2 × 5 mL). The combined organic phase was dried over Na_2SO_4, filtered, and the solvent removed under reduced pressure. The residue obtained was purified by HPLC (acetonitrile:H_2O 90:10→100:0 over 10 min, flow rate 1 mL/min at 22 °C) to deliver compound **11a** as a white solid in 45% yield (6.2 mg, 9 µmol) with 91% *ee*.

^{1}H-NMR (500 MHz, $CDCl_3$): δ = 8.04 – 8.01 (m, 1H), 7.93 – 7.88 (m, 1H), 7.83 – 7.80 (m, 1H), 7.79 – 7.76 (m, 1H), 7.54 – 7.50 (m, 3H), 7.50 – 7.45 (m, 3H), 7.41 – 7.36 (m, 2H), 7.35 – 7.31 (m, 2H), 7.30 (s, 1H), 7.21 (s, 1H), 7.06 – 6.87 (m, 2H), 6.67 – 6.39 (m, 2H), 6.33 – 6.09 (m, 2H), 5.91 – 5.61 (m, 2H), 3.09 (s, OCH_3), 3.07 (s, OCH_3).

^{13}C{^{1}H}-NMR (126 MHz, $CDCl_3$) δ = 157.3 (Cq), 157.1 (Cq), 139.5 (Cq), 138.8 (Cq), 138.6 (Cq), 137.0 (Cq), 136.9 (Cq), 135.8 (Cq), 135.0 (Cq), 133.7 (Cq), 131.0 (Cq), 130.9 (Cq), 130.6

(Cq), 130.5 (2CH), 130.4 (Cq), 130.3 (Cq), 129.8 (2CH), 129.6 (2CH), 128.5 (2CH),128.34 (CH), 128.32 (CH), 127.9 (Cq), 127.6 (CH), 127.1 (Cq), 126.8 (CH), 126.21 (CH), 126.17 (CH), 126.1 (CH), 126.0 (CH), 125.8 (CH), 125.6 (CH), 123.15 (CH), 123.08 (CH), 112.7 (2CH), 110.0 (CH), 109.7 (CH), 54.68 (OCH_3), 54.65 (OCH_3) (one Cq signal was not observed due to overlap).

IR (ATR): $\tilde{\nu}$ = 2954, 2925, 2853, 1711, 1609, 1578, 1510, 1494, 1462, 1433, 1378, 1317, 1294, 1242, 1171, 1107, 1086, 1031, 998, 967, 944, 920, 885, 862, 824, 808, 779, 771, 743, 689, 674, 635, 626, 597, 571, 550, 519, 455, 425, 403 cm^{-1}

HRMS-ESI (m/z) calculated for $C_{44}H_{30}NaO_2S_3^+$ $[M+Na]^+$: 709.1300; found, 709.1314.

$[\boldsymbol{\alpha}]_{\mathbf{25}}^{\boldsymbol{D}}$: –88° (*c* = 0.034 in DCM) for 91% *ee*

Compound **3a**

^{1}H-NMR (300 MHz, CD_2Cl_2)

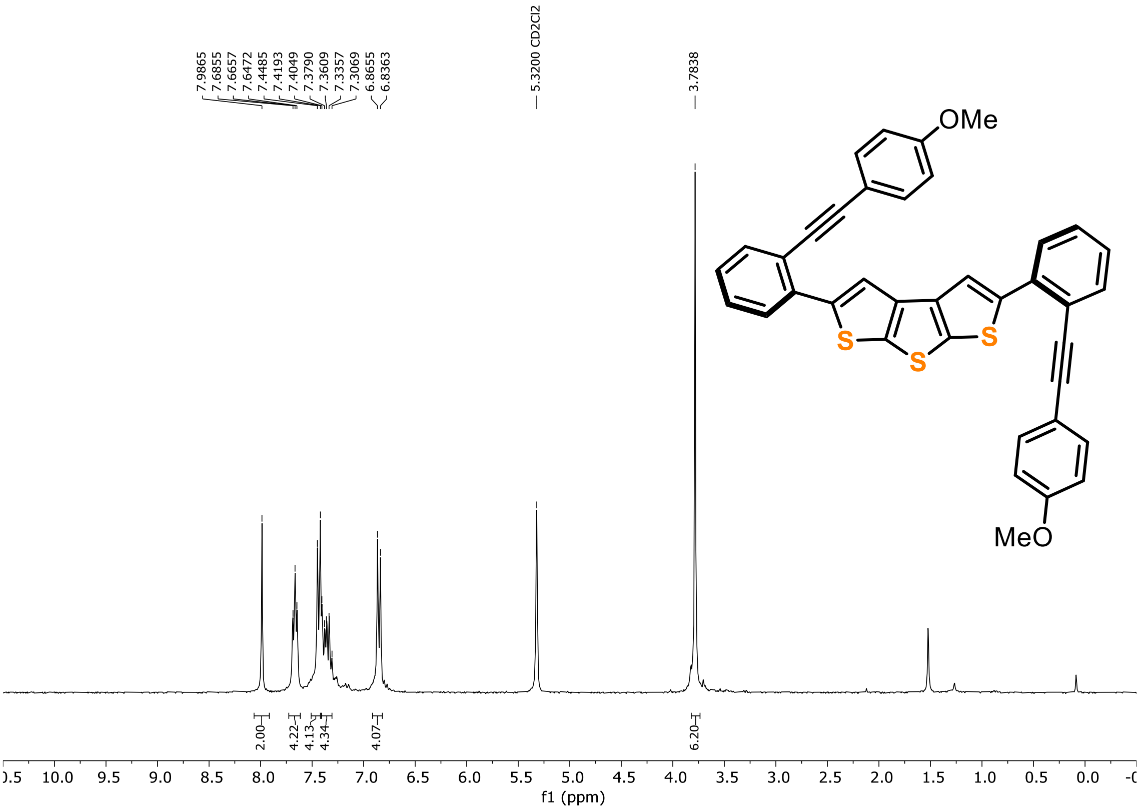

^{13}C{^{1}H}-NMR (101 MHz, CD_2Cl_2)

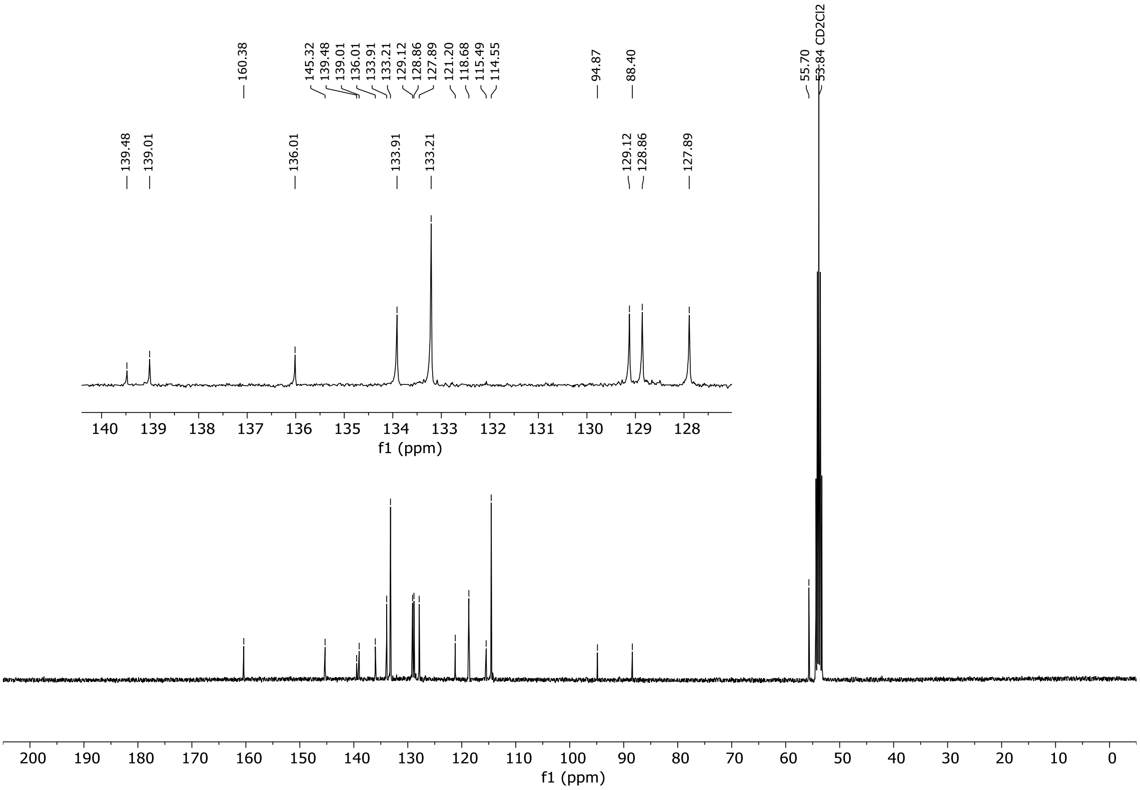

Compound **3b**

^{1}H-NMR (300 MHz, $CDCl_3$)

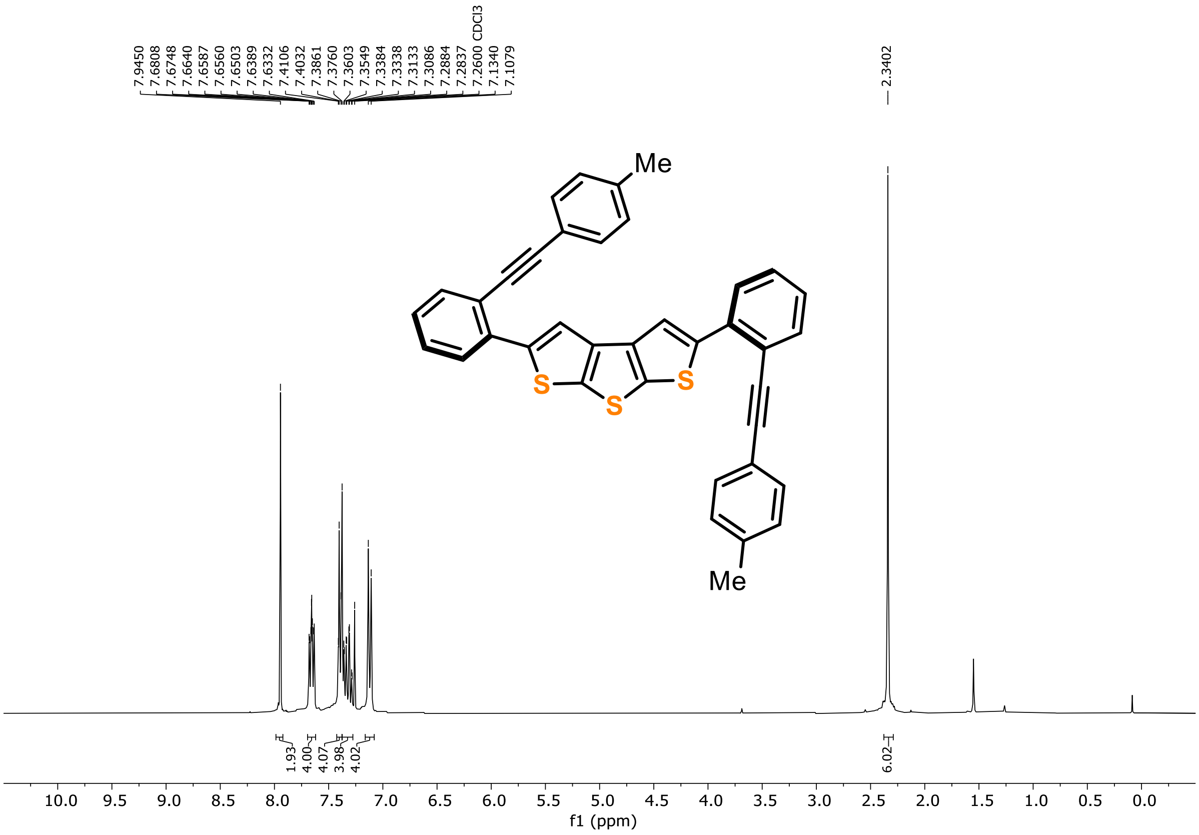

^{13}C{^{1}H}-NMR (101 MHz, $CDCl_3$)

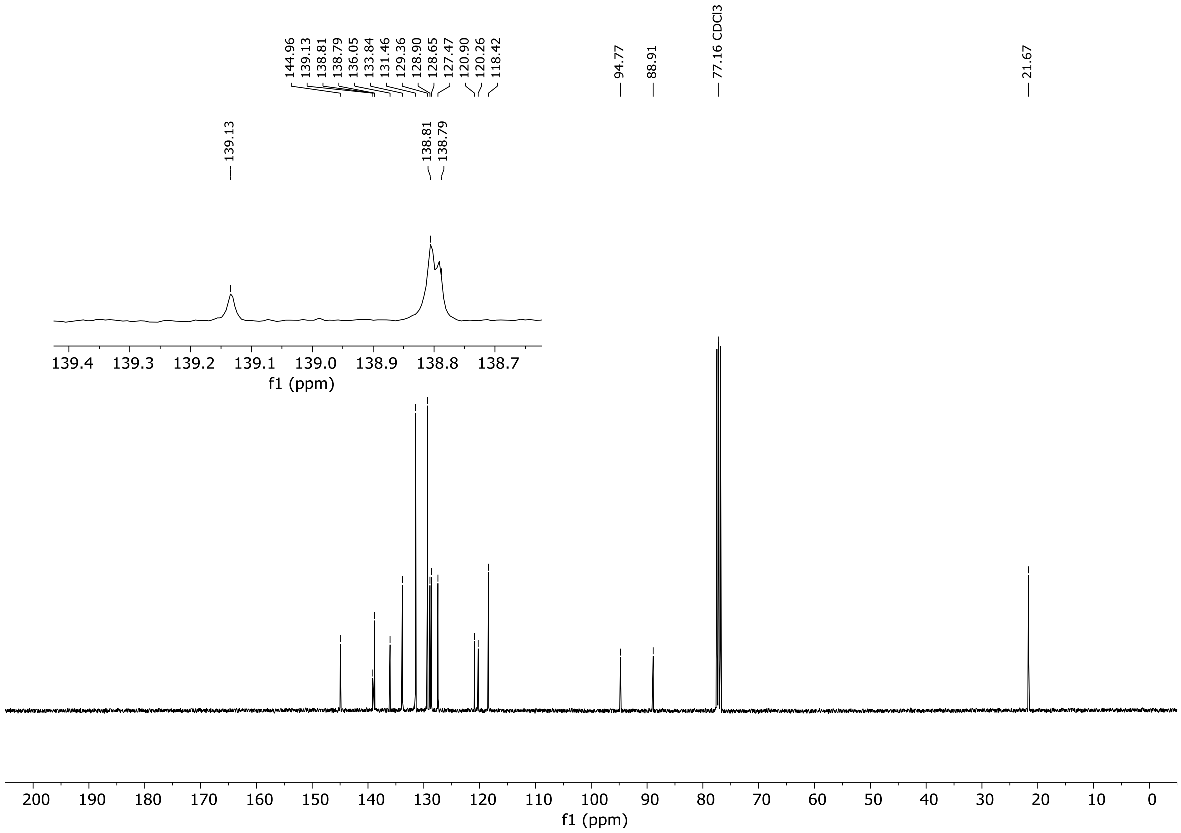

Compound **3c**

^{1}H-NMR (300 MHz, $CDCl_3$)

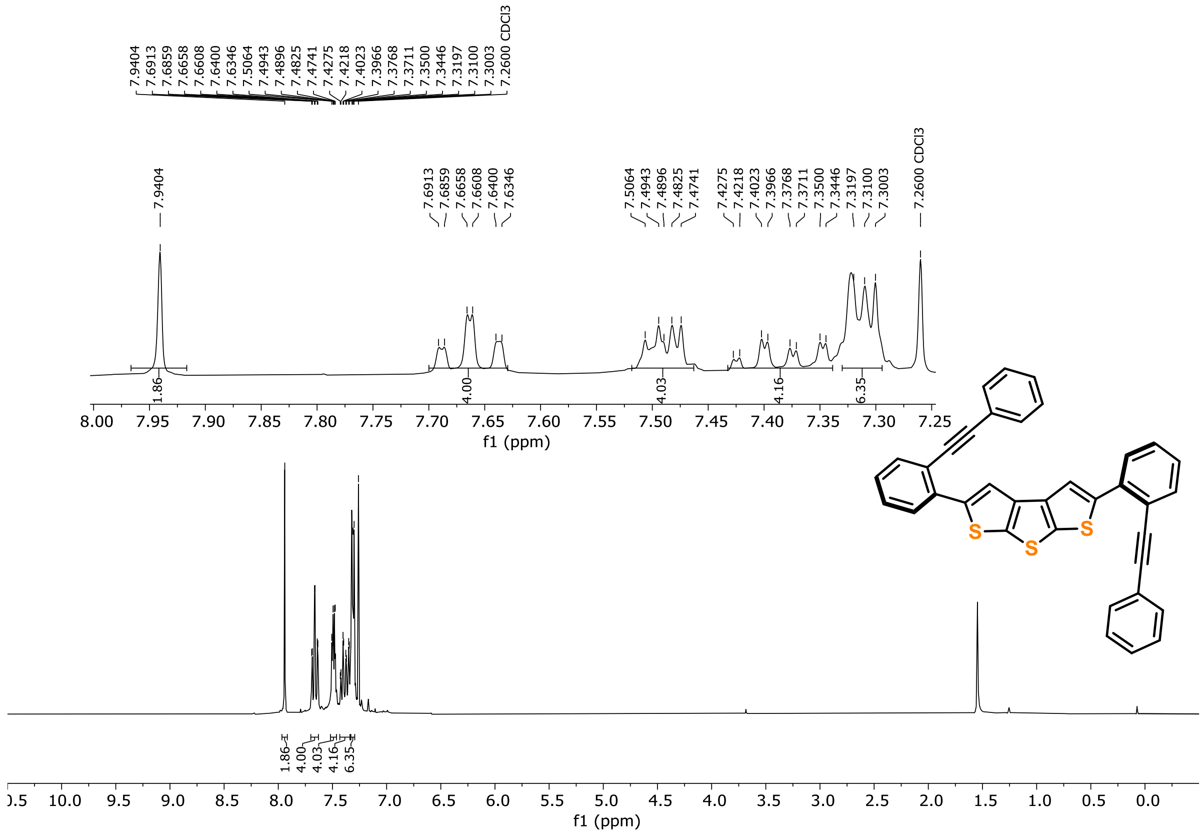

^{13}C{^{1}H}-NMR (101 MHz, $CDCl_3$)

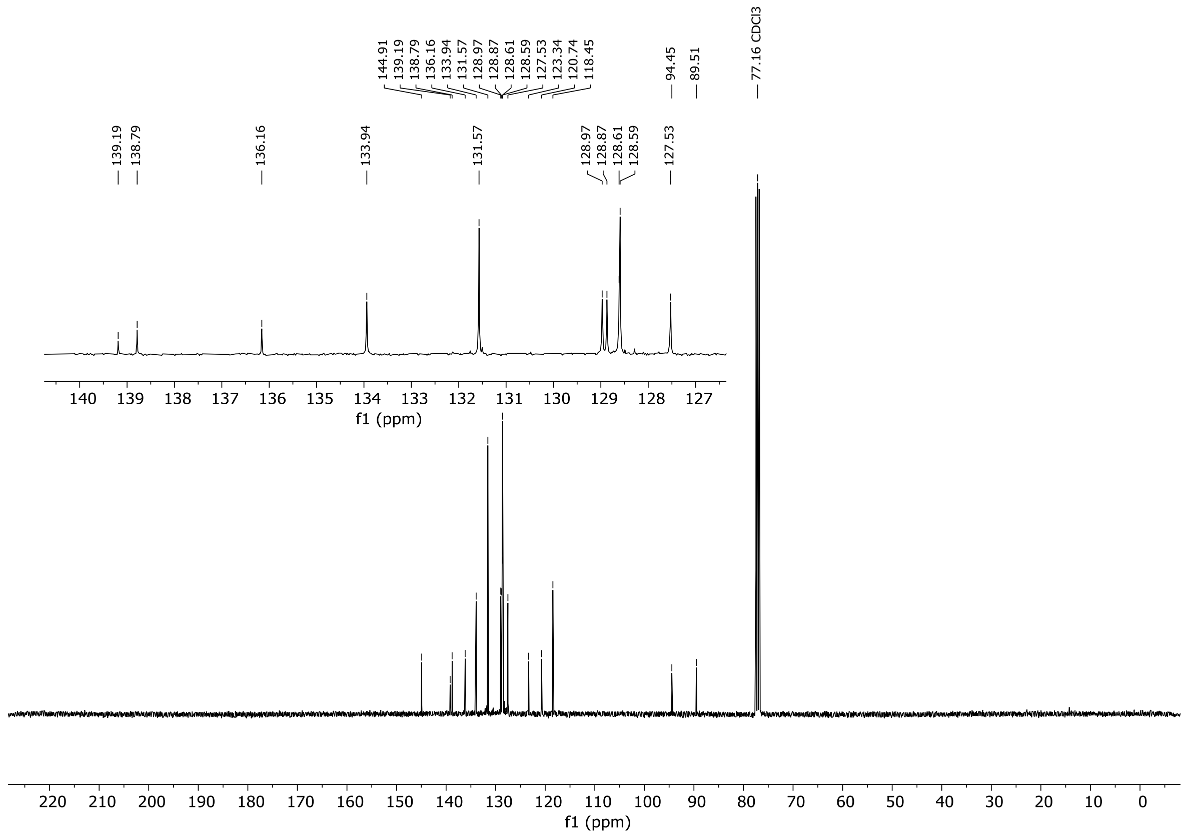

Compound **3d**

^{1}H-NMR (300 MHz, $CDCl_3$)

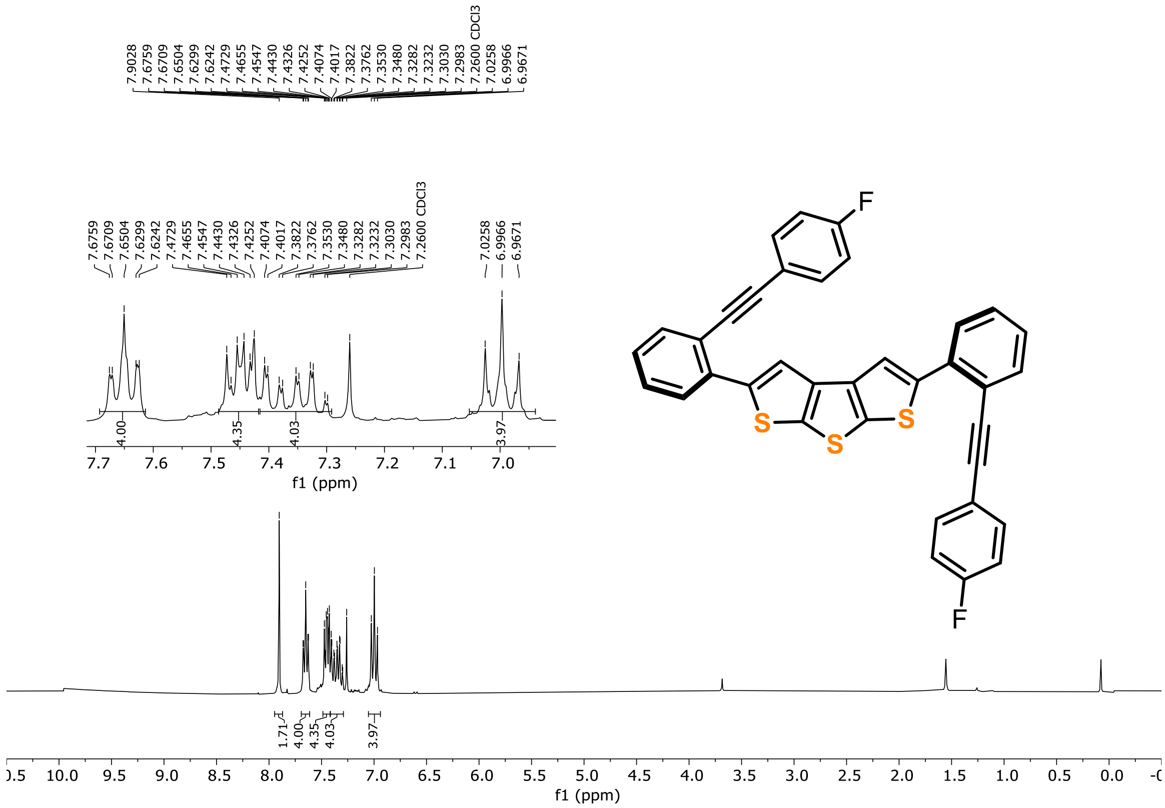

$^{13}C\{^{1}H\}$-NMR (101 MHz, $CDCl_3$)

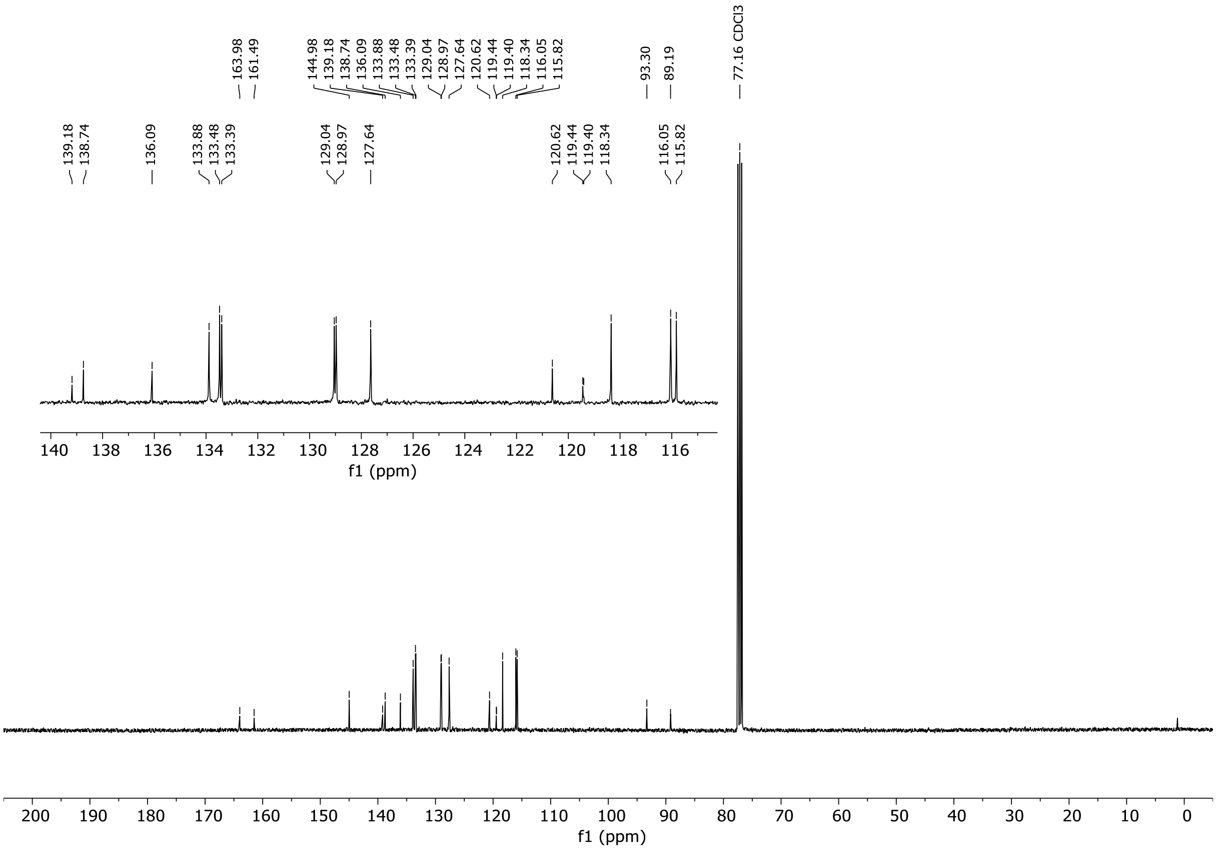

^{19}F-NMR (282 MHz, $CDCl_3$)

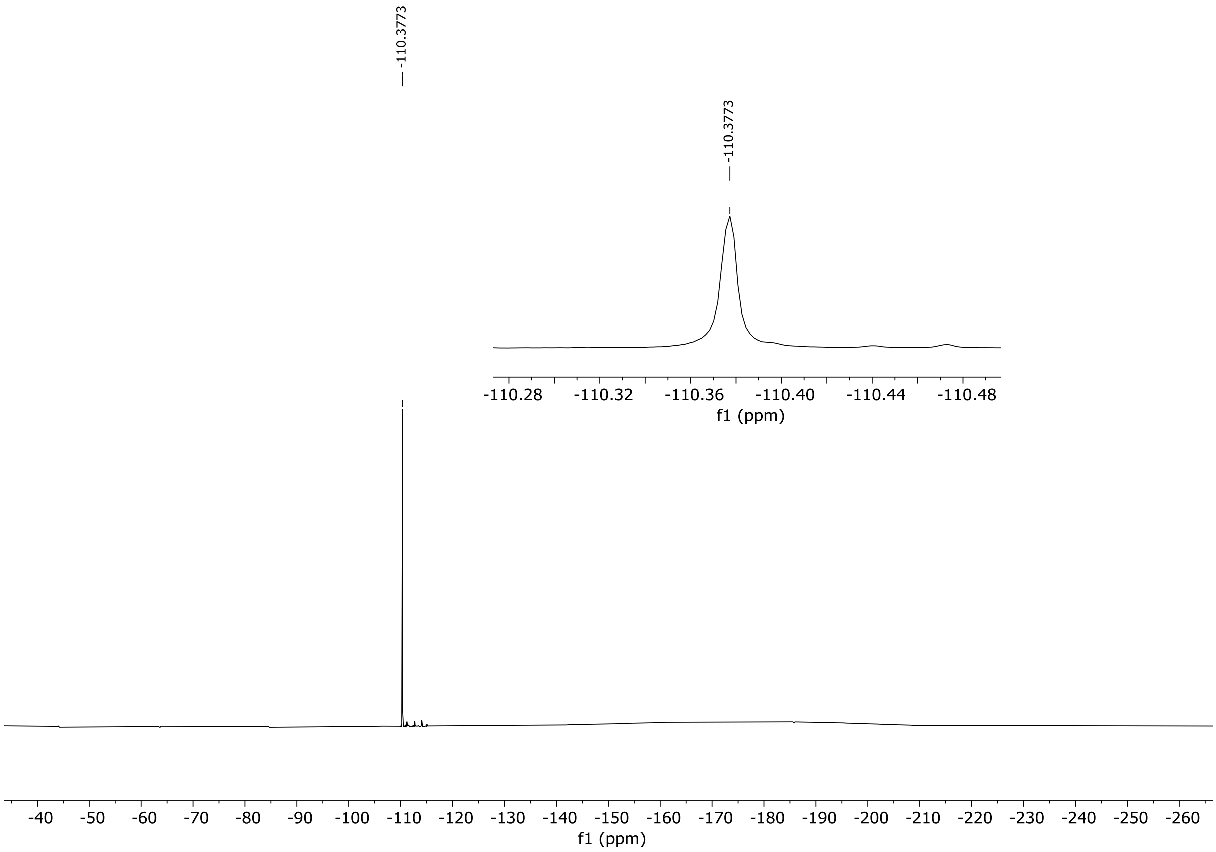

Compound **3e**

^{1}H-NMR (400 MHz, $CDCl_3$)

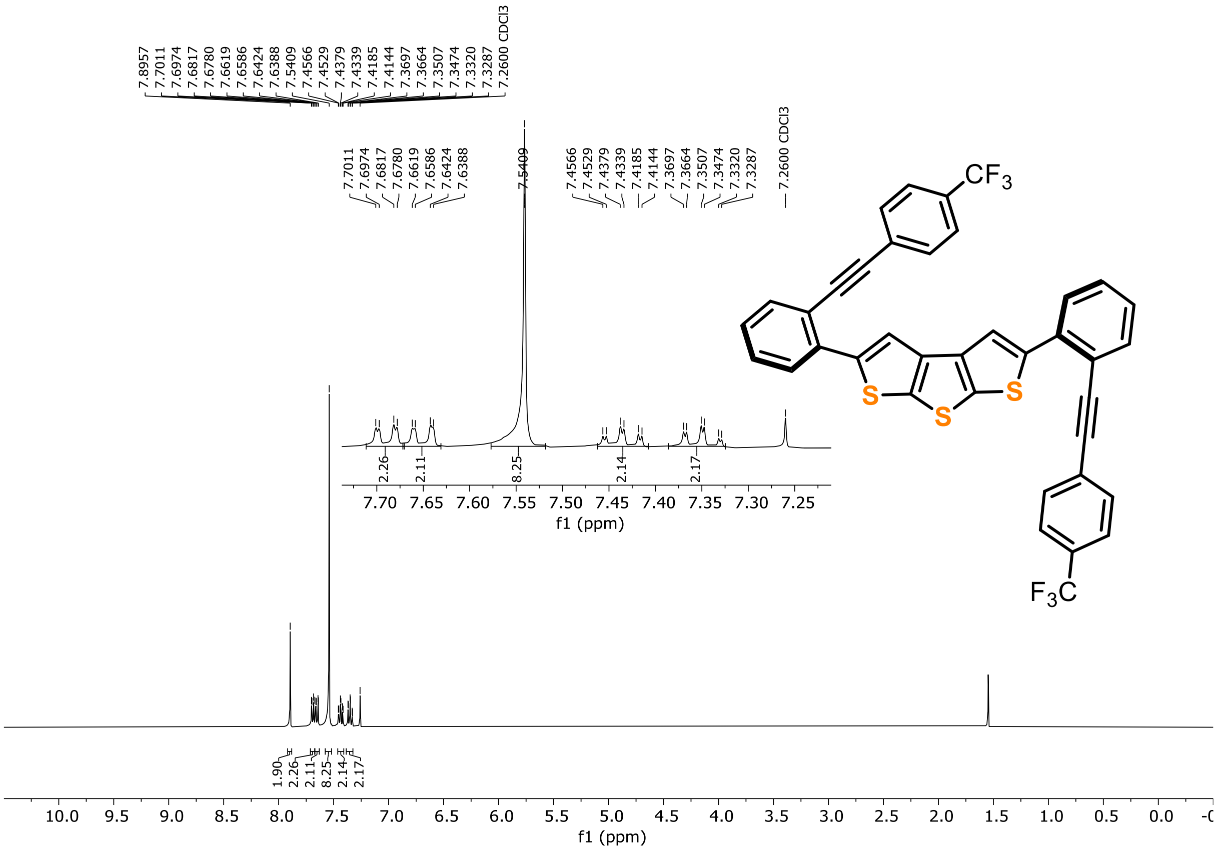

$^{13}C\{^1H\}$-NMR (101 MHz, $CDCl_3$)

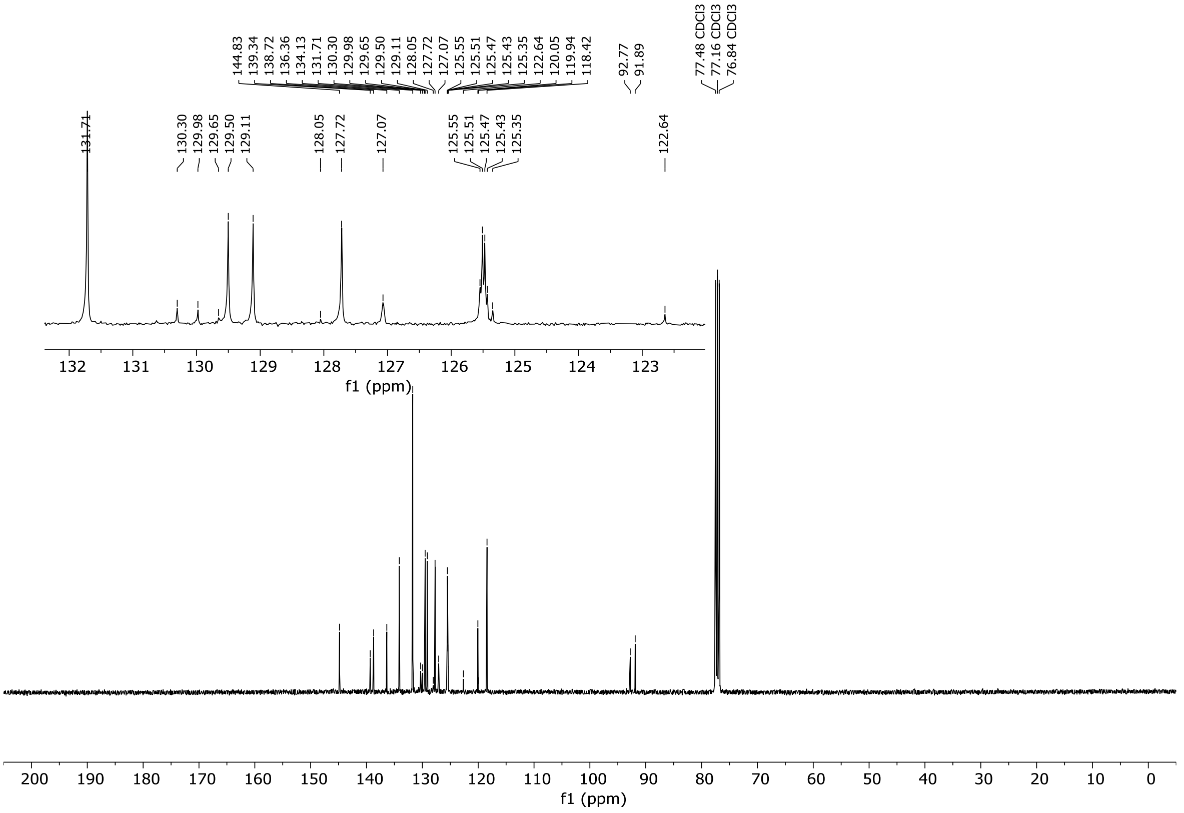

^{19}F-NMR (377 MHz, $CDCl_3$)

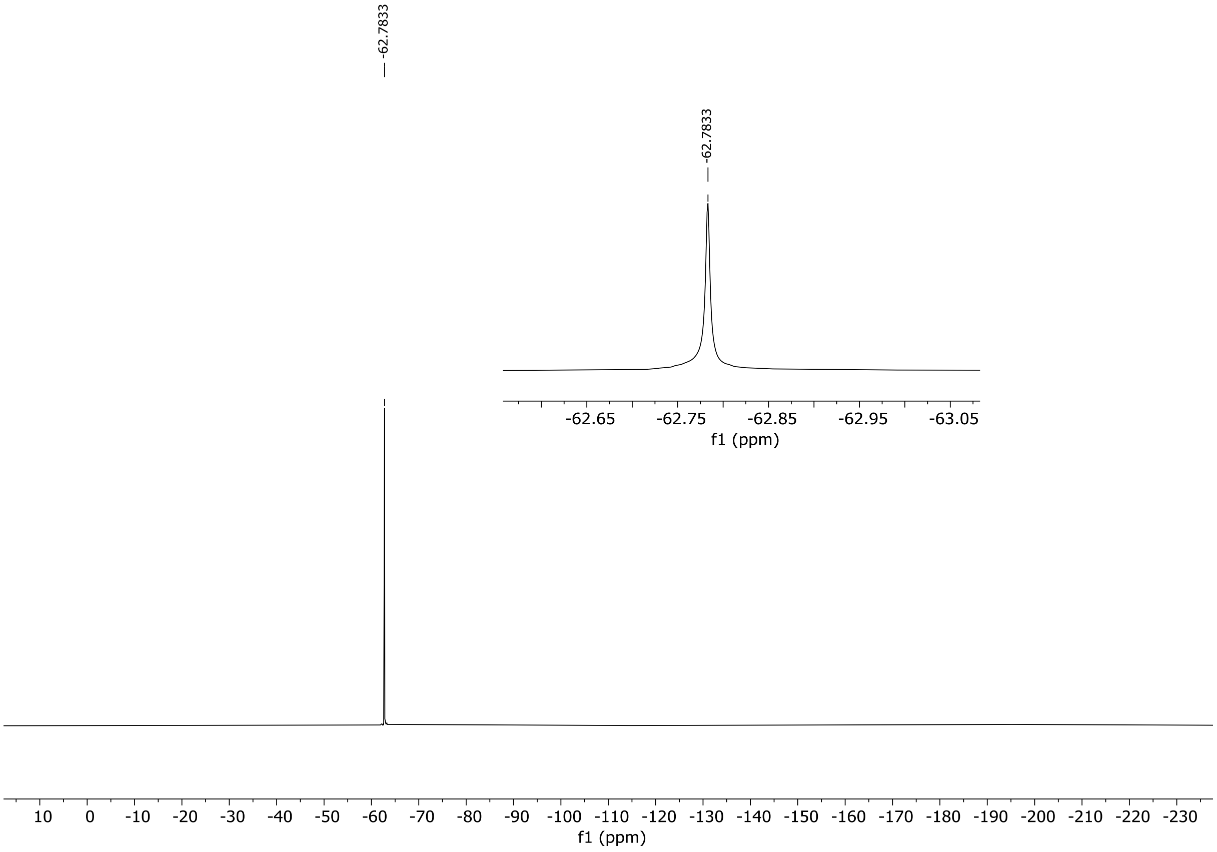

Compound **3f**

^{1}H-NMR (300 MHz, $CDCl_3$)

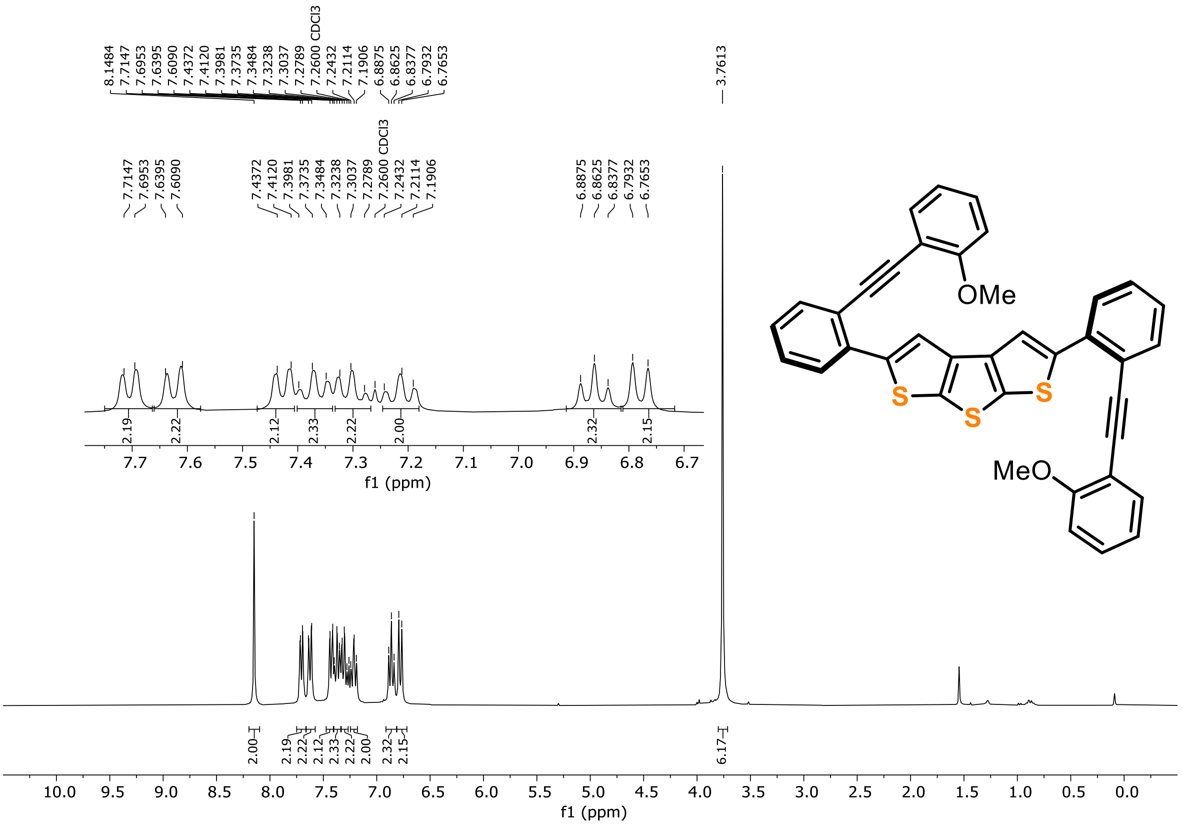

^{13}C{^{1}H}-NMR (101 MHz, $CDCl_3$)

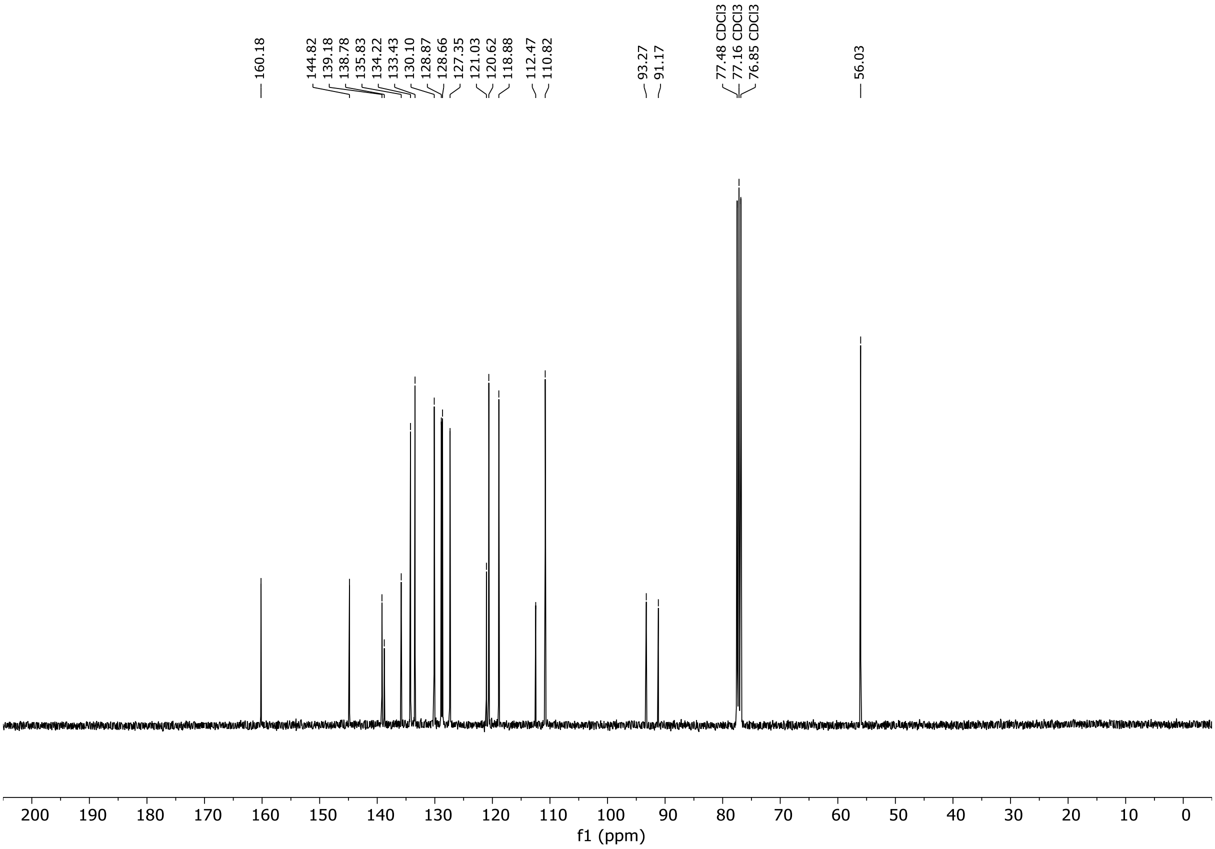

Compound **3g**

^{1}H-NMR (300 MHz, CD_2Cl_2)

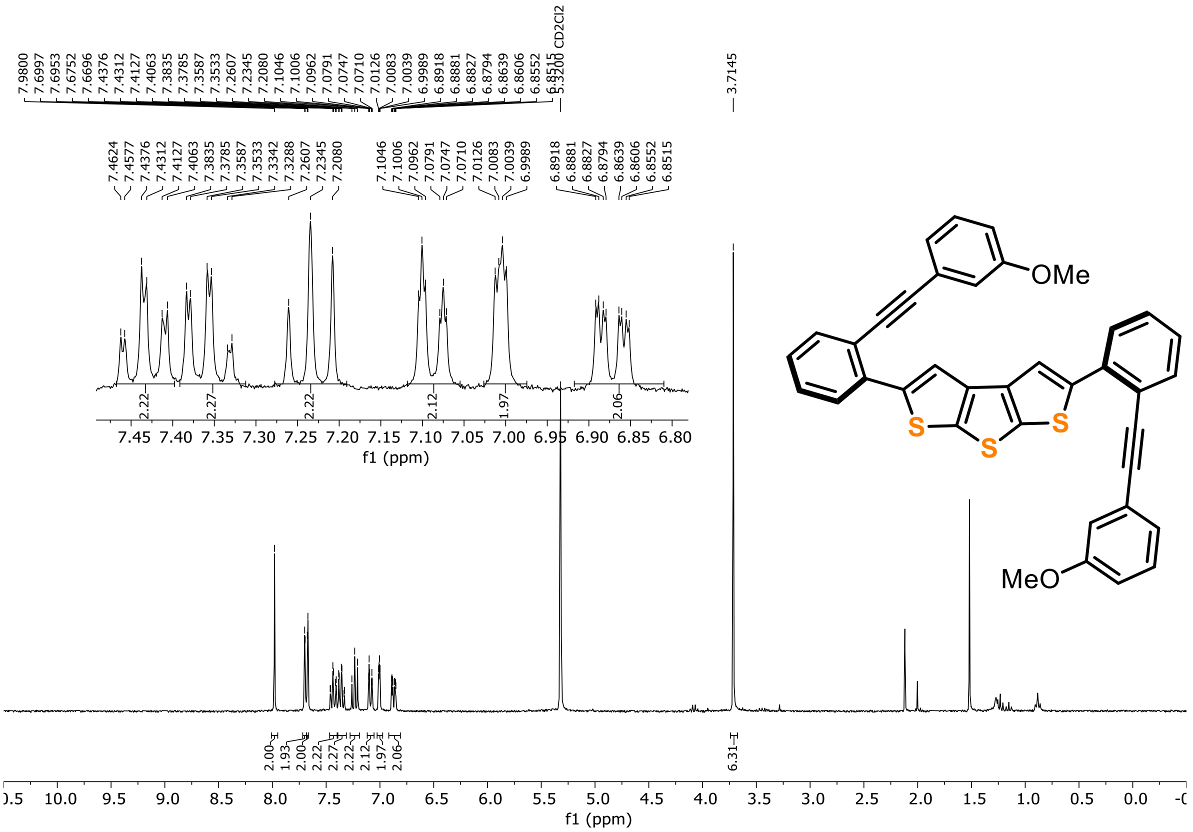

$^{13}C\{^1H\}$-NMR (101 MHz, CD_2Cl_2)

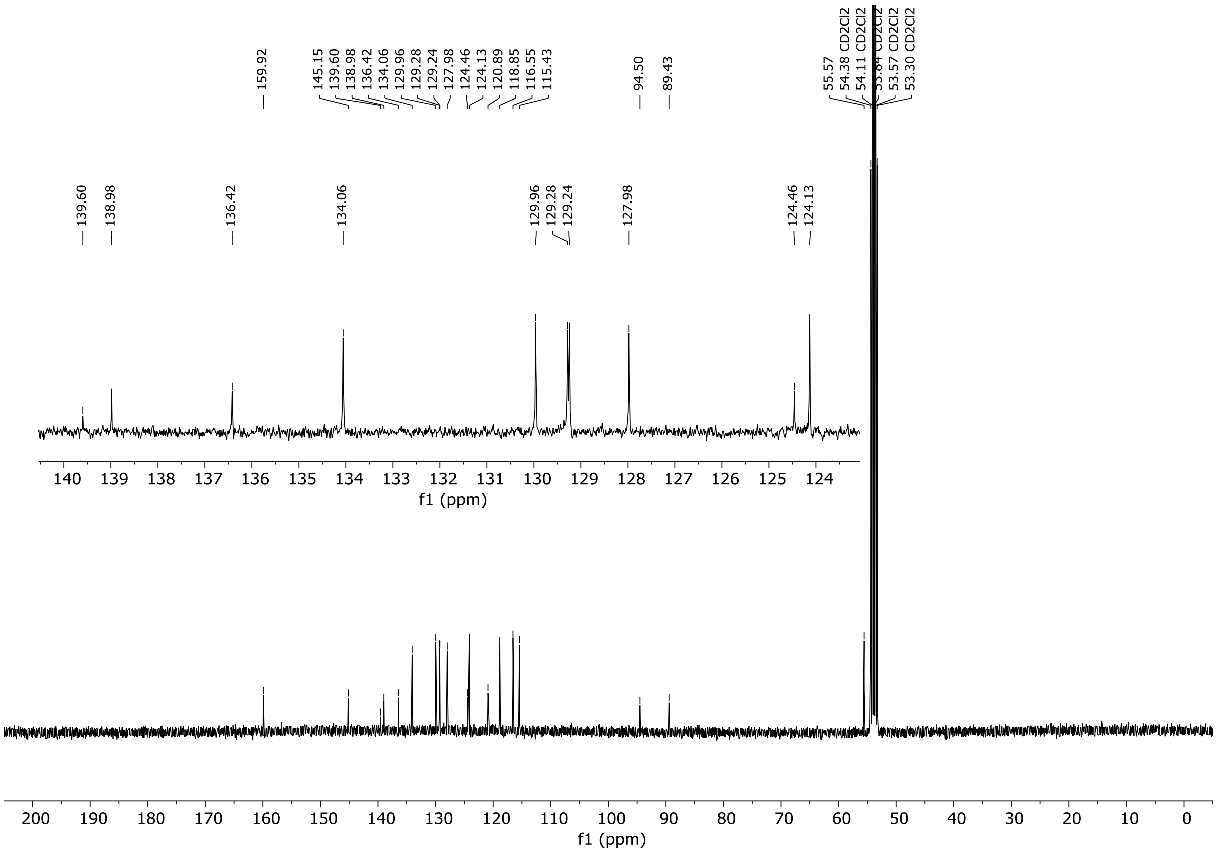

Compound **3h**

^{1}H-NMR (300 MHz, $CDCl_3$)

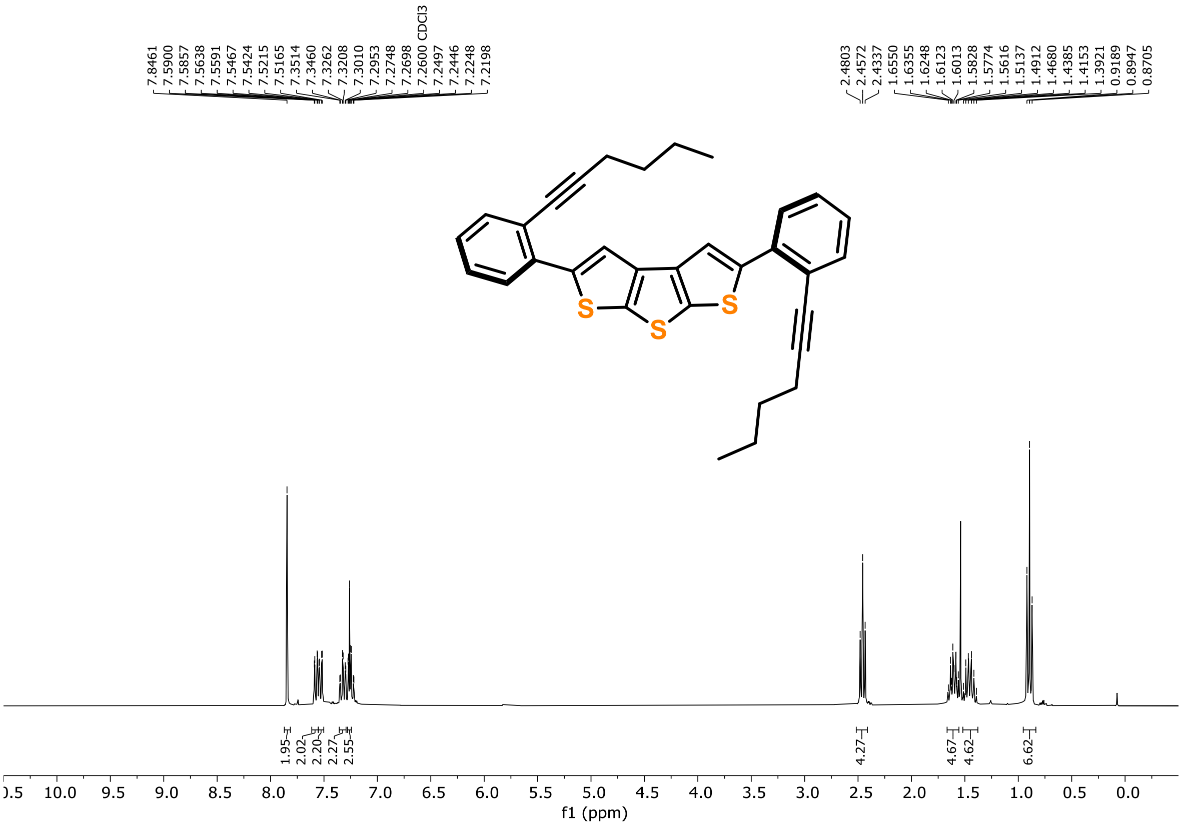

$^{13}C\{^1H\}$-NMR (101 MHz, $CDCl_3$)

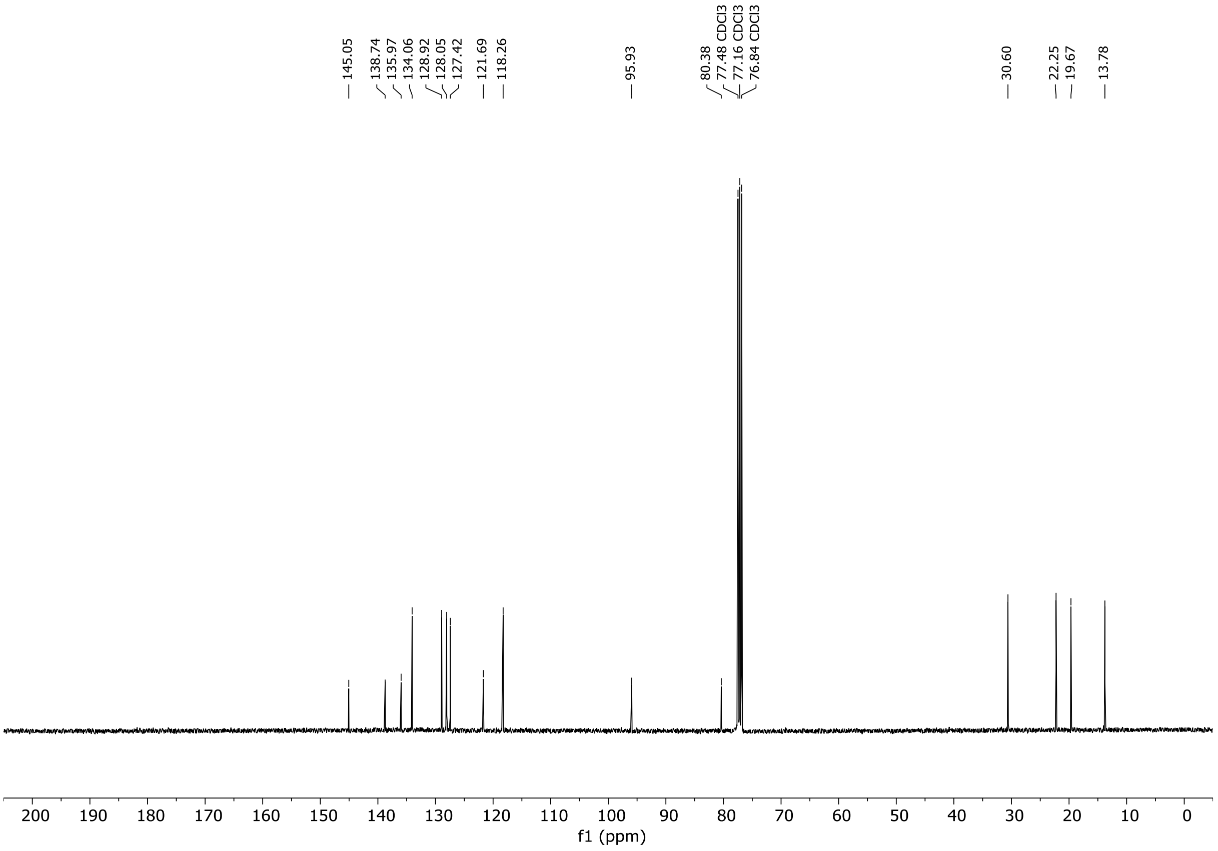

Compound **S2d**

^{1}H-NMR (300 MHz, CD_2Cl_2)

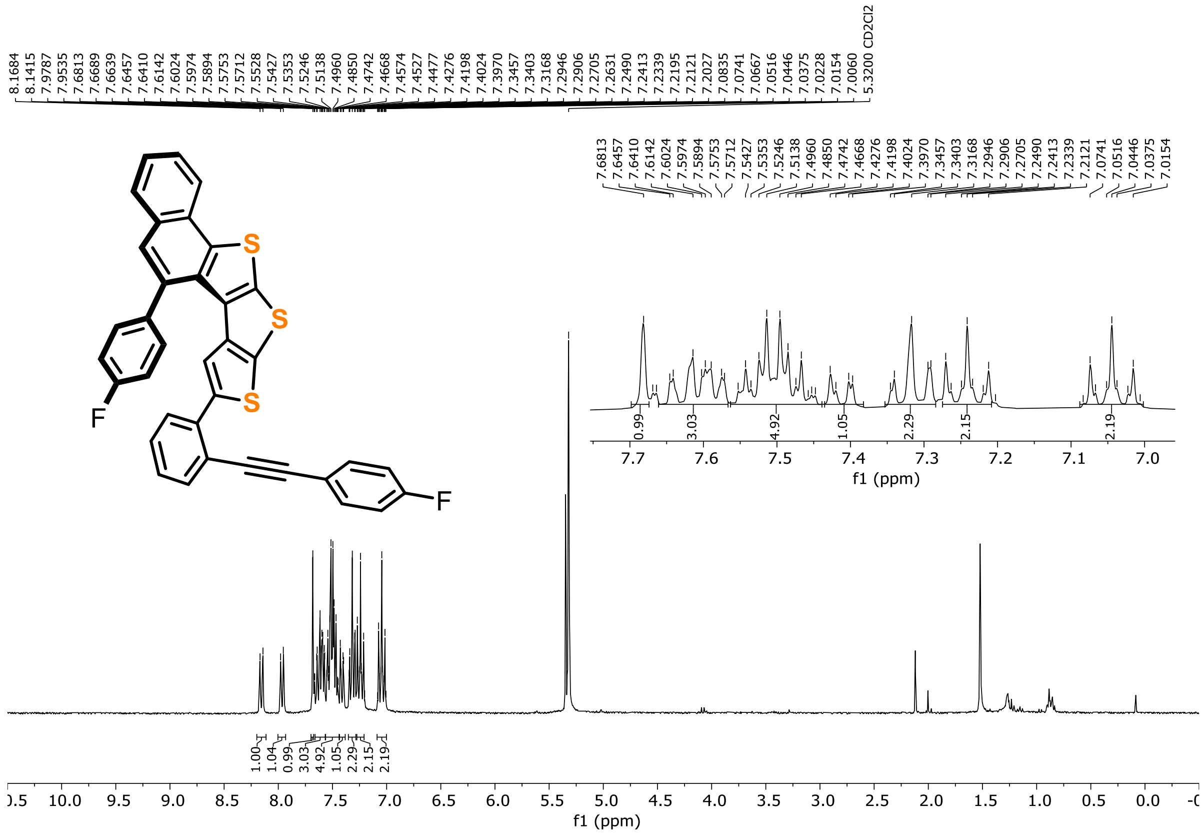

^{13}C{APT}-NMR (126 MHz, CD_2Cl_2)

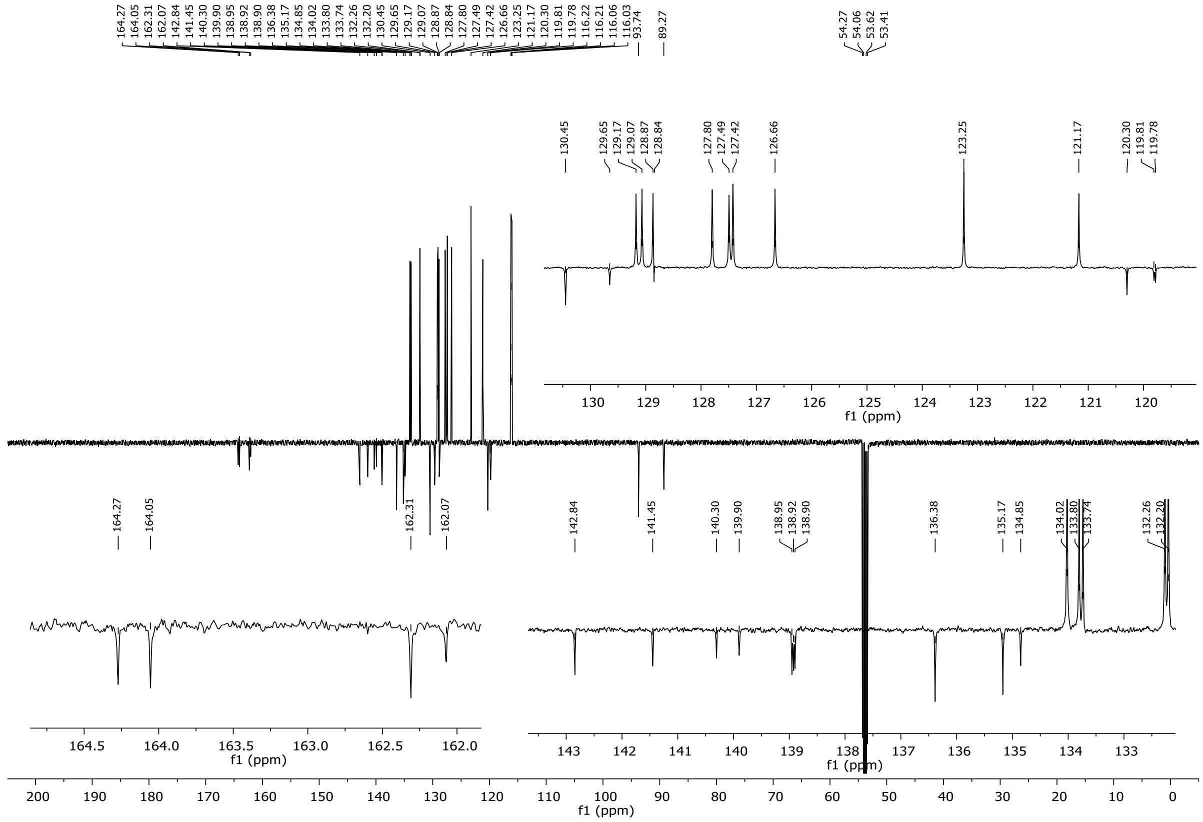

^{19}F-NMR (282 MHz, CD_2Cl_2)

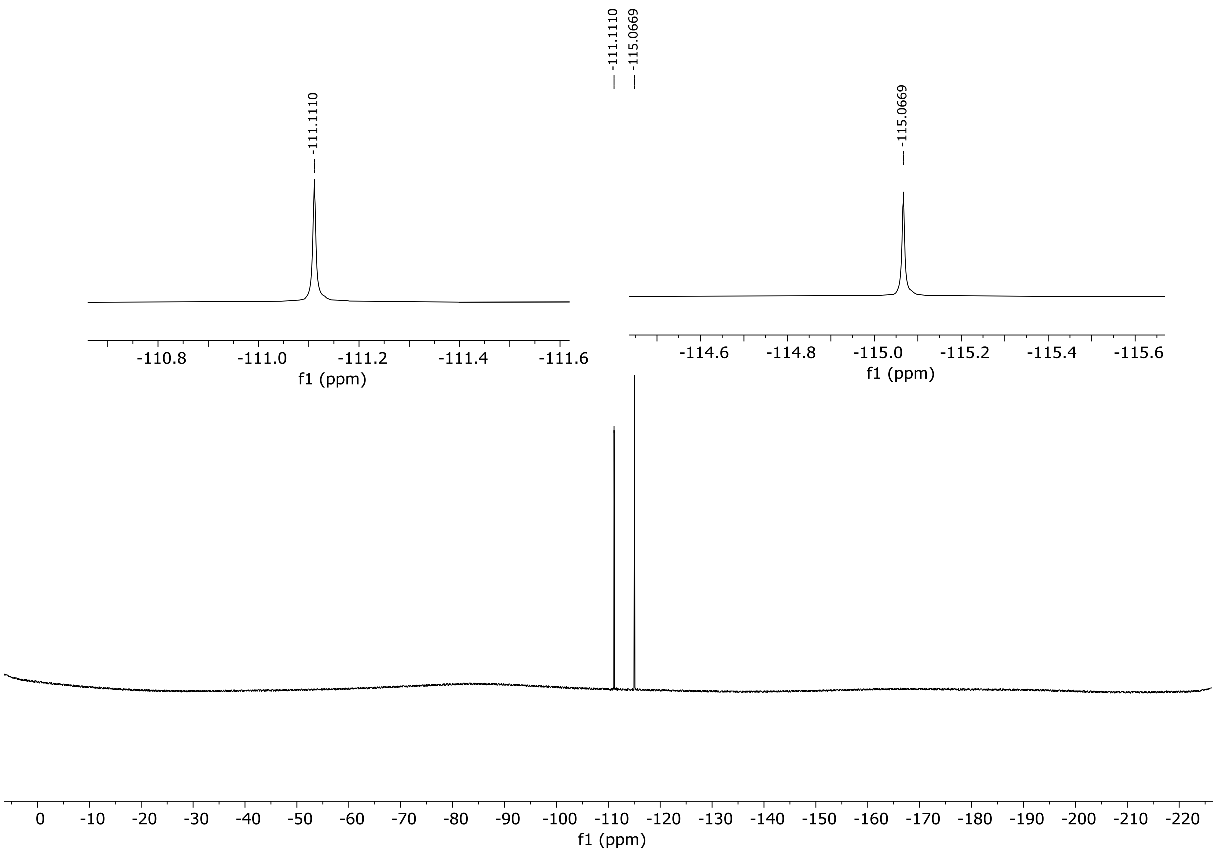

Compound **S2h**

^{1}H-NMR (300 MHz, CD_2Cl_2)

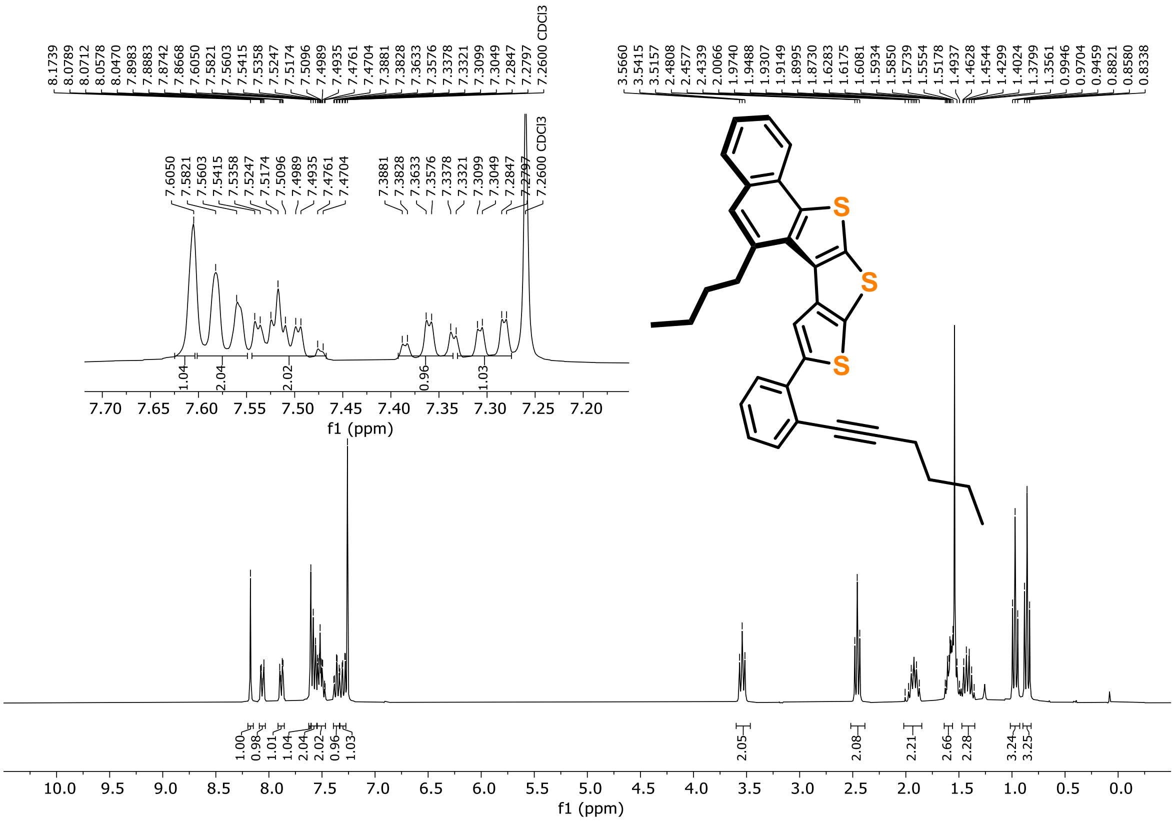

^{13}C{^{1}H}-NMR (101 MHz, $CDCl_3$)

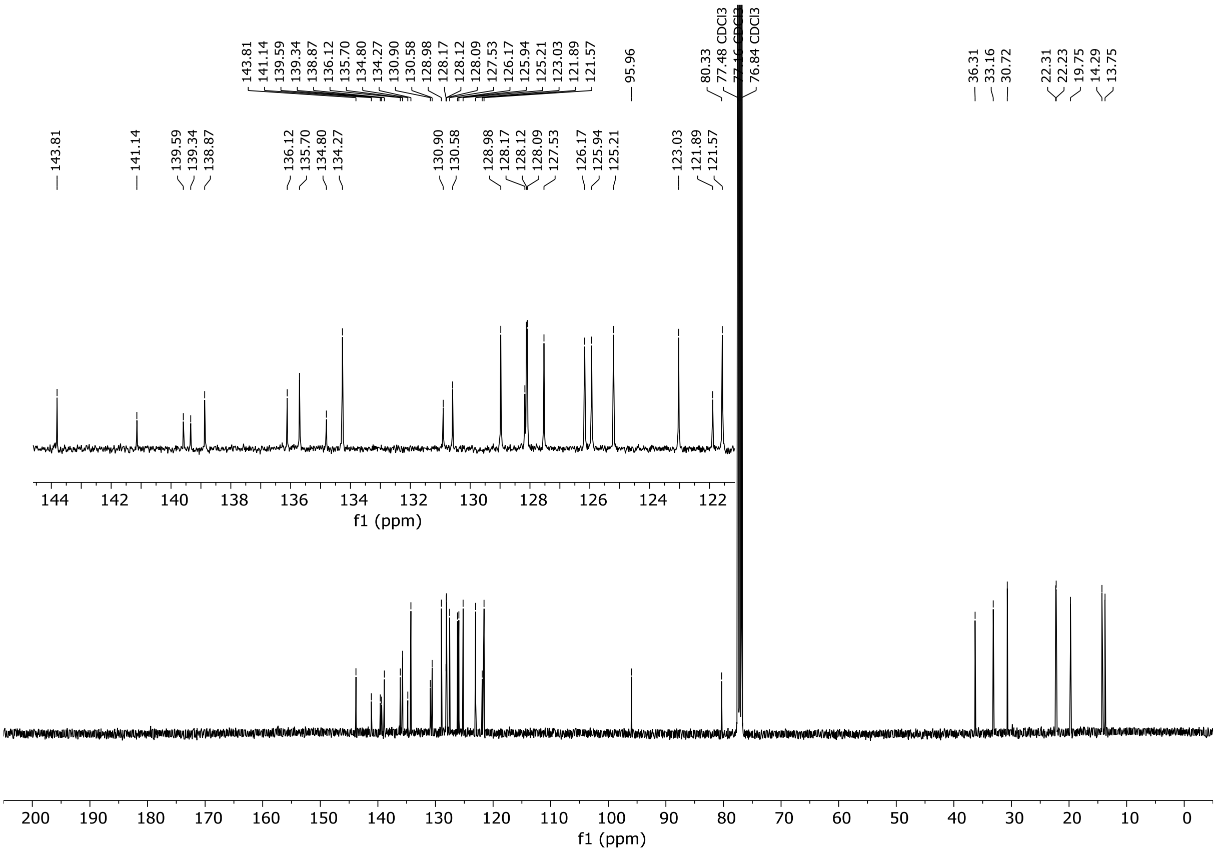

Compound **1a**

^{1}H-NMR (300 MHz, $CDCl_3$)

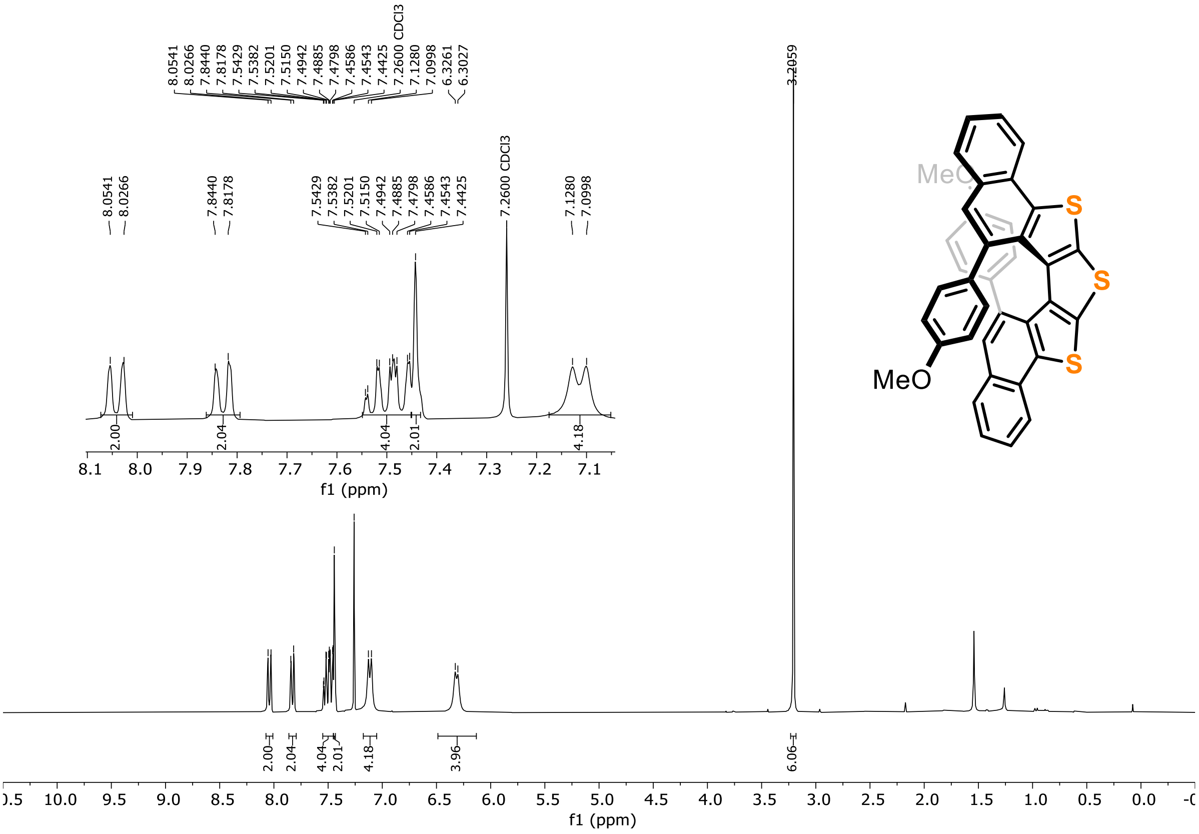

^{13}C{^{1}H}-NMR (101 MHz, $CDCl_3$)

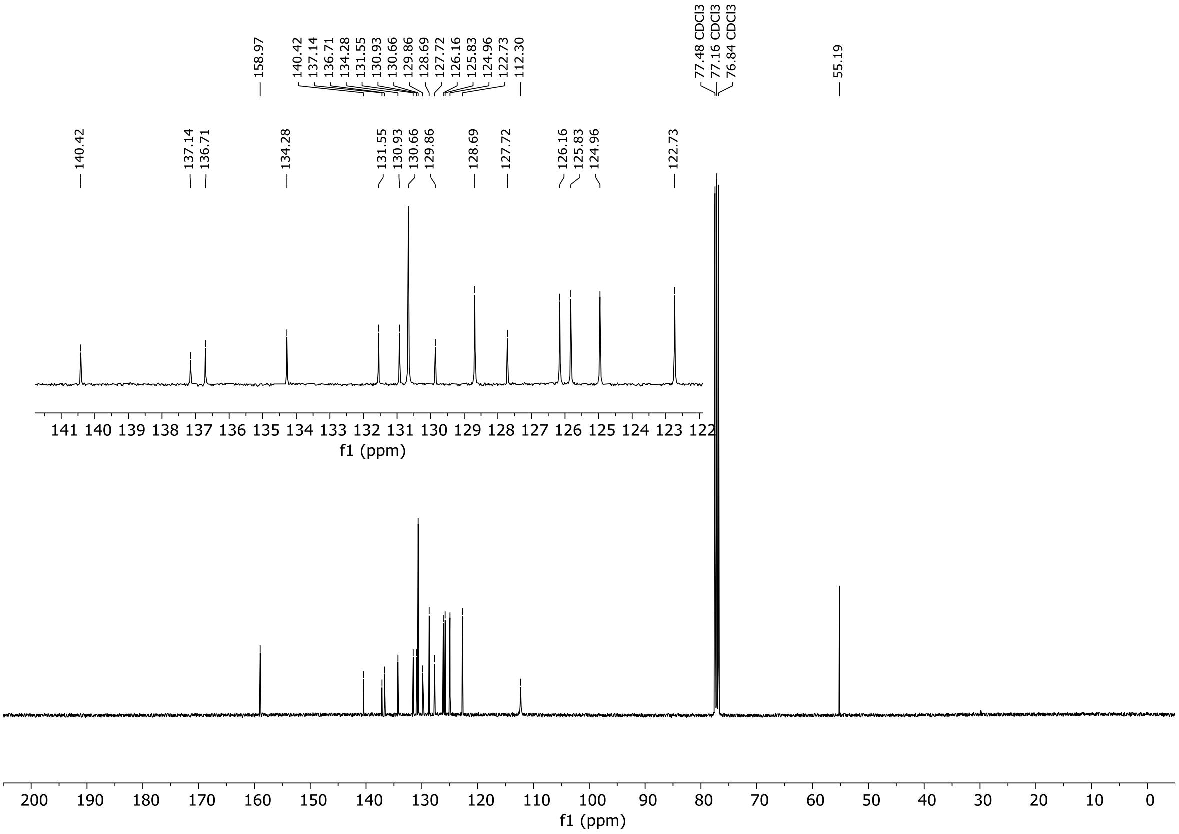

Compound **1b**

^{1}H-NMR (300 MHz, $CDCl_3$)

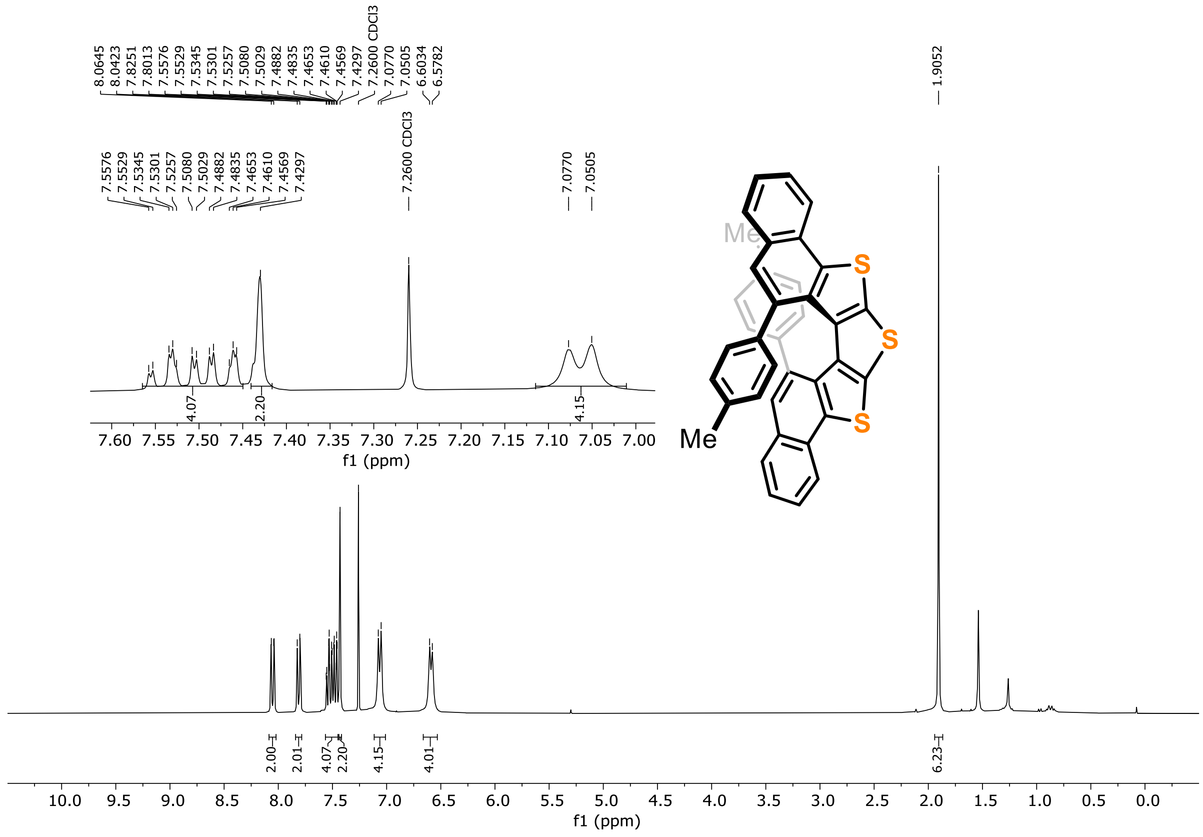

^{13}C{^{1}H}-NMR (101 MHz, $CDCl_3$)

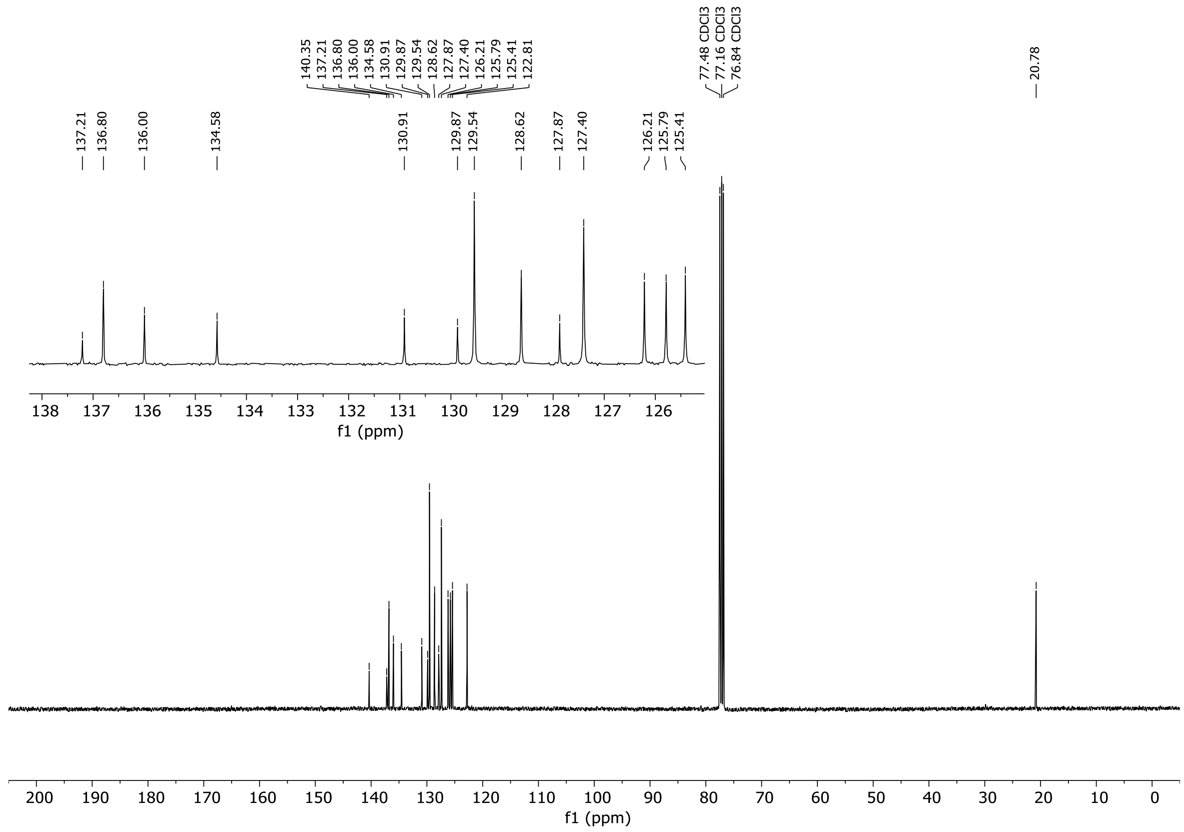

Compound **1c**

^{1}H-NMR (300 MHz, CD_2Cl_2)

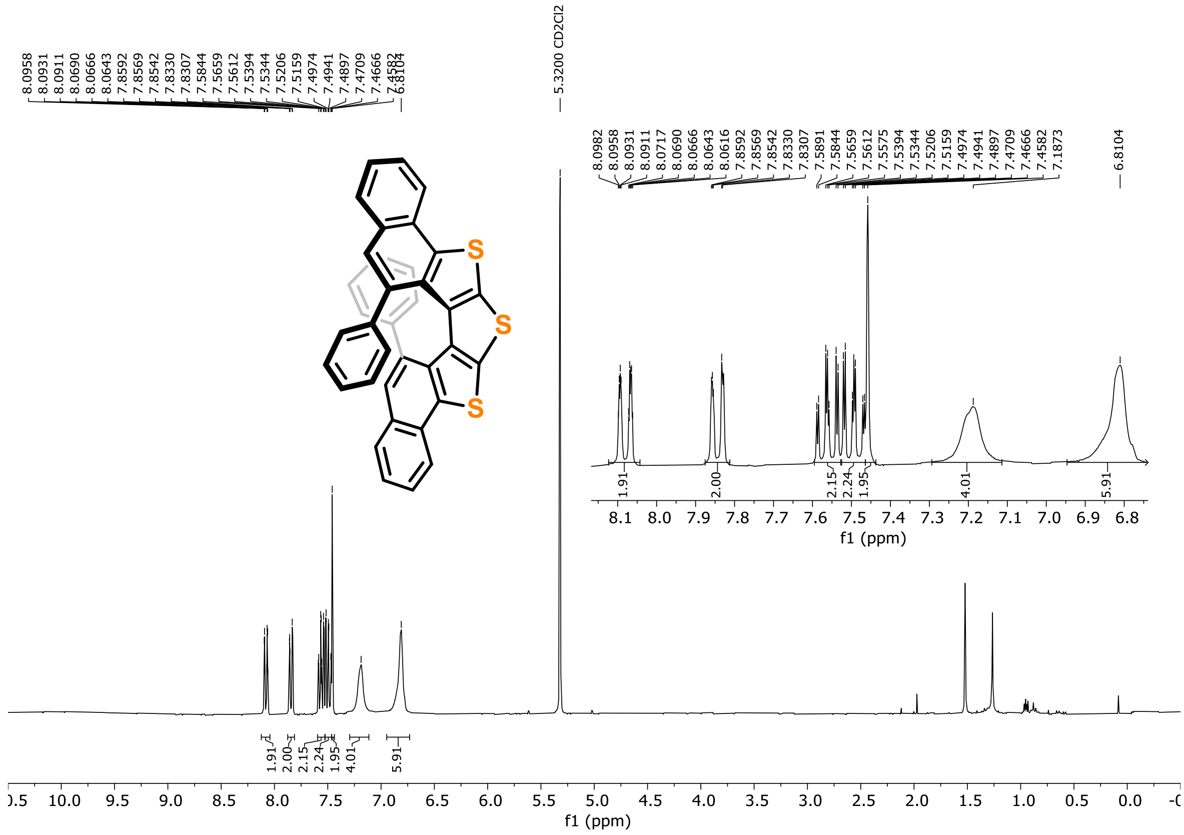

^{13}C{^{1}H}-NMR (101 MHz, CD_2Cl_2)

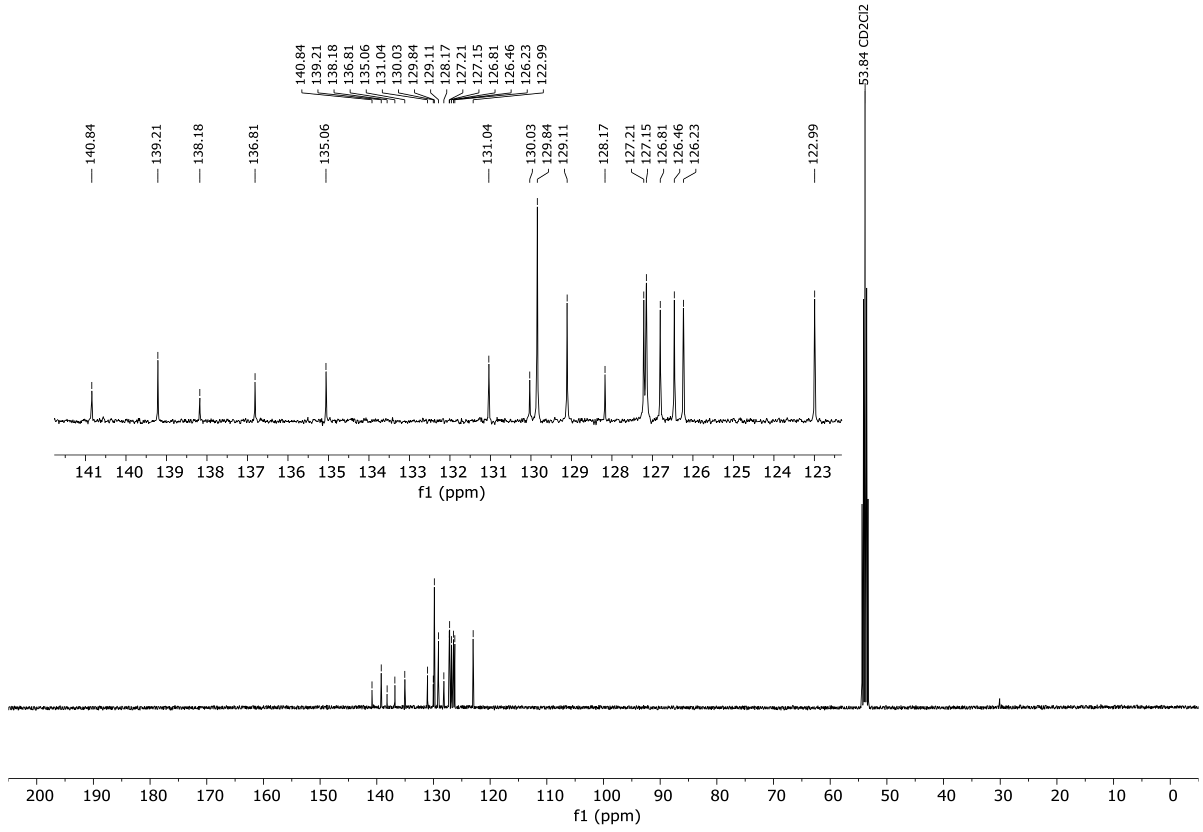

Compound **1d**

^{1}H-NMR (300 MHz, CD_2Cl_2)

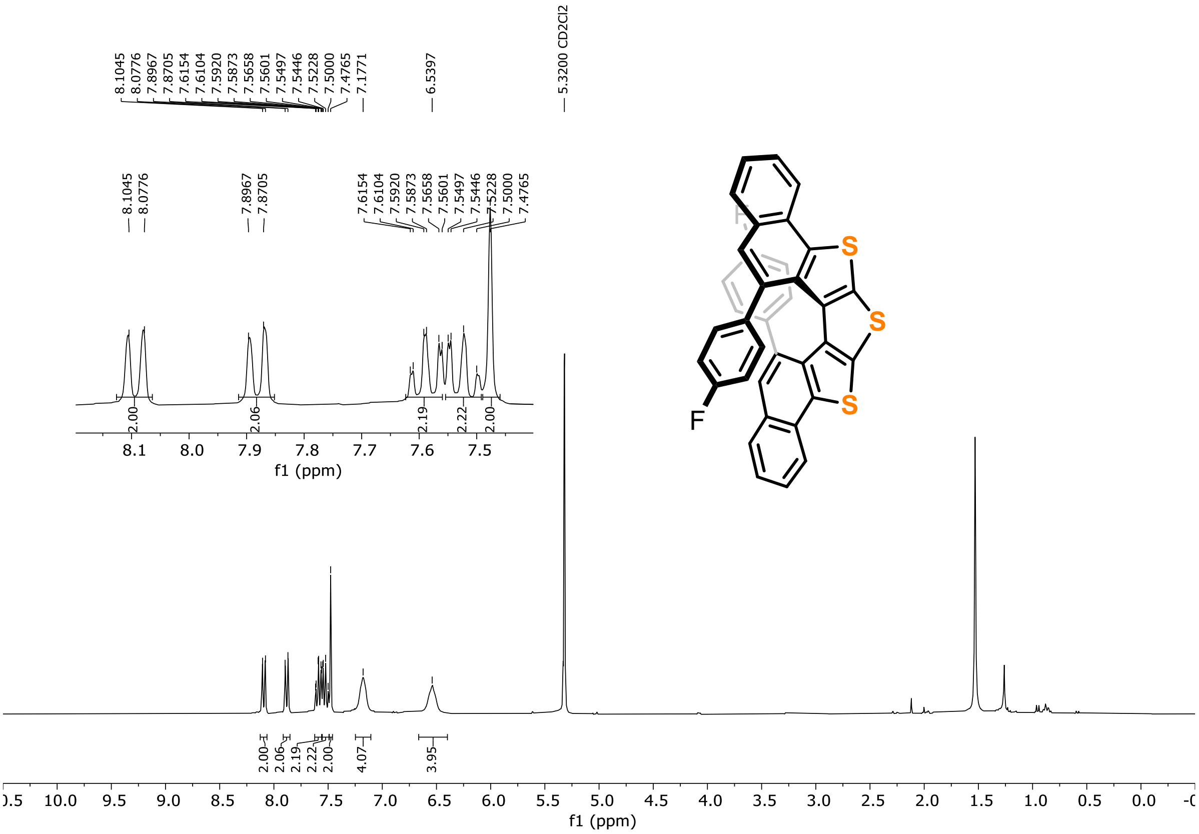

^{13}C{^{1}H}-NMR (101 MHz, CD_2Cl_2)

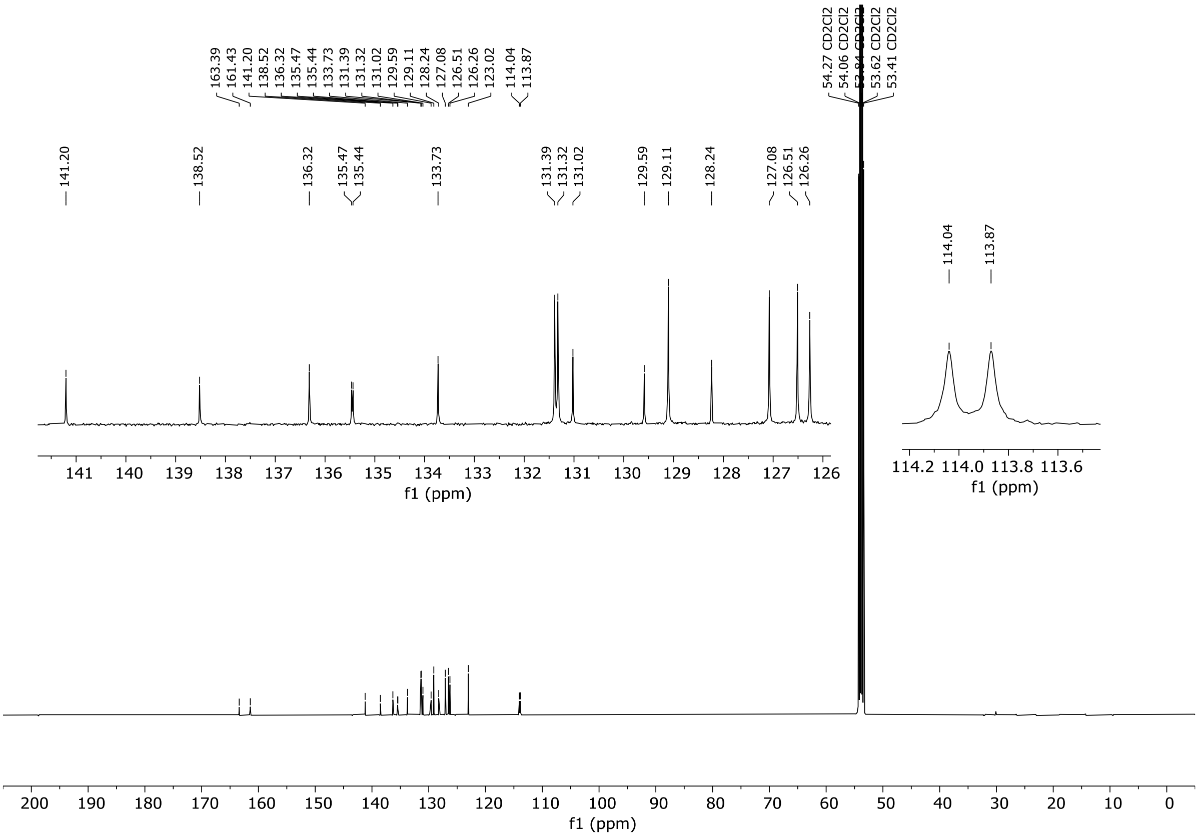

^{19}F-NMR (282 MHz, CD_2Cl_2)

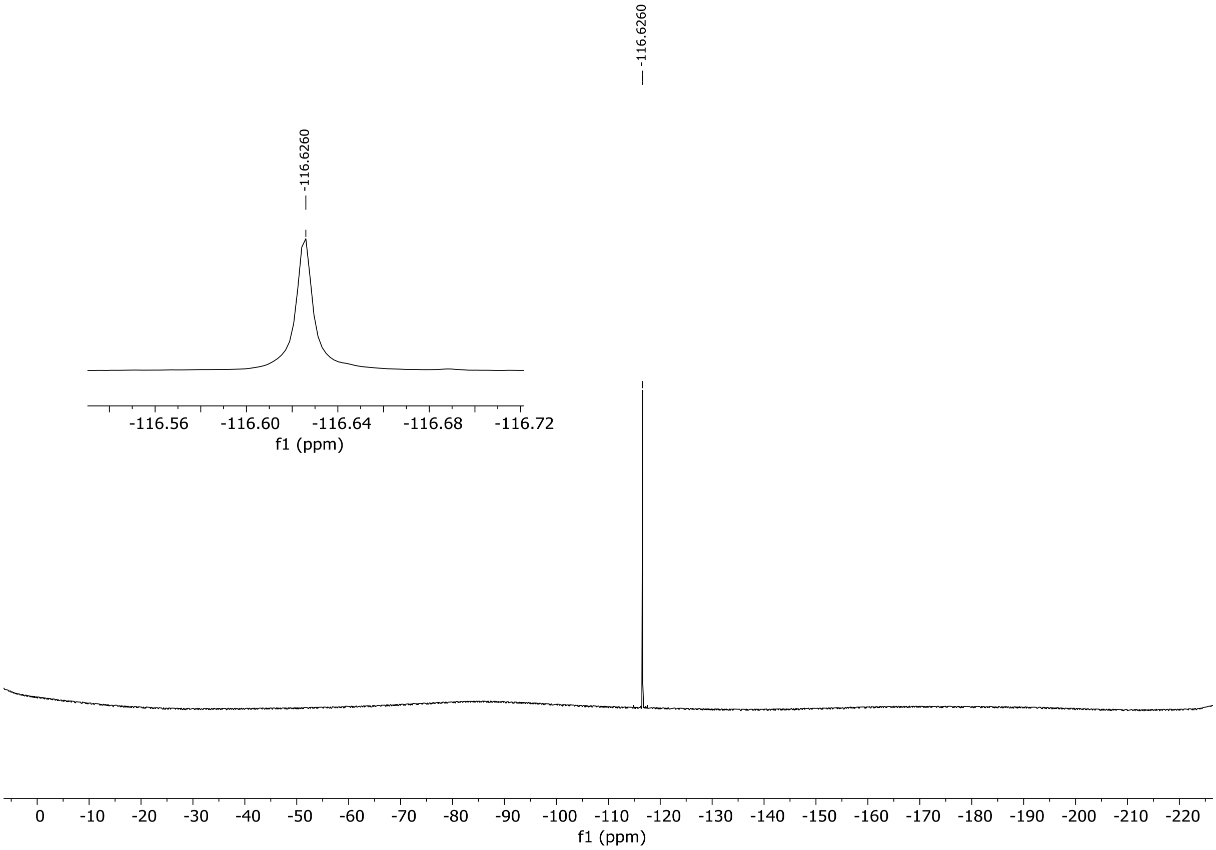

Compound **1e**

^{1}H-NMR (300 MHz, CD_2Cl_2)

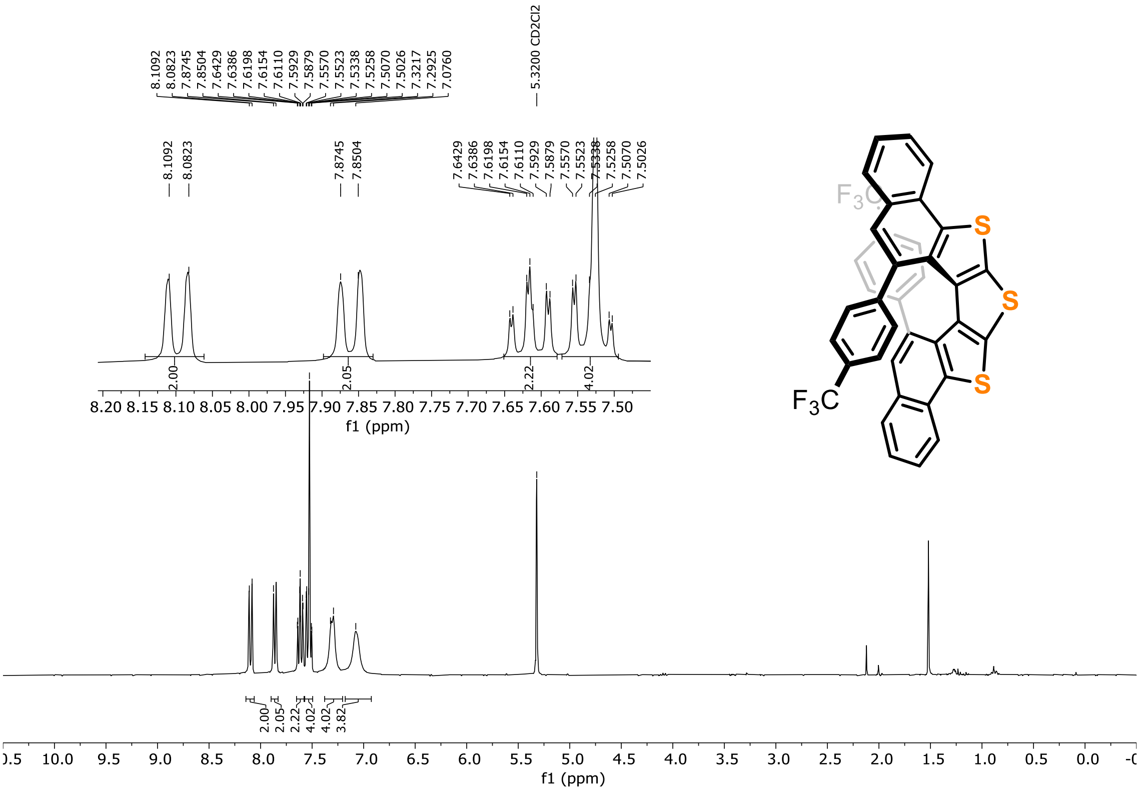

$^{13}C\{^{1}H\}$-NMR (101 MHz, CD_2Cl_2)

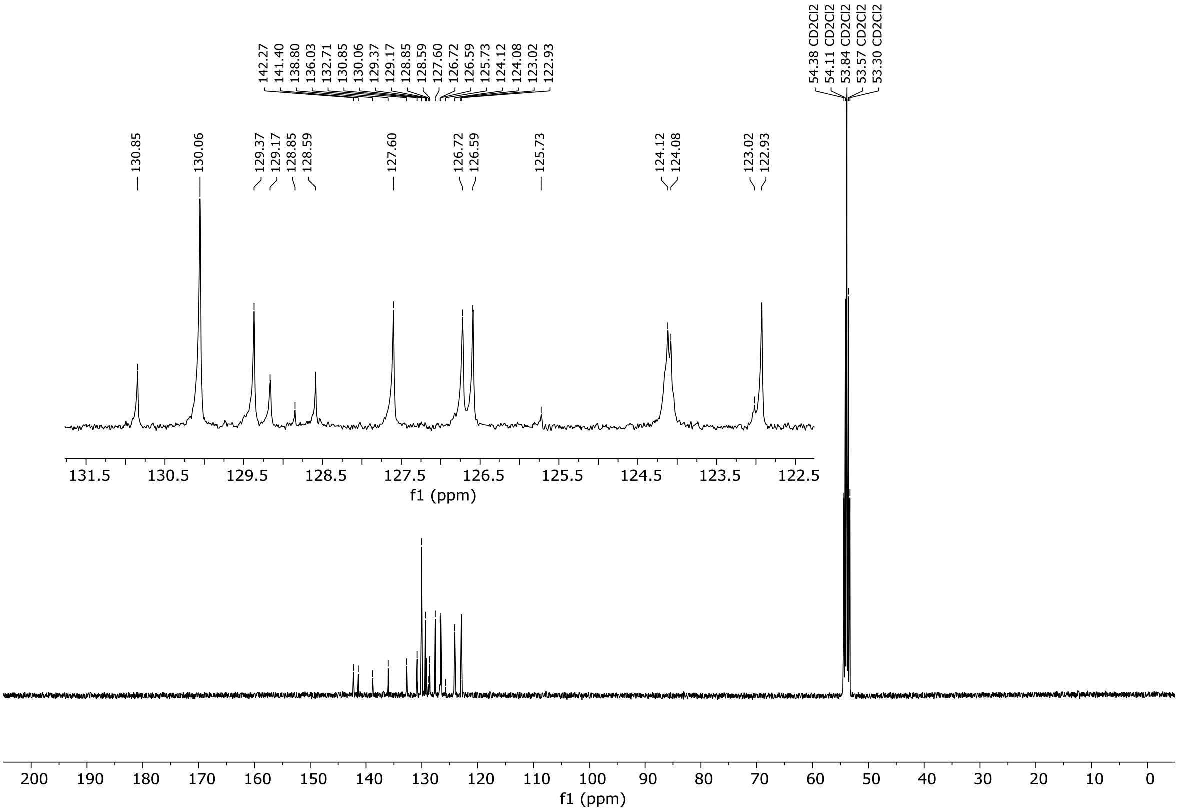

^{19}F-NMR (282 MHz, CD_2Cl_2)

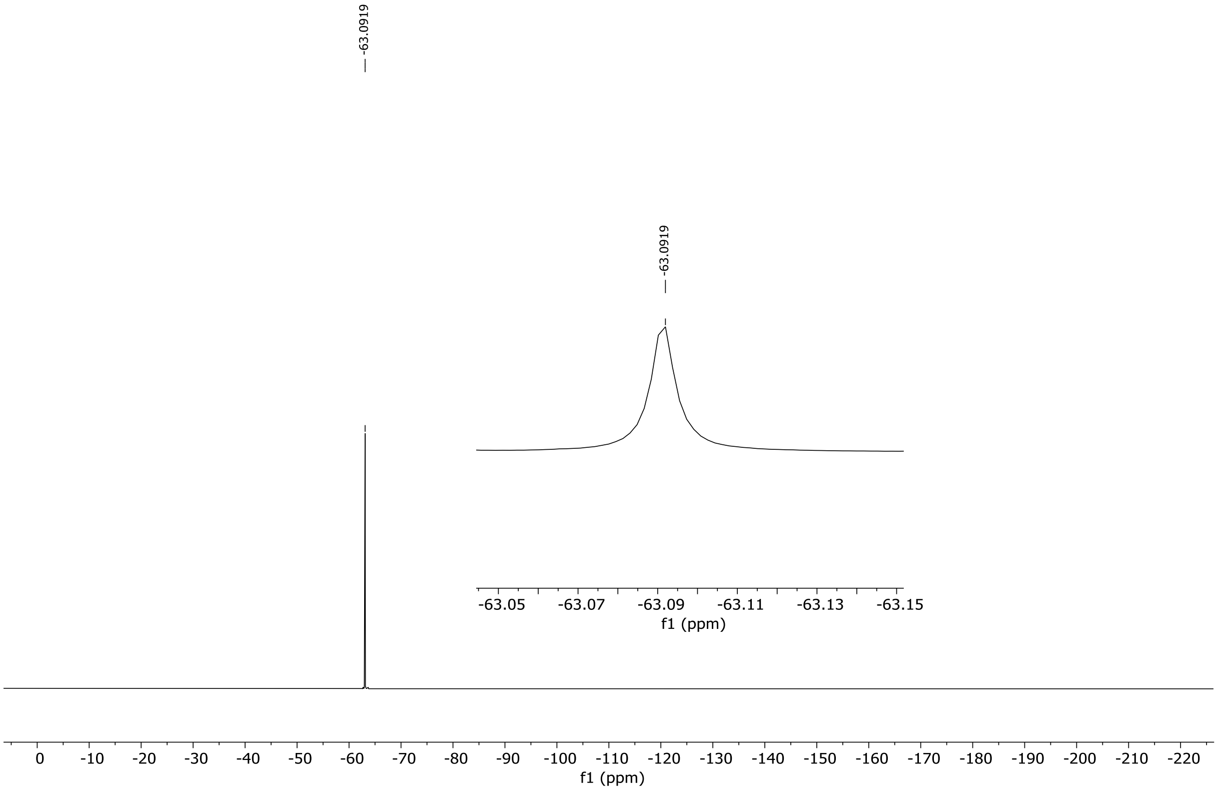

Compound **1f**

^{1}H-NMR (300 MHz, $CDCl_3$)

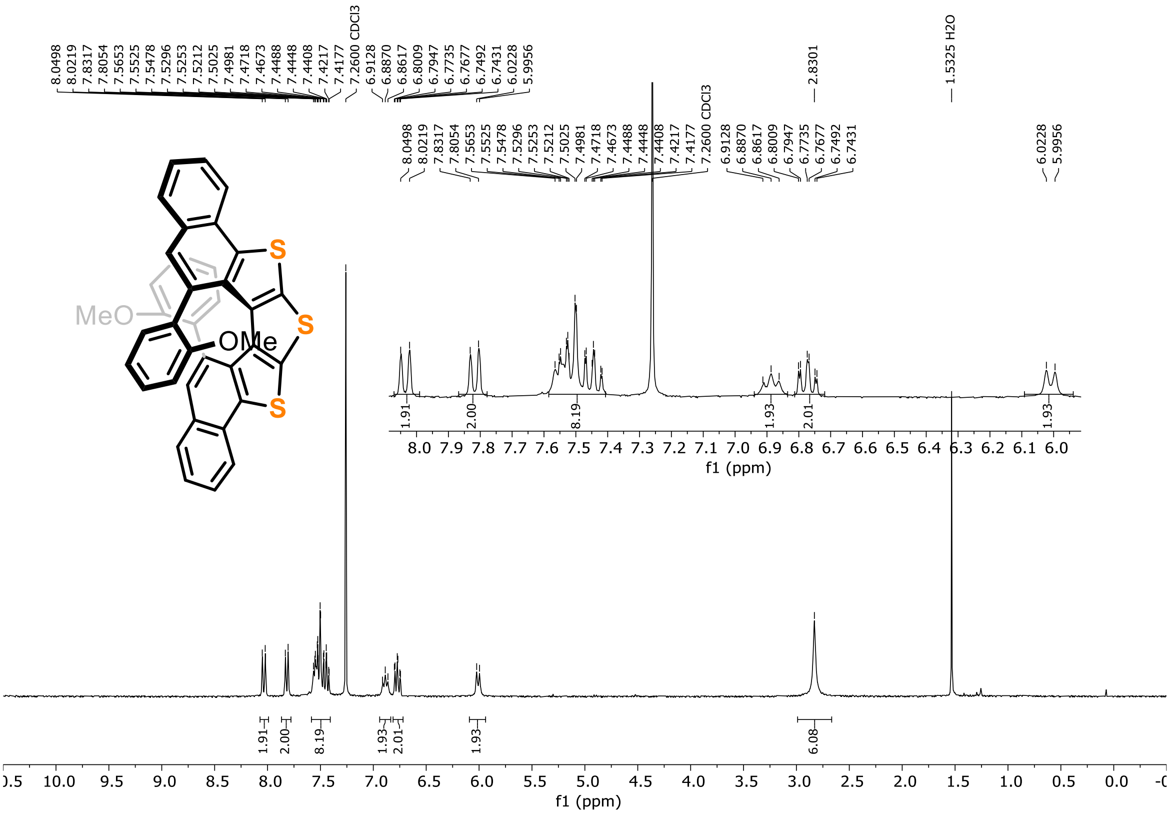

^{13}C{^{1}H}-NMR (101 MHz, $CDCl_3$)

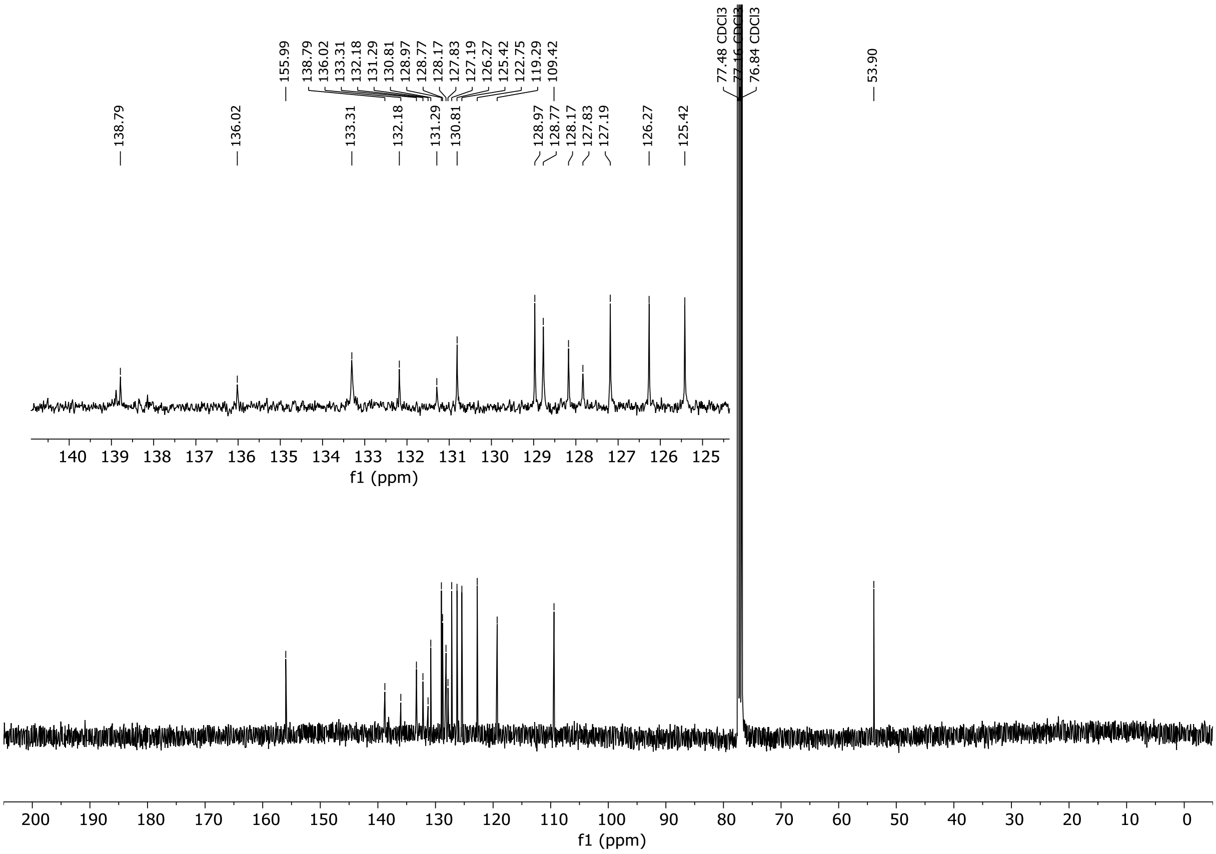

Compound **1g**

^{1}H-NMR (300 MHz, CDCl$_3$)

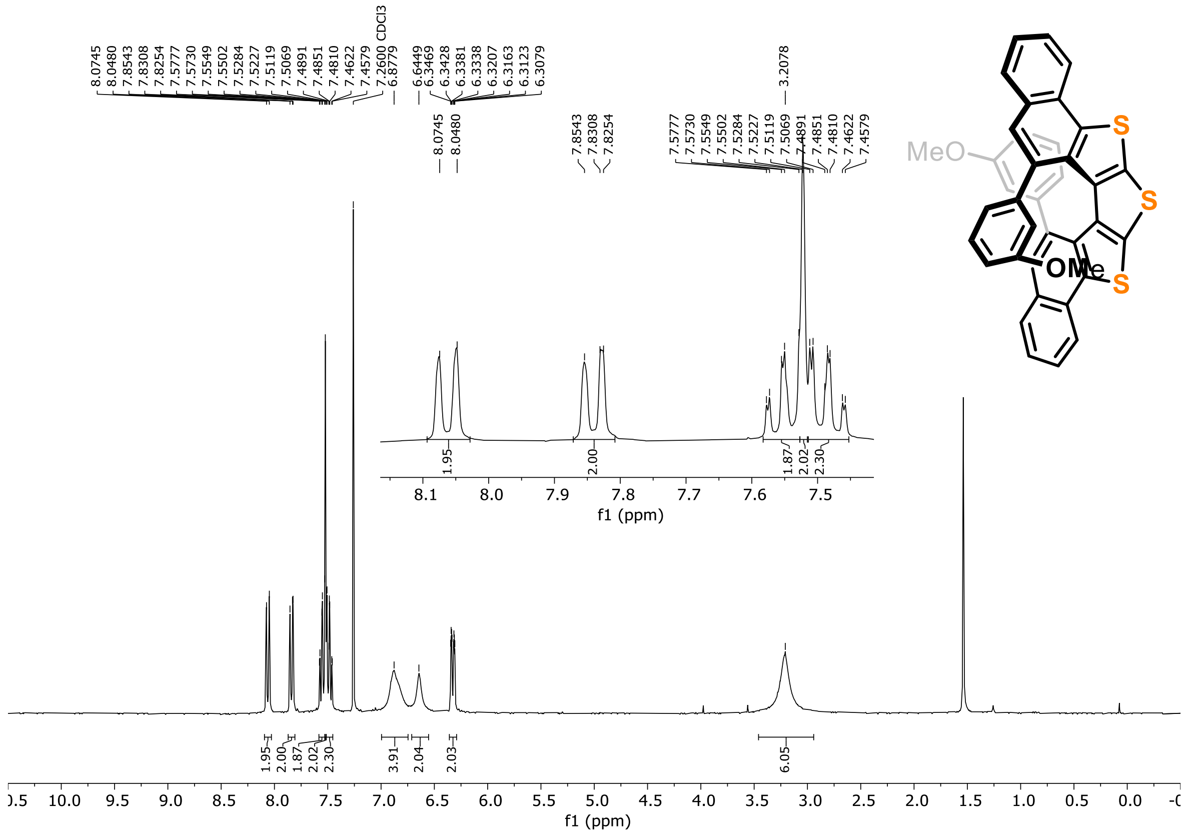

^{13}C{^{1}H}-NMR (101 MHz, CDCl$_3$)

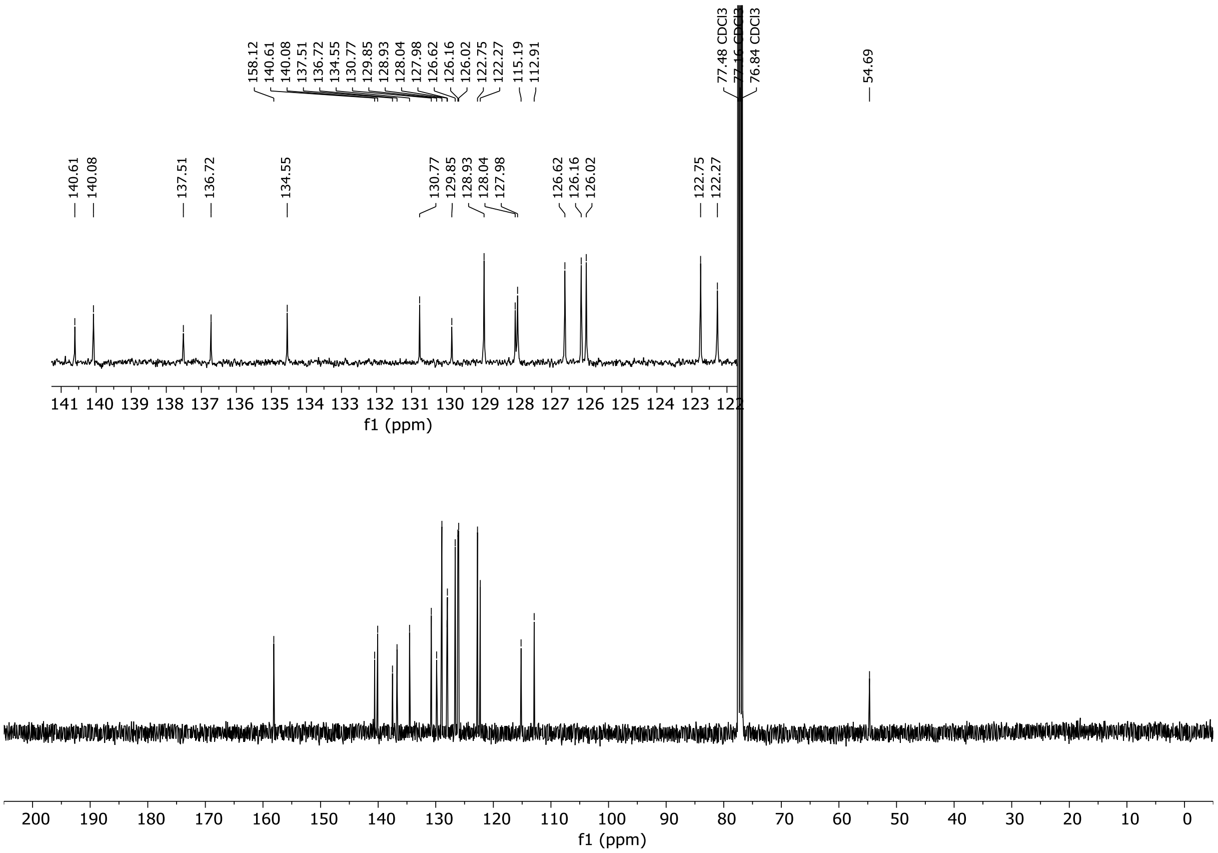

Compound **1h**

^{1}H-NMR (300 MHz, $CDCl_3$)

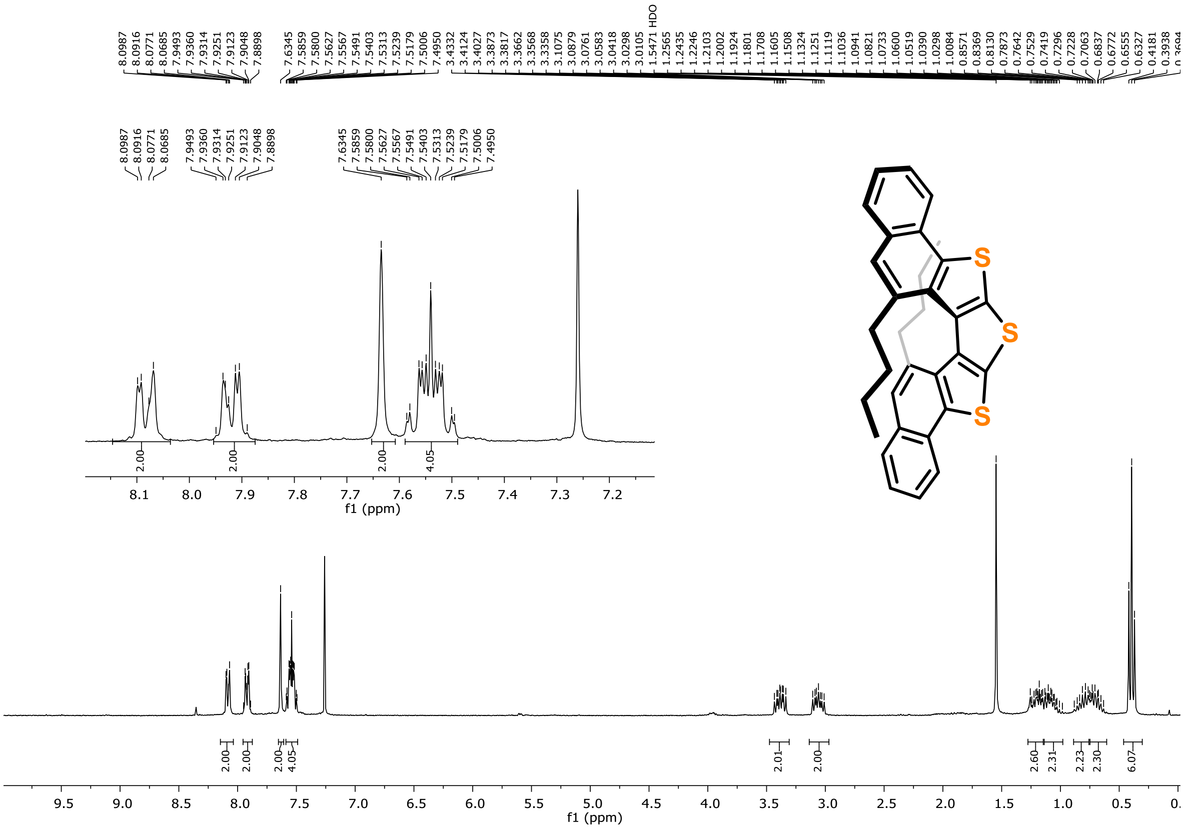

^{13}C{^{1}H}-NMR (101 MHz, $CDCl_3$)

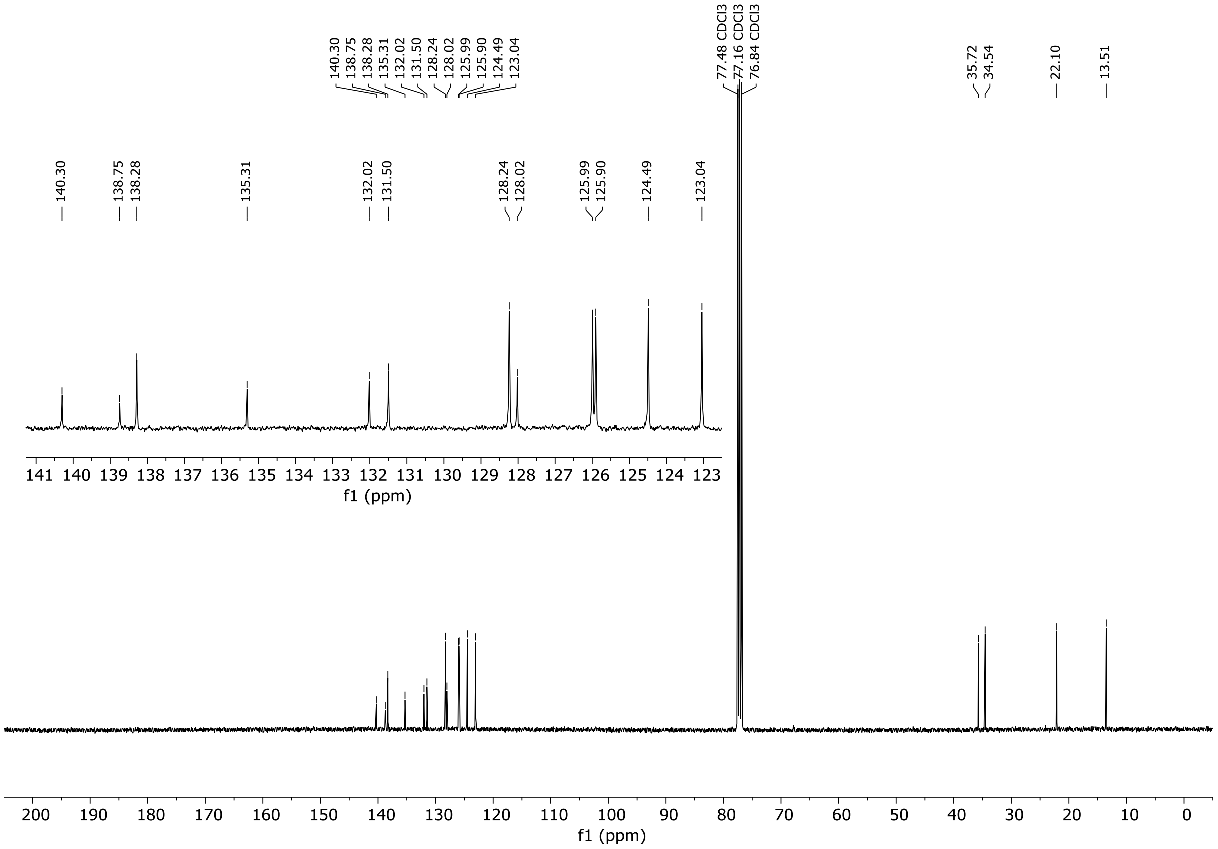

Compound **5a**

^{1}H-NMR (300 MHz, $CDCl_3$)

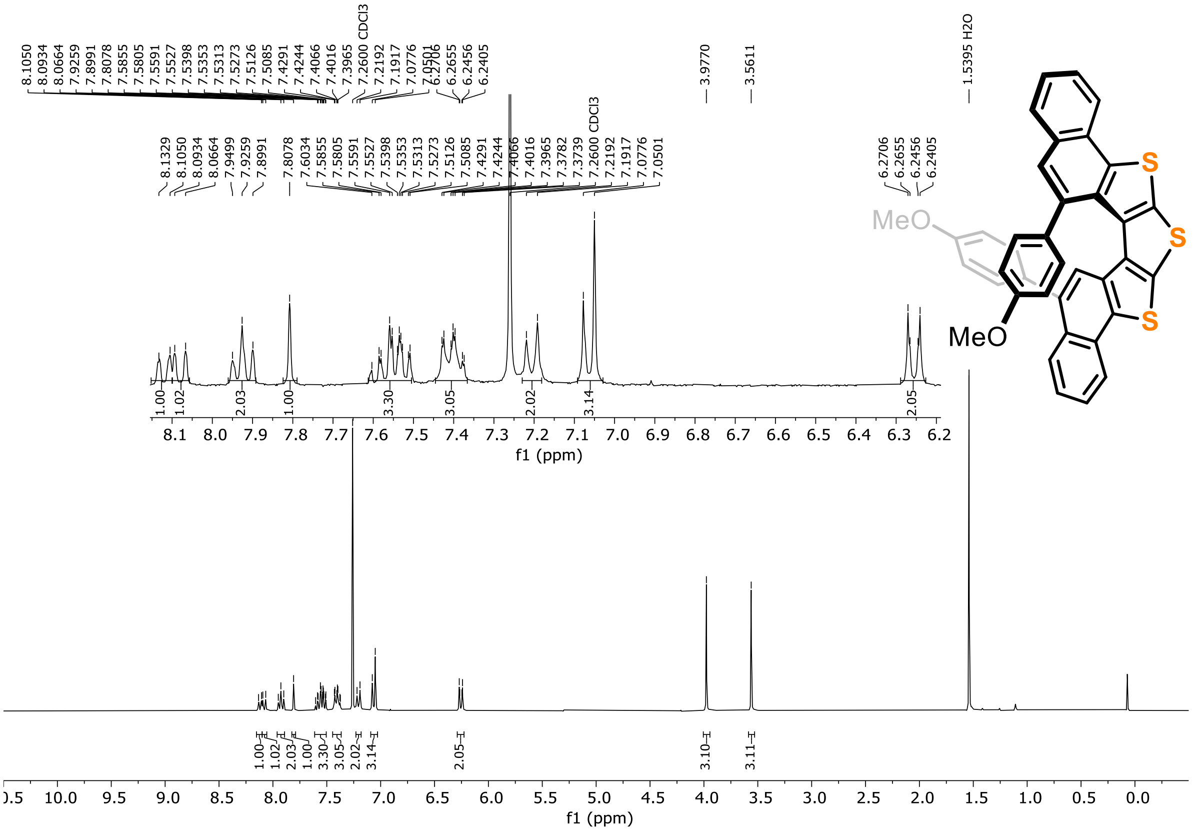

^{13}C{^{1}H}-NMR (126 MHz, $CDCl_3$)

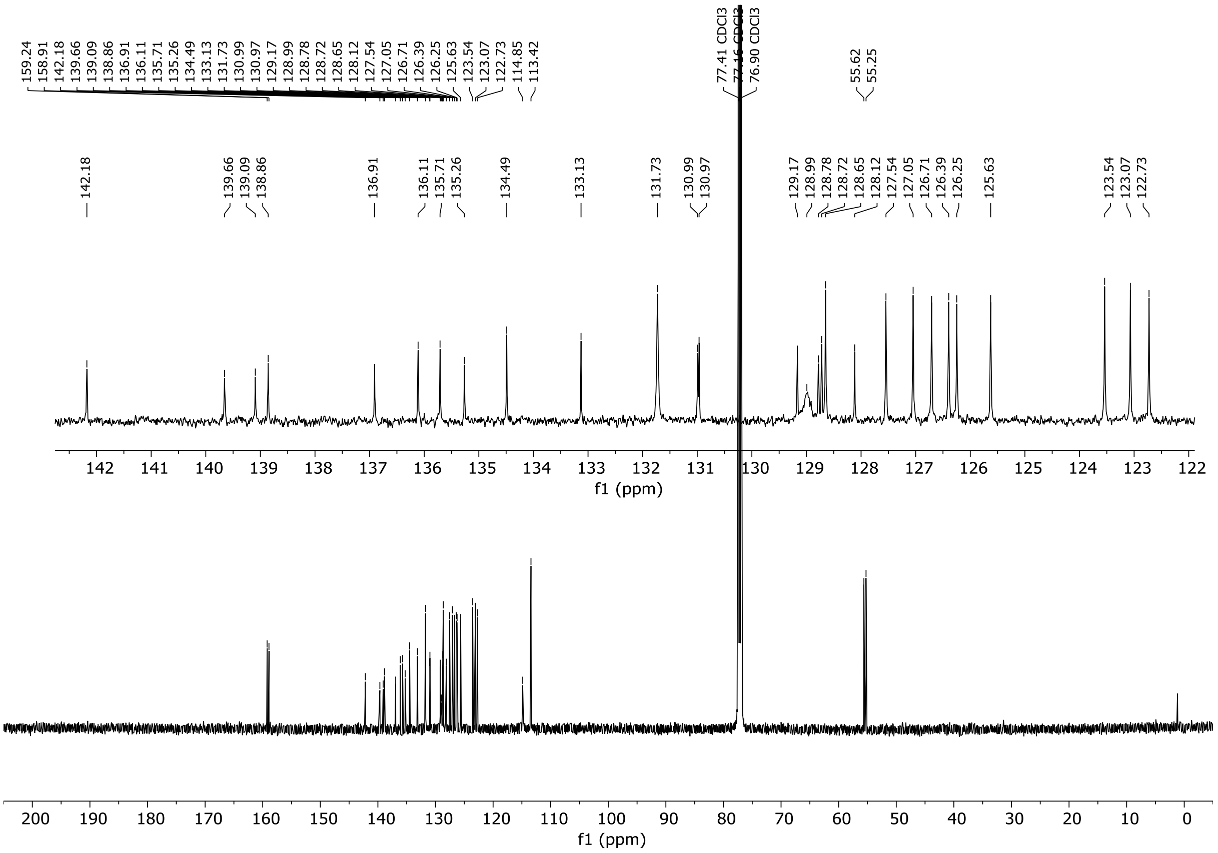

Compound **8a**

^{1}H-NMR (300 MHz, $CDCl_3$)

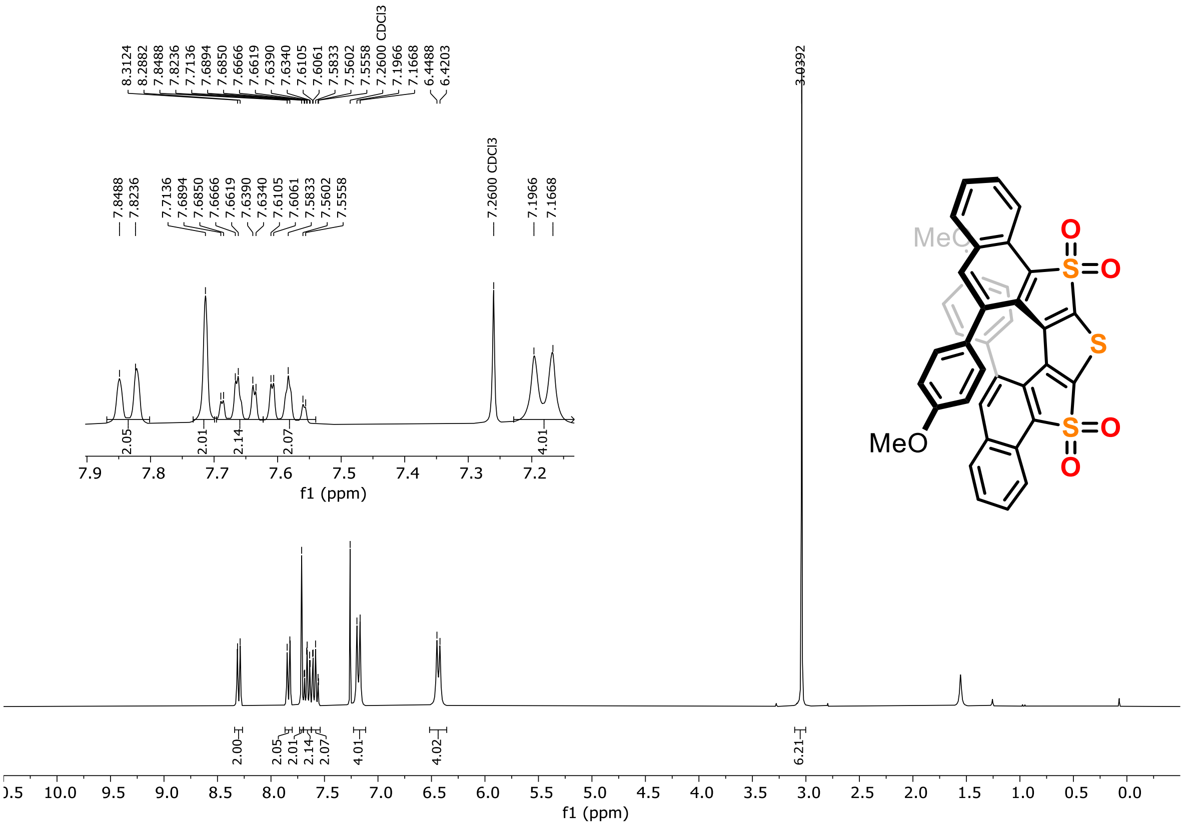

^{13}C{^{1}H}-NMR (101 MHz, $CDCl_3$)

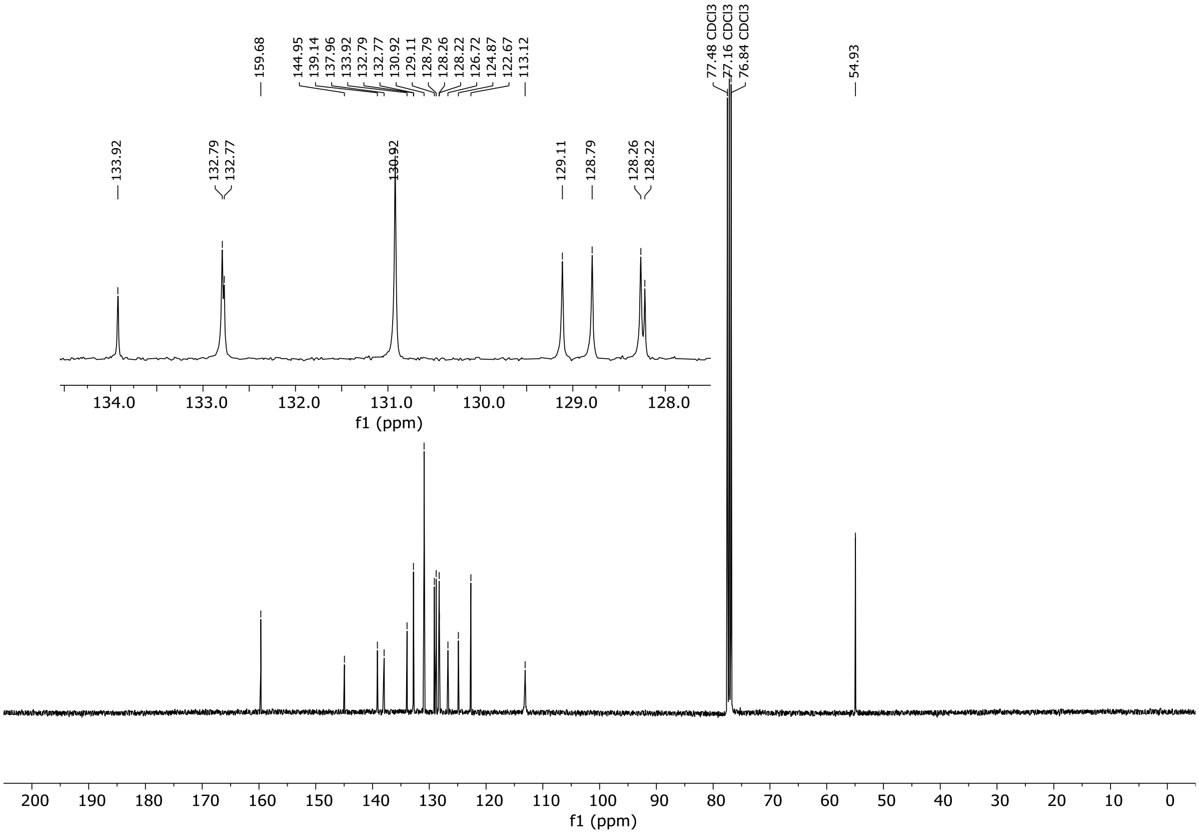

Compound **9a**

^{1}H-NMR (400 MHz, $CDCl_3$)

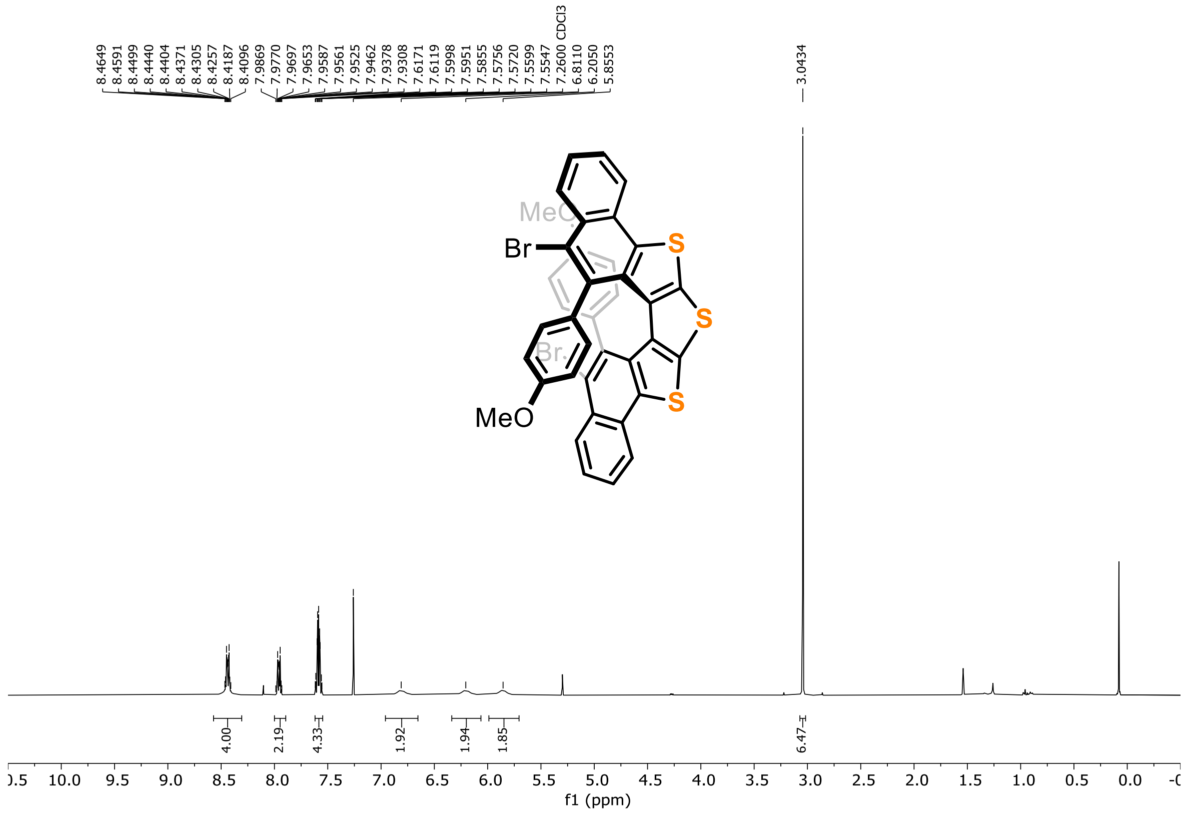

^{13}C{^{1}H}-NMR (101 MHz, $CDCl_3$)

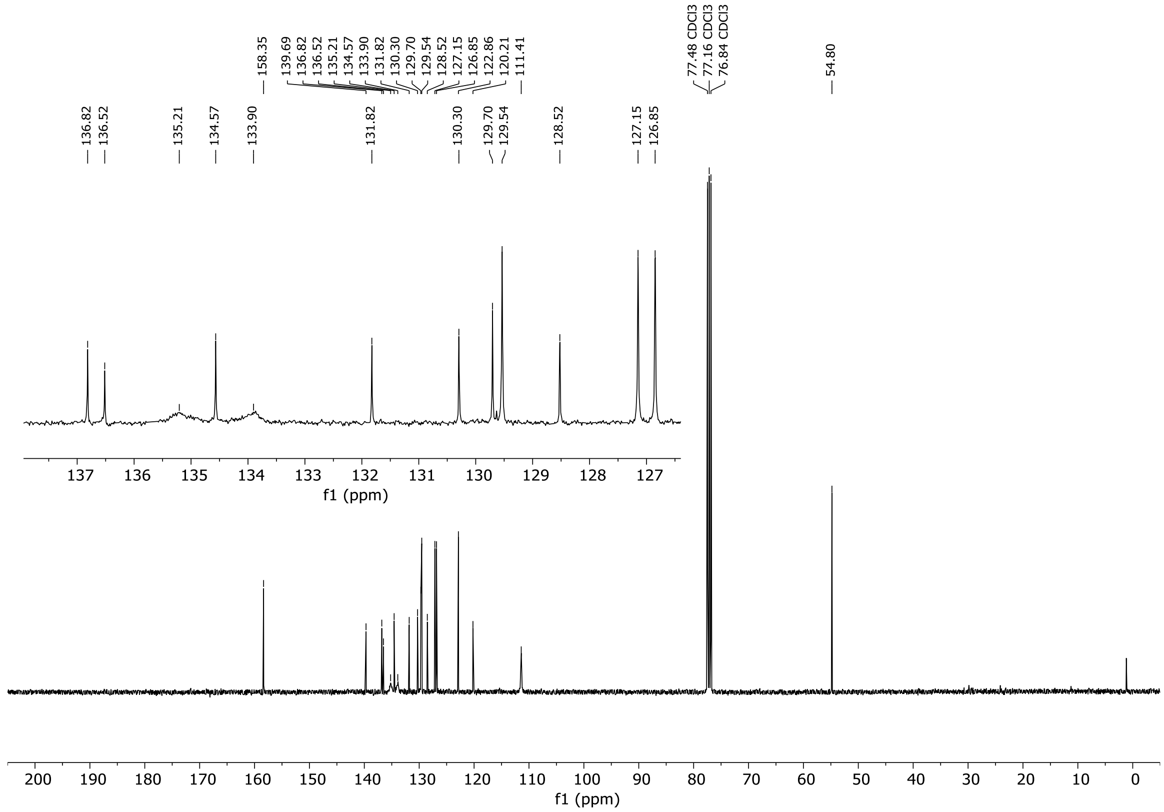

Compound **11a**

^{1}H-NMR (500 MHz, $CDCl_3$)

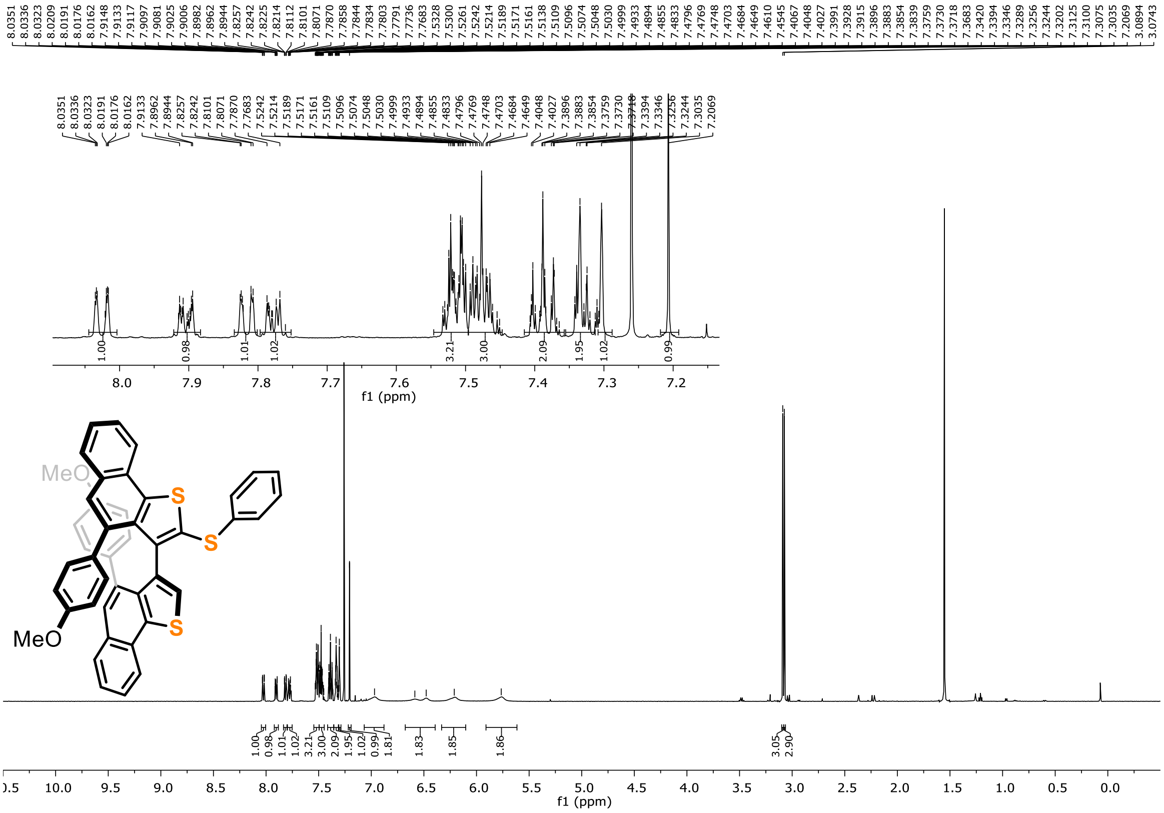

^{13}C{^{1}H}-NMR (126 MHz, $CDCl_3$)

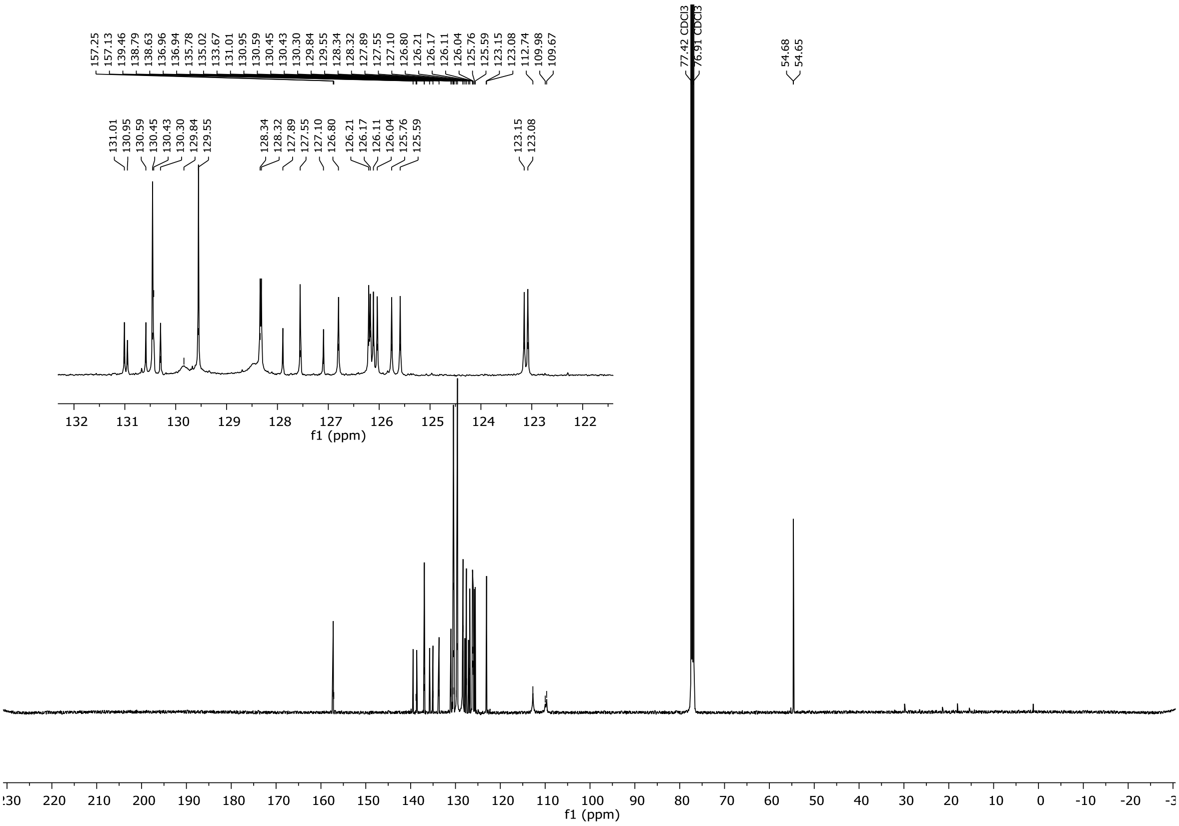

Compound **10a**

^{1}H-NMR (300 MHz, $CDCl_3$)

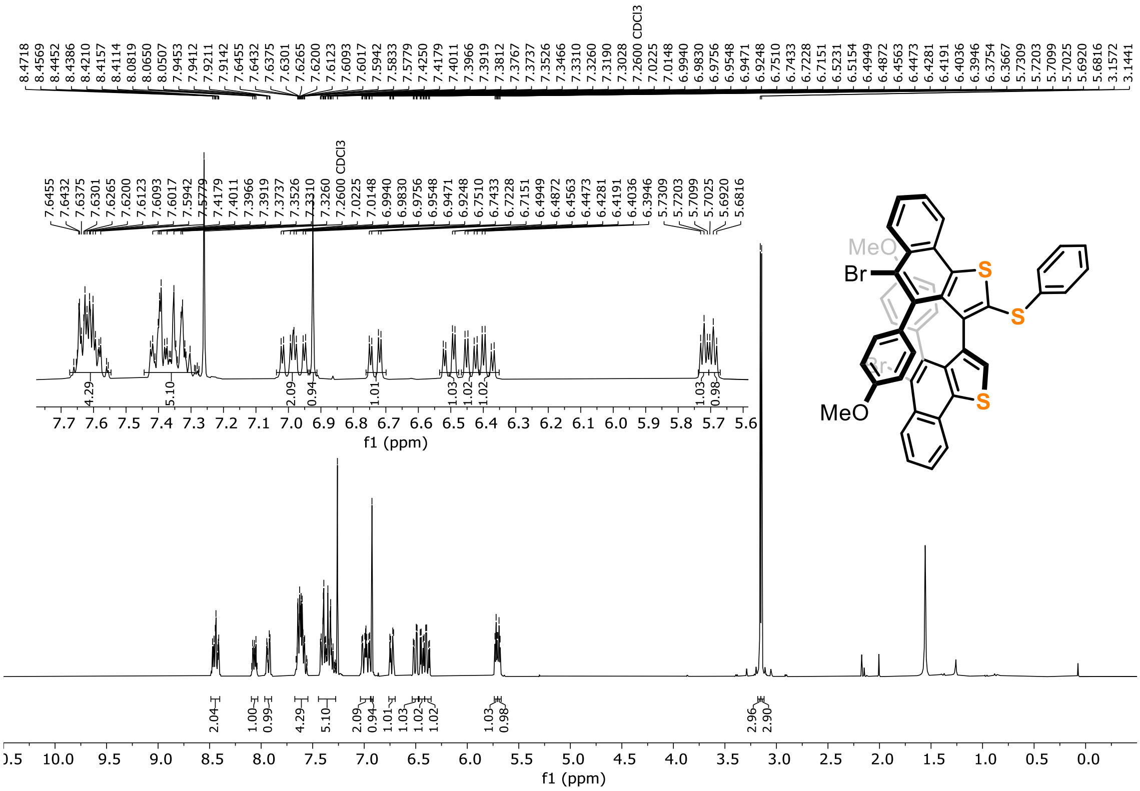

^{13}C{^{1}H}-NMR (126 MHz, $CDCl_3$)

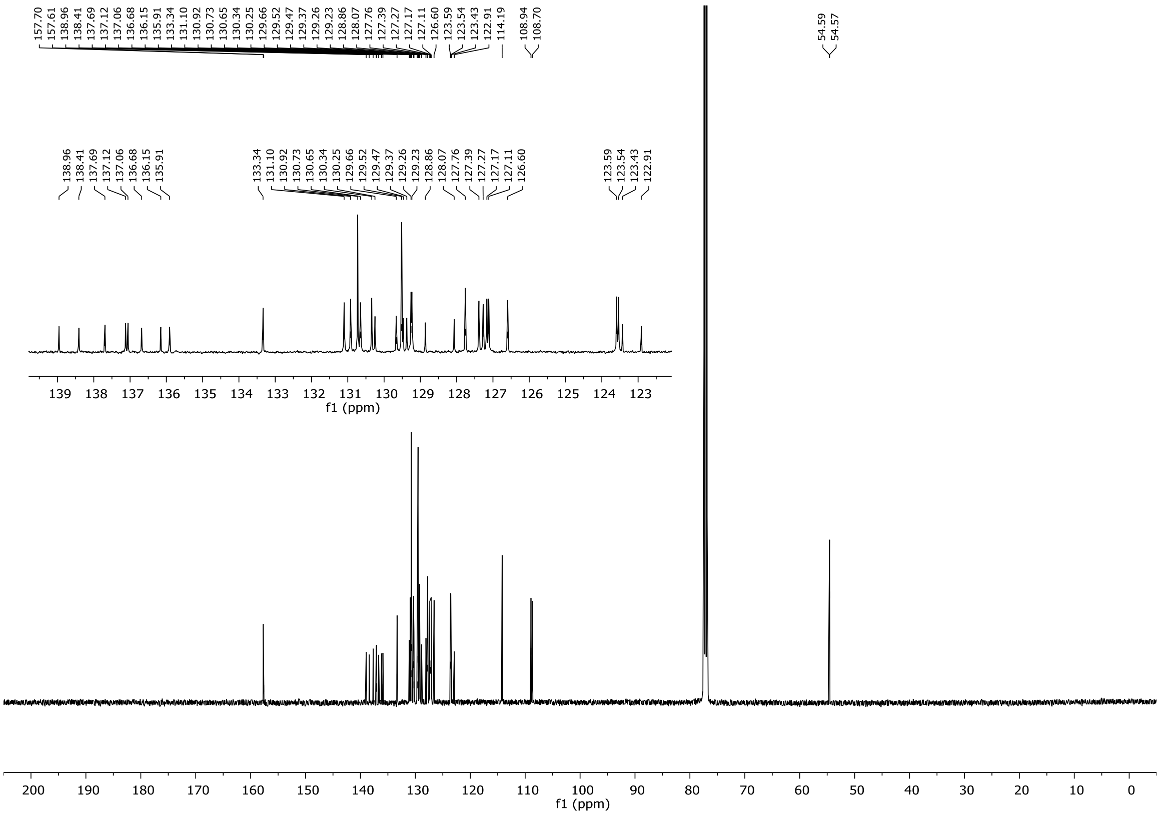

HPLC-Chromatograms

1a

Column: CHIRALPAK IA-3 SFC (3.0 mm × 100 mm). Eluent: CO_2:IPA = 65:35

<u>Racemic-</u> 1a

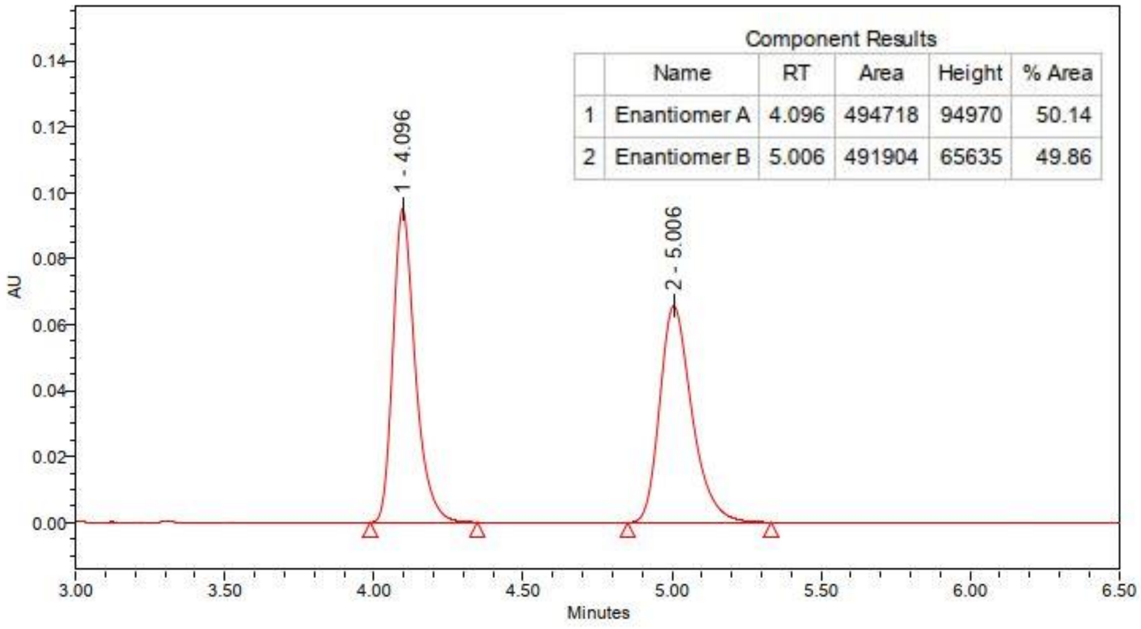

<u>Enantioenriched-</u> 1a 91% *ee*

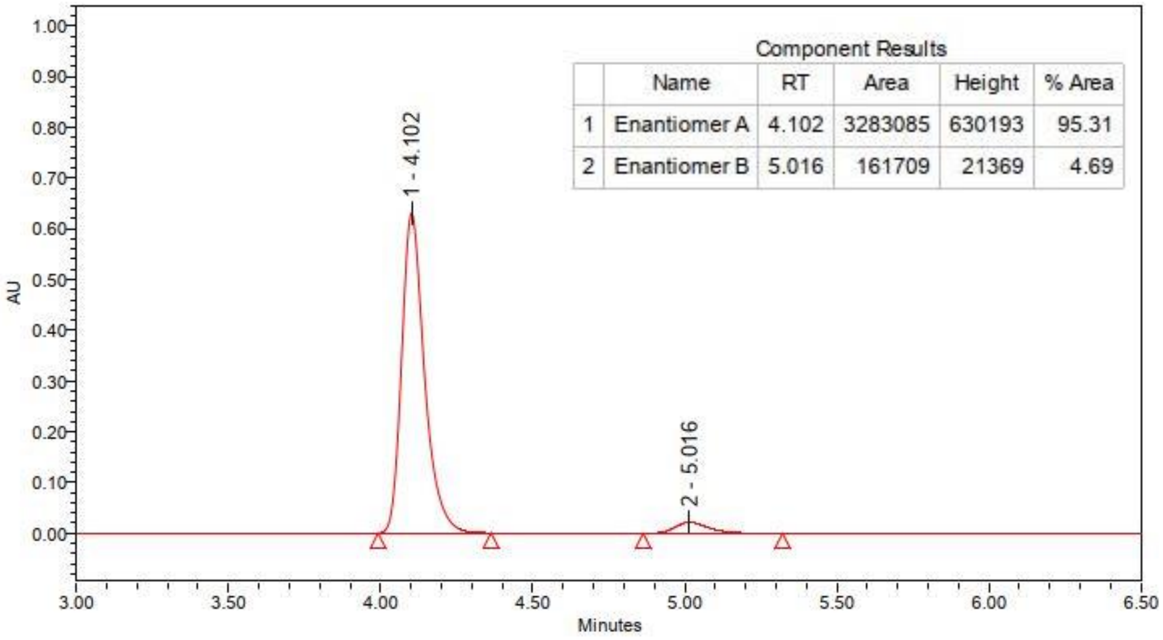

1b

Column: CHIRALPAK IG-U (3.0 mm × 100 mm). Eluent: acetonitrile:H_2O = 95:5

Racemic- 1b

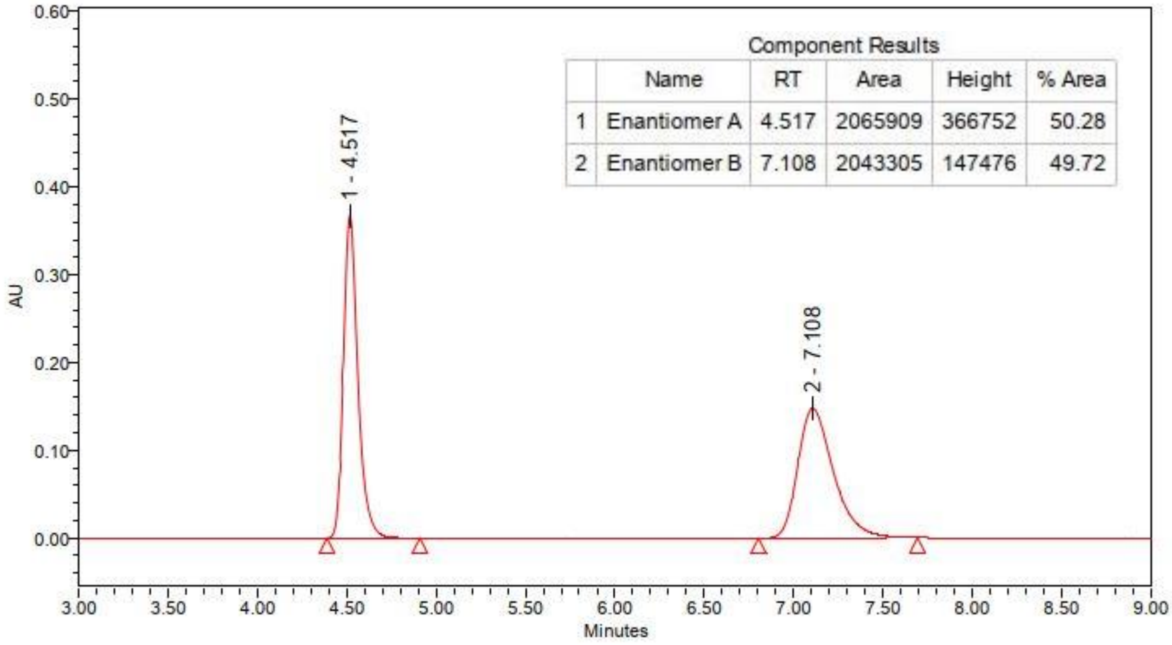

Component Results

	Name	RT	Area	Height	% Area
1	Enantiomer A	4.517	2065909	366752	50.28
2	Enantiomer B	7.108	2043305	147476	49.72

Enantioenriched- 1b 89% *ee*

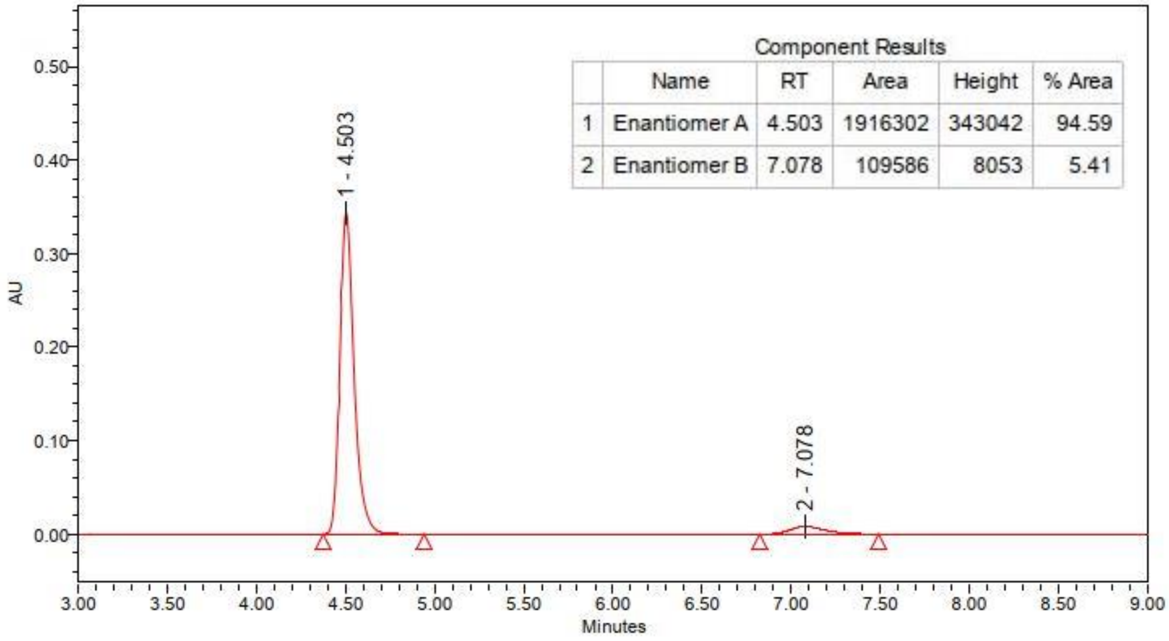

Component Results

	Name	RT	Area	Height	% Area
1	Enantiomer A	4.503	1916302	343042	94.59
2	Enantiomer B	7.078	109586	8053	5.41

1c

Column: CHIRALPAK IG-U (3.0 mm × 100 mm). Eluent: acetonitrile:H_2O = 95:5

<u>Racemic-</u> 1c

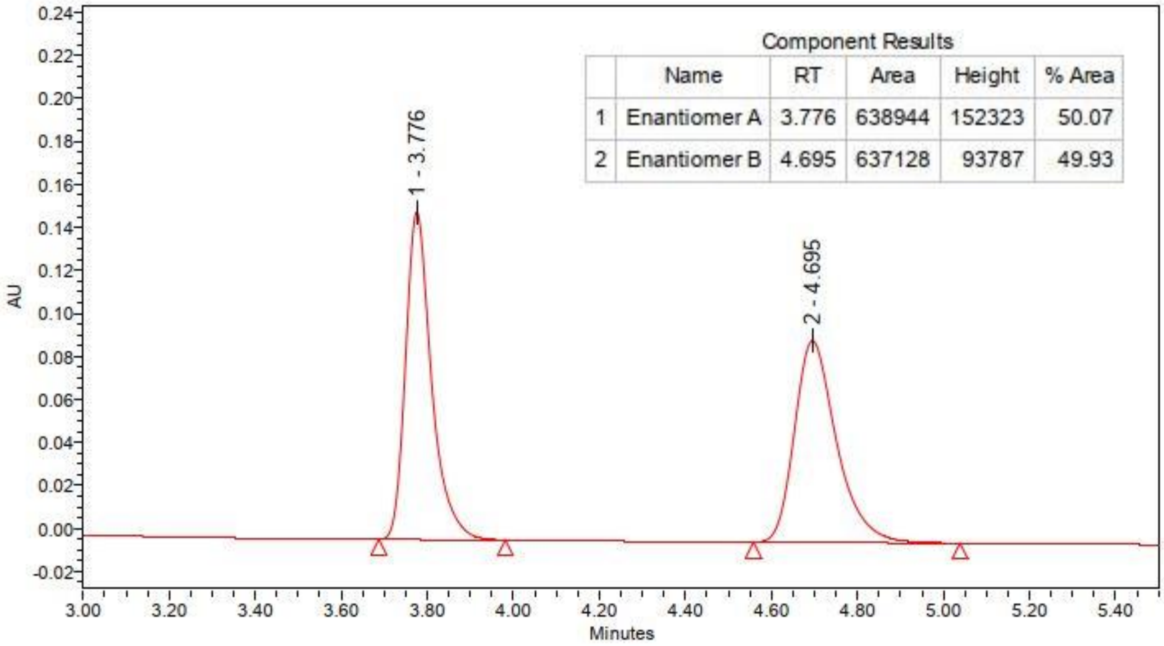

<u>Enantioenriched-</u> 1c 88% *ee*

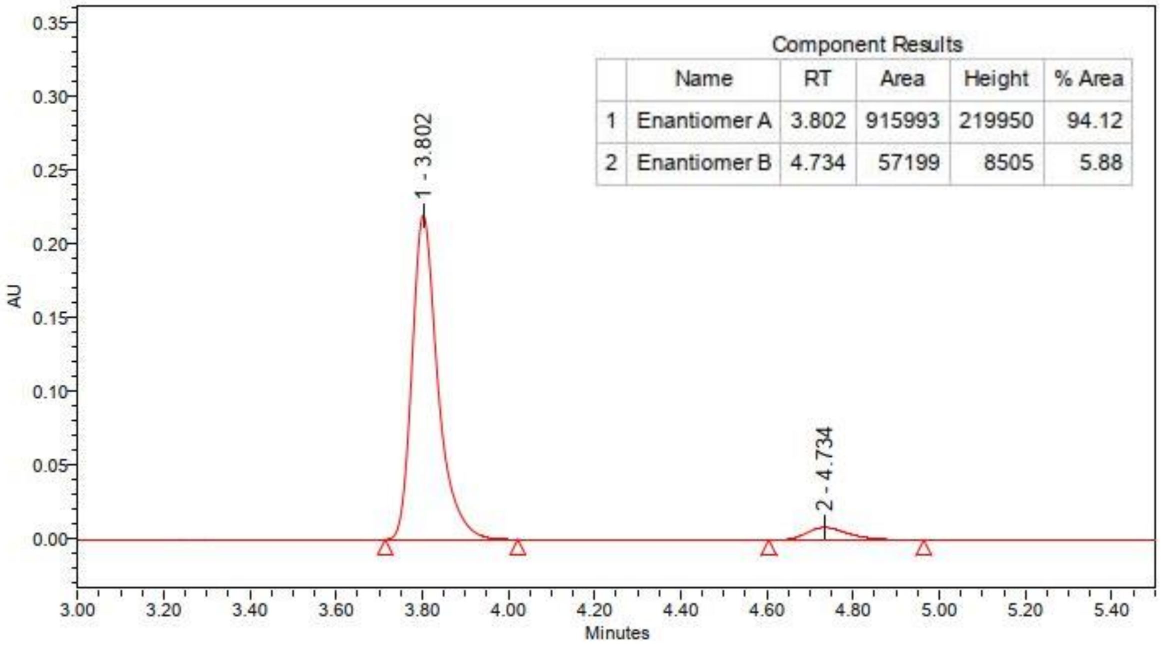

1d

Column: CHIRALPAK IG-U (3.0 mm × 100 mm). Eluent: acetonitrile:H_2O = 95:5

<u>Racemic-</u> 1d

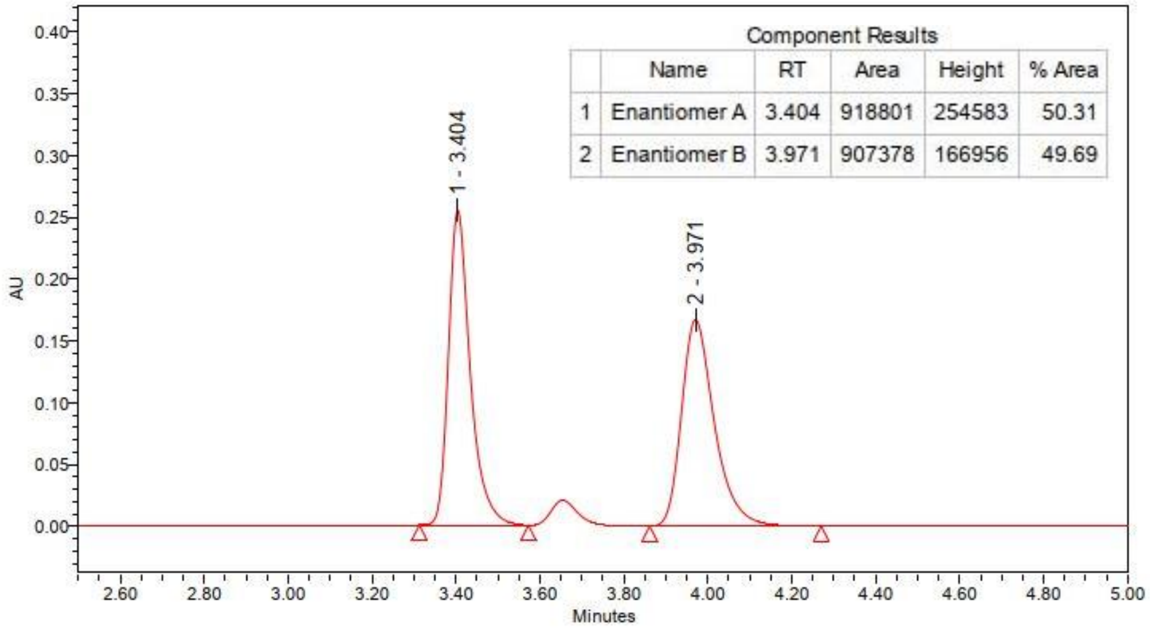

<u>Enantioenriched-</u> 1d 84% *ee*

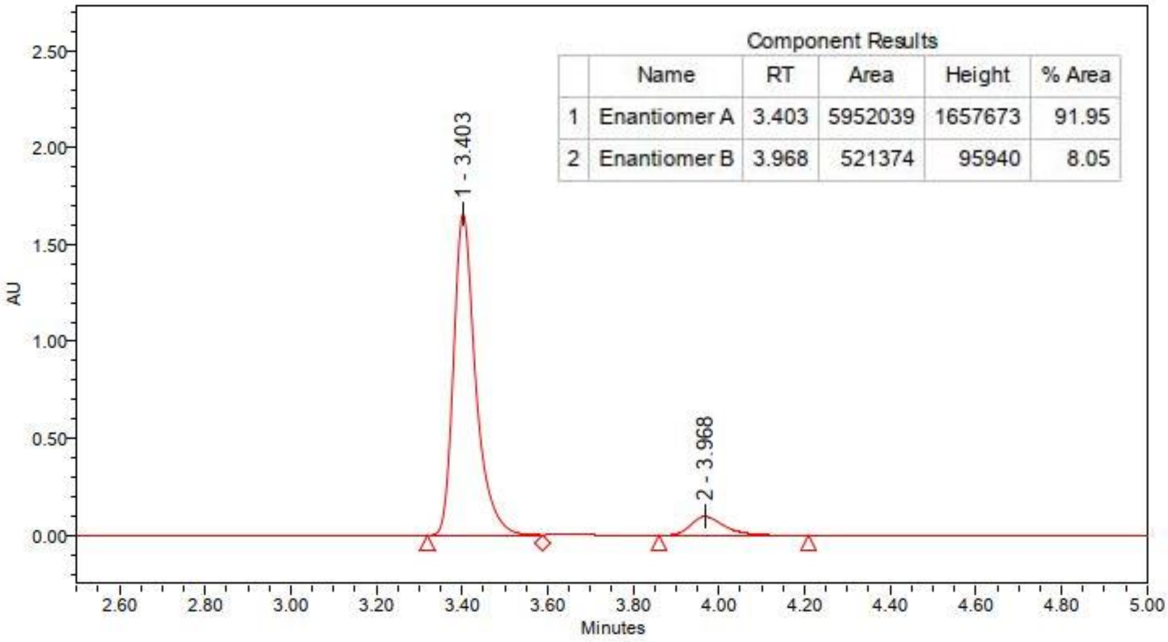

1e

Column: CHIRALPAK IG-U (3.0 mm × 100 mm). Eluent: acetonitrile:H_2O = 95:5

<u>Racemic-</u> 1e

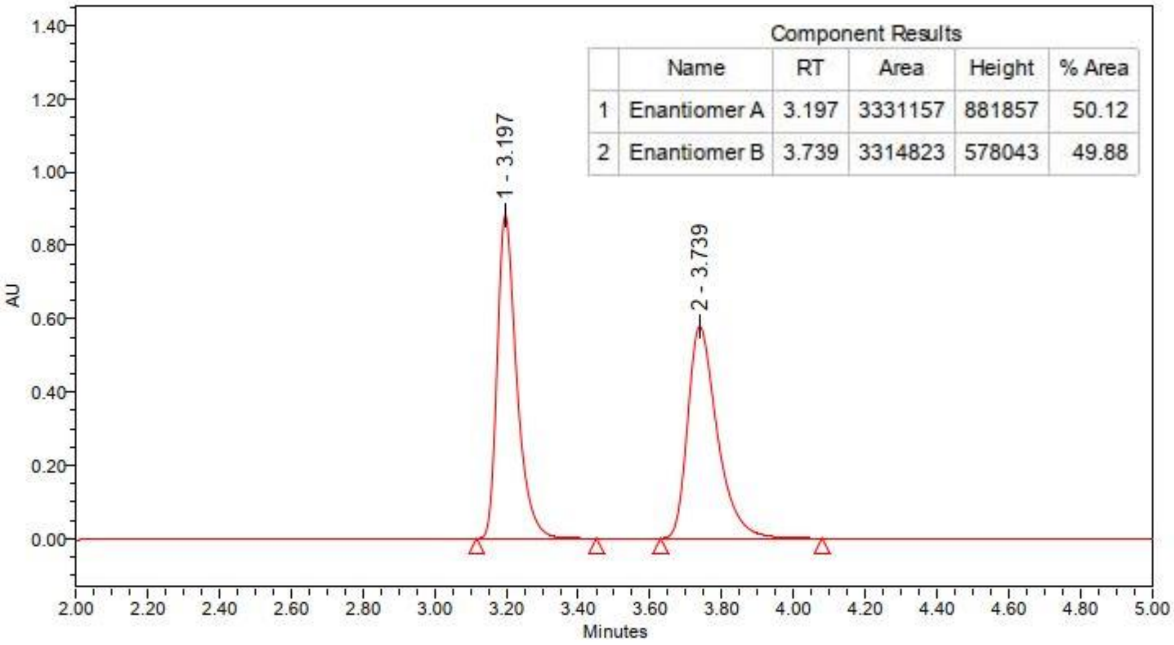

<u>Enantioenriched-</u> 1e 91% *ee*

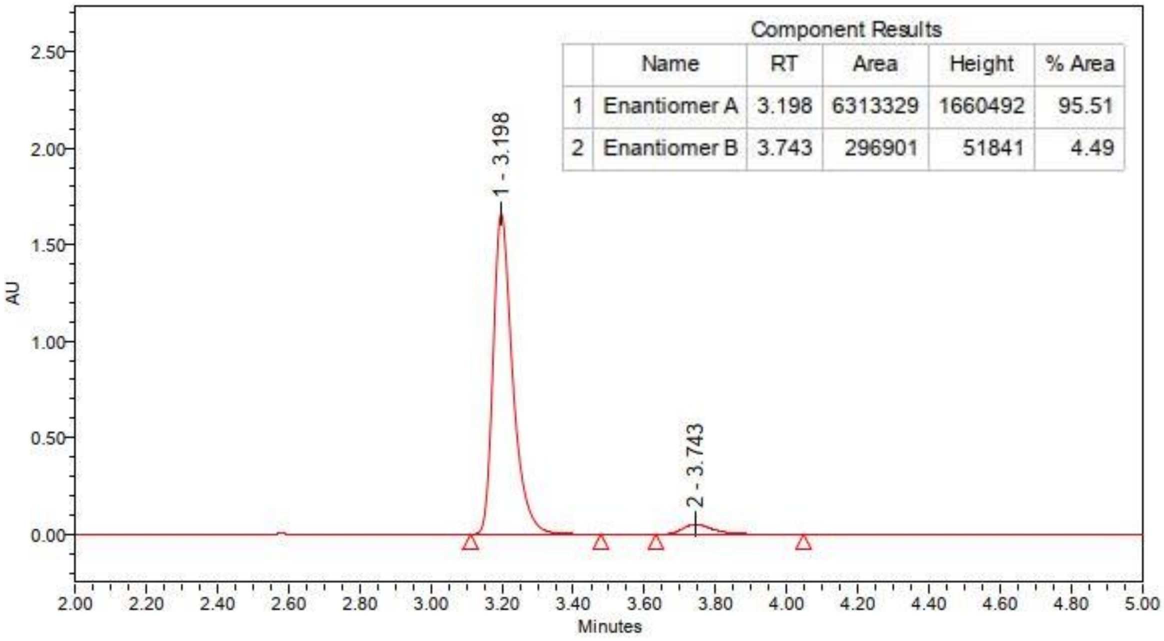

1f

Column: CHIRALPAK IA-3 SFC (3.0 mm × 100 mm). Eluent: CO_2:IPA = 70:30

<u>Racemic-</u> 1f

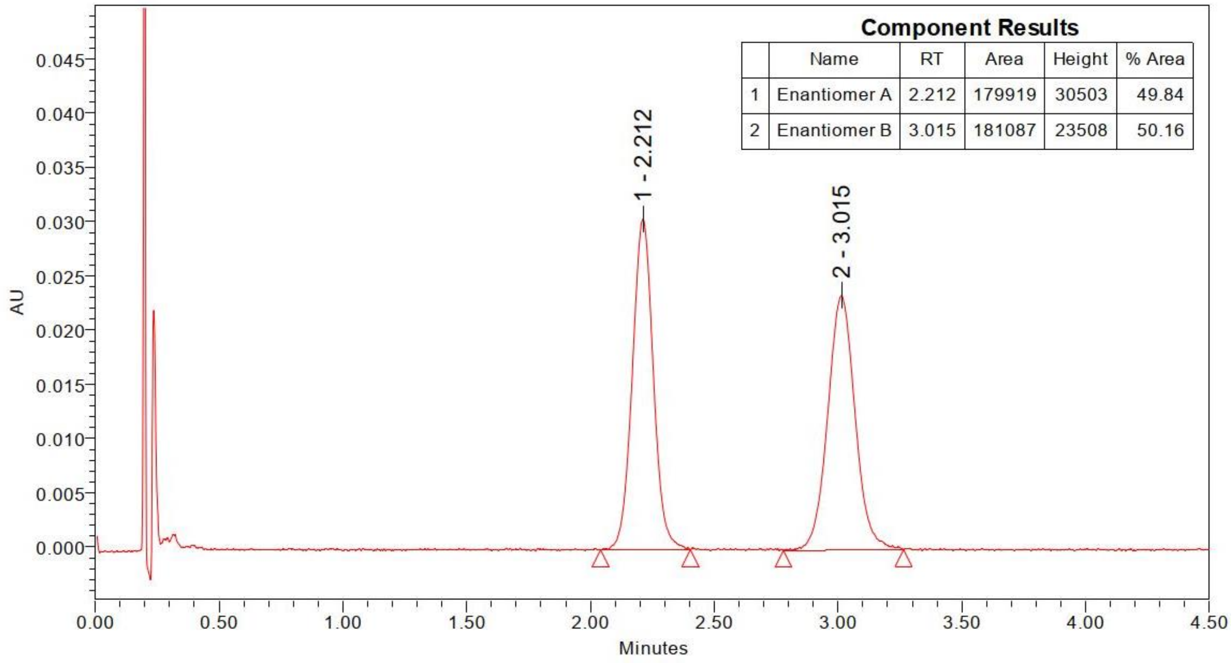

	Name	RT	Area	Height	% Area
1	Enantiomer A	2.212	179919	30503	49.84
2	Enantiomer B	3.015	181087	23508	50.16

<u>Enantioenriched-</u> 1f 87% *ee*

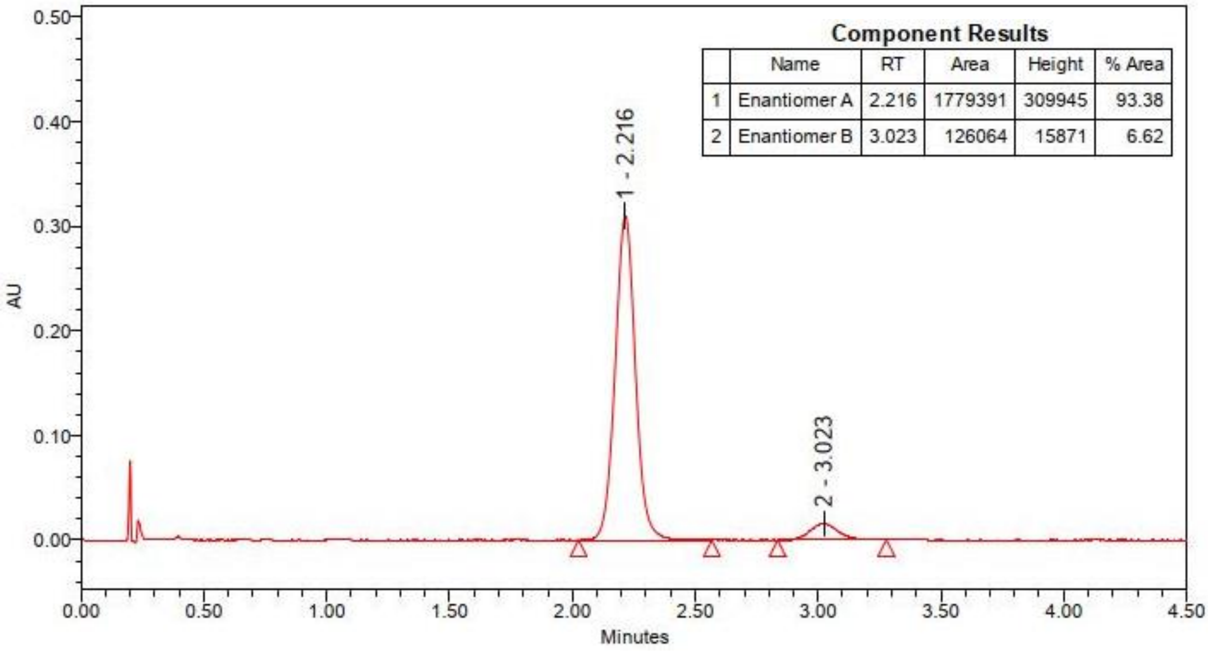

	Name	RT	Area	Height	% Area
1	Enantiomer A	2.216	1779391	309945	93.38
2	Enantiomer B	3.023	126064	15871	6.62

1g

Column: CHIRALPAK IA-3 SFC (3.0 mm × 100 mm). Eluent: CO_2:IPA = 65:35

<u>Racemic-</u> 1g

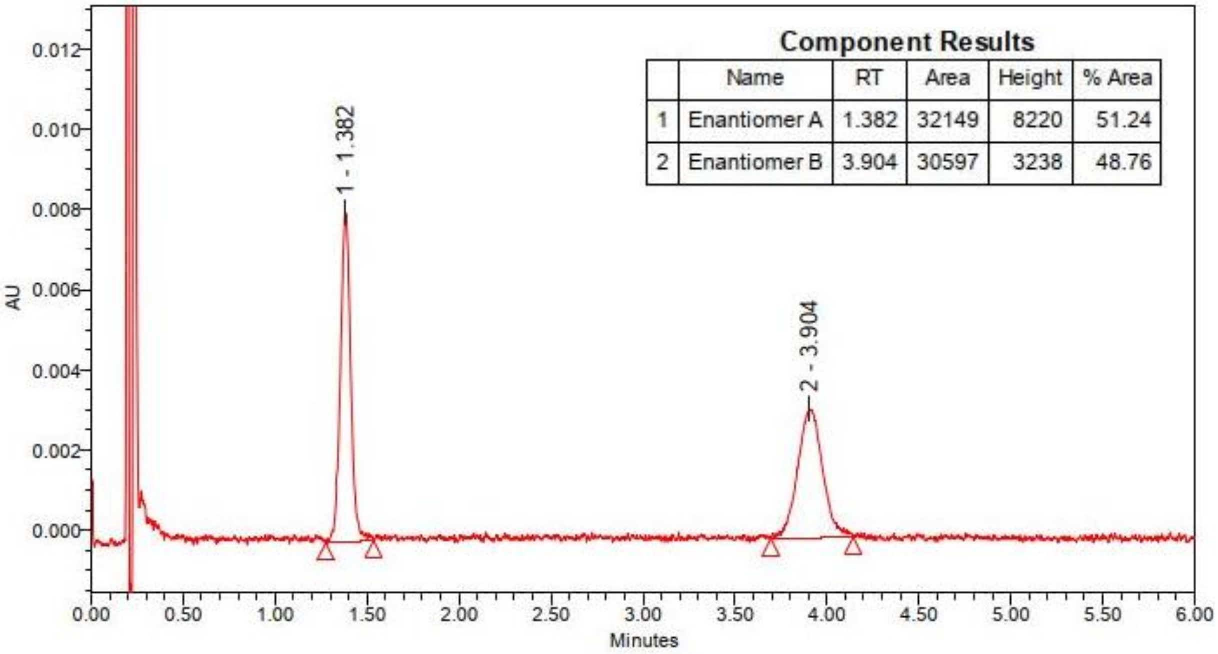

<u>Enantioenriched-</u> 1g 10% *ee*

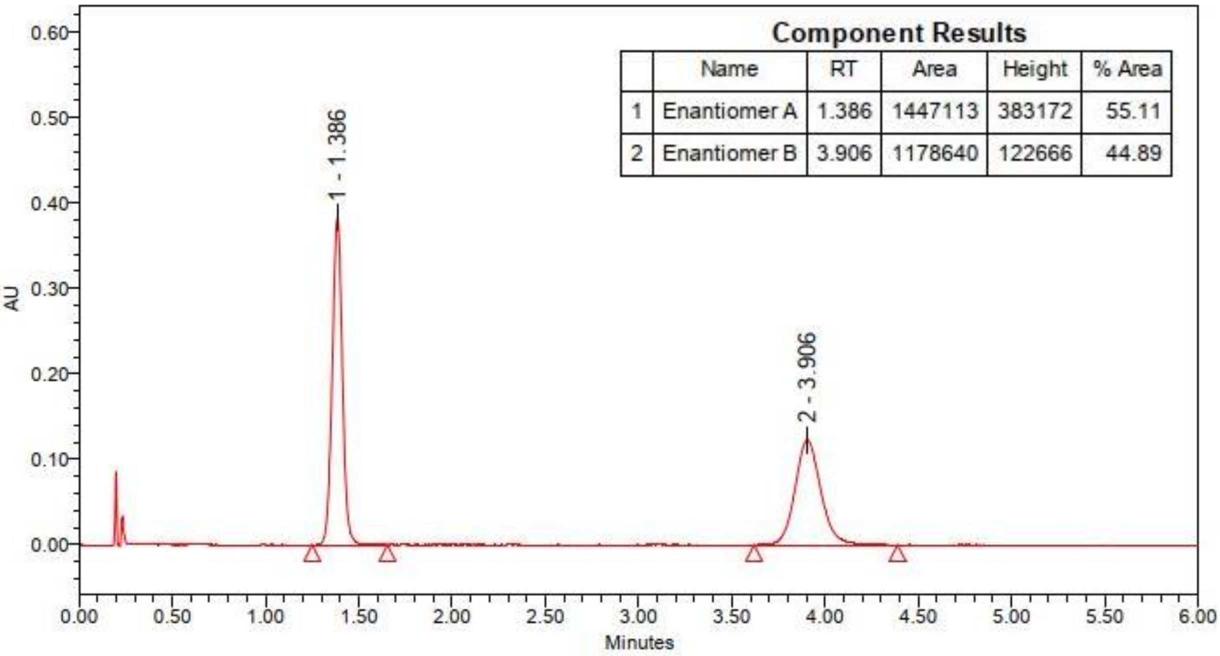

1h

Column: CHIRALPAK IB-3 SFC (3.0 mm × 100 mm). Eluent: CO_2:IPA = 90:10

<u>Racemic-</u> 1h

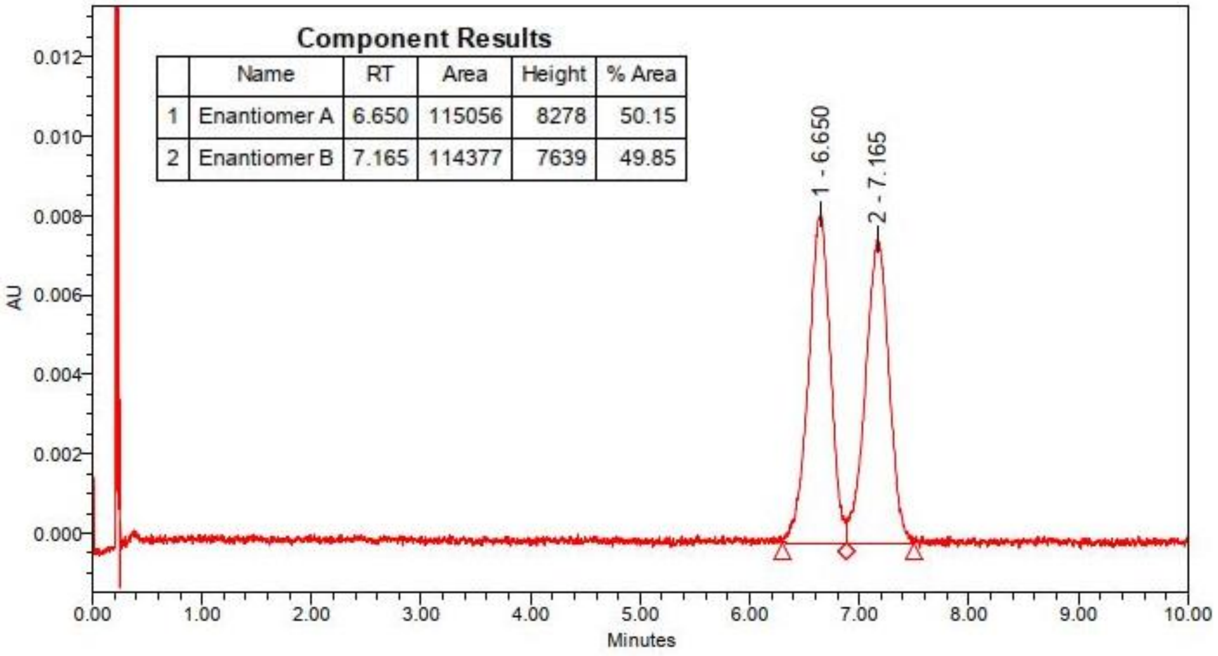

<u>Enantioenriched-</u> 1h 59% *ee*

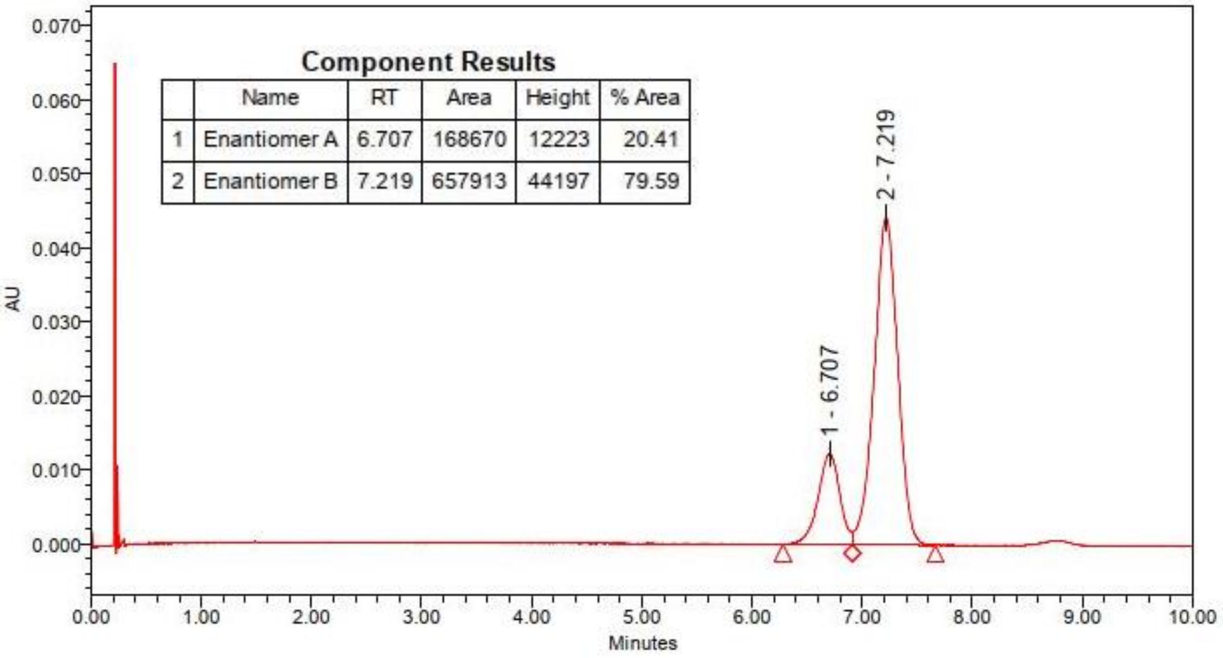

8a

Column: CHIRALPAK IA-3 SFC (3.0 mm × 100 mm). Eluent: CO_2:IPA = 80:20

Racemic- 8a

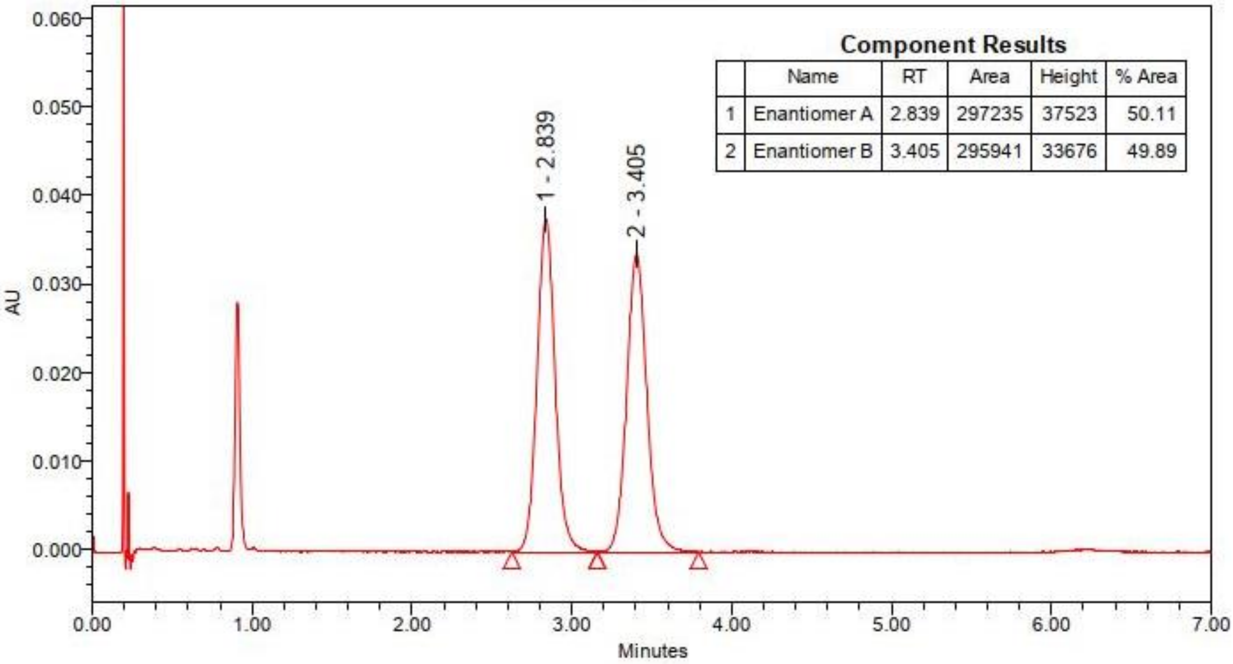

Enantioenriched- 8a 92% *ee*

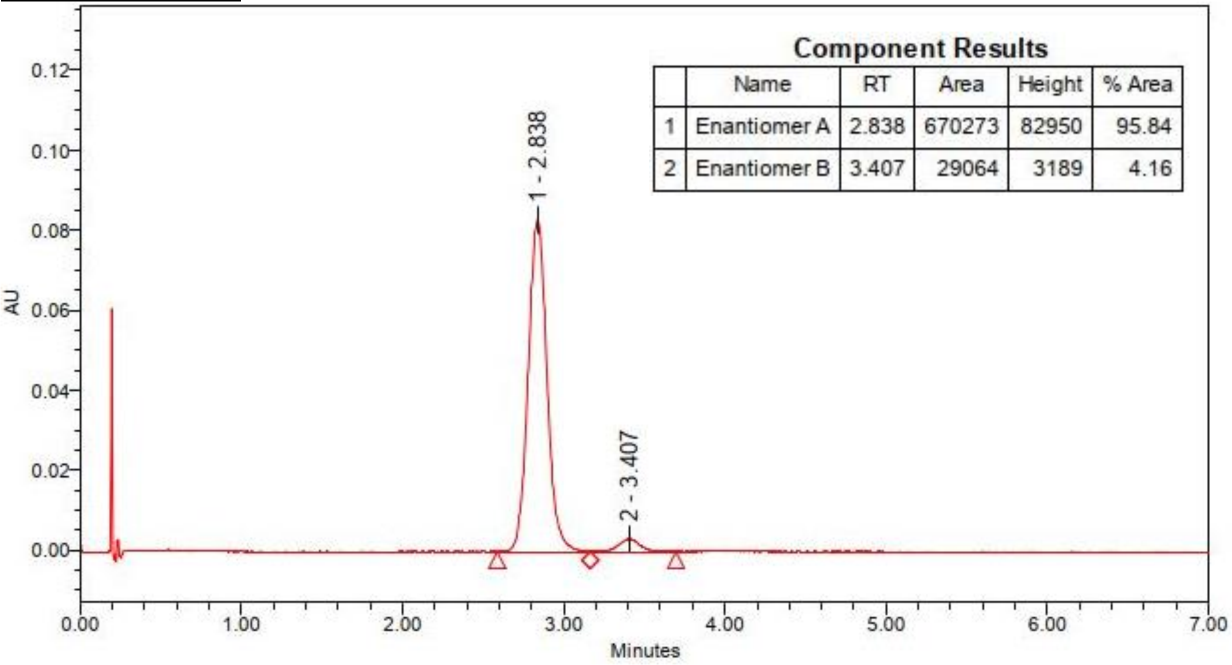

9a

Column: CHIRALPAK IB-3 SFC (3.0 mm × 100 mm). Eluent: CO_2:IPA = 70:30

<u>Racemic-</u> 9a

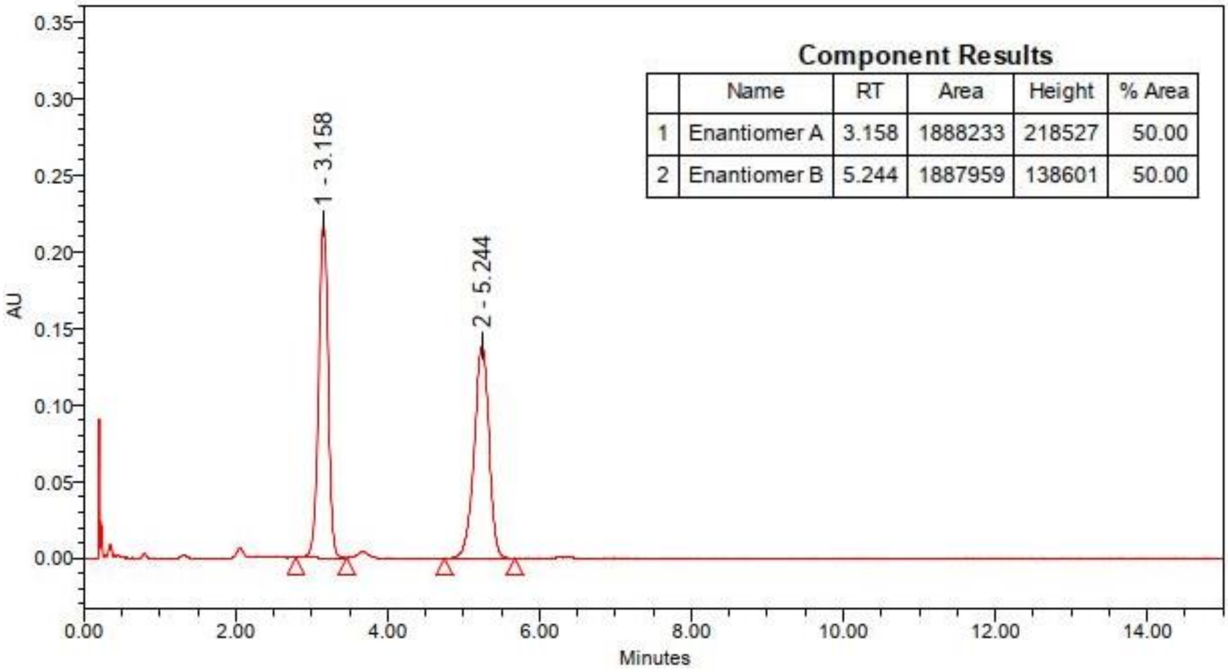

	Name	RT	Area	Height	% Area
1	Enantiomer A	3.158	1888233	218527	50.00
2	Enantiomer B	5.244	1887959	138601	50.00

<u>Enantioenriched-</u> 9a 92% *ee*

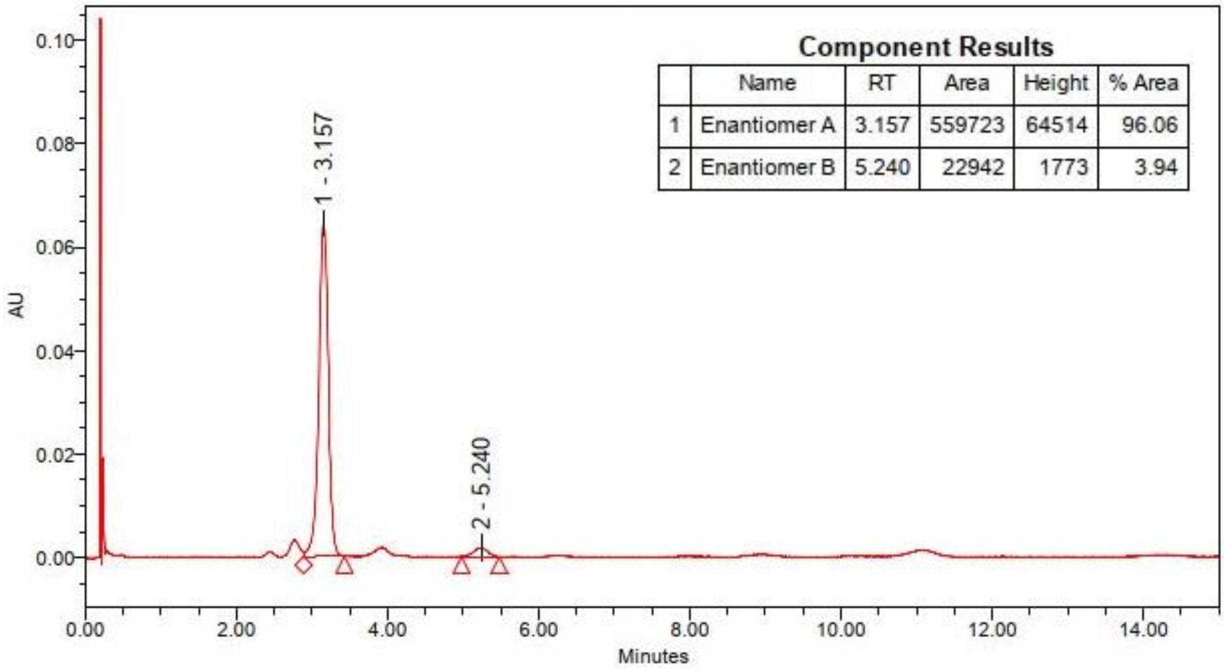

	Name	RT	Area	Height	% Area
1	Enantiomer A	3.157	559723	64514	96.06
2	Enantiomer B	5.240	22942	1773	3.94

11a

Column: CHIRALPAK IA-3 SFC (3.0 mm × 100 mm). Eluent: CO_2:IPA = 70:30

<u>Racemic-</u> 11a

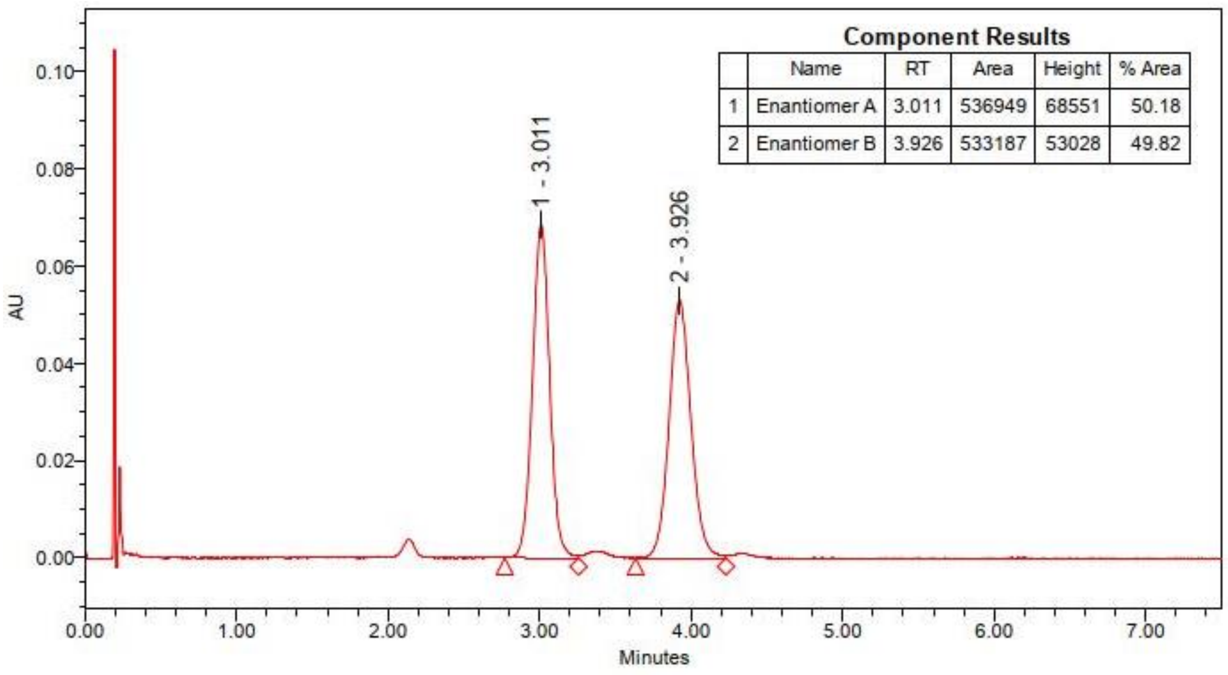

Component Results

	Name	RT	Area	Height	% Area
1	Enantiomer A	3.011	536949	68551	50.18
2	Enantiomer B	3.926	533187	53028	49.82

<u>Enantioenriched-</u> 11a 91% *ee*

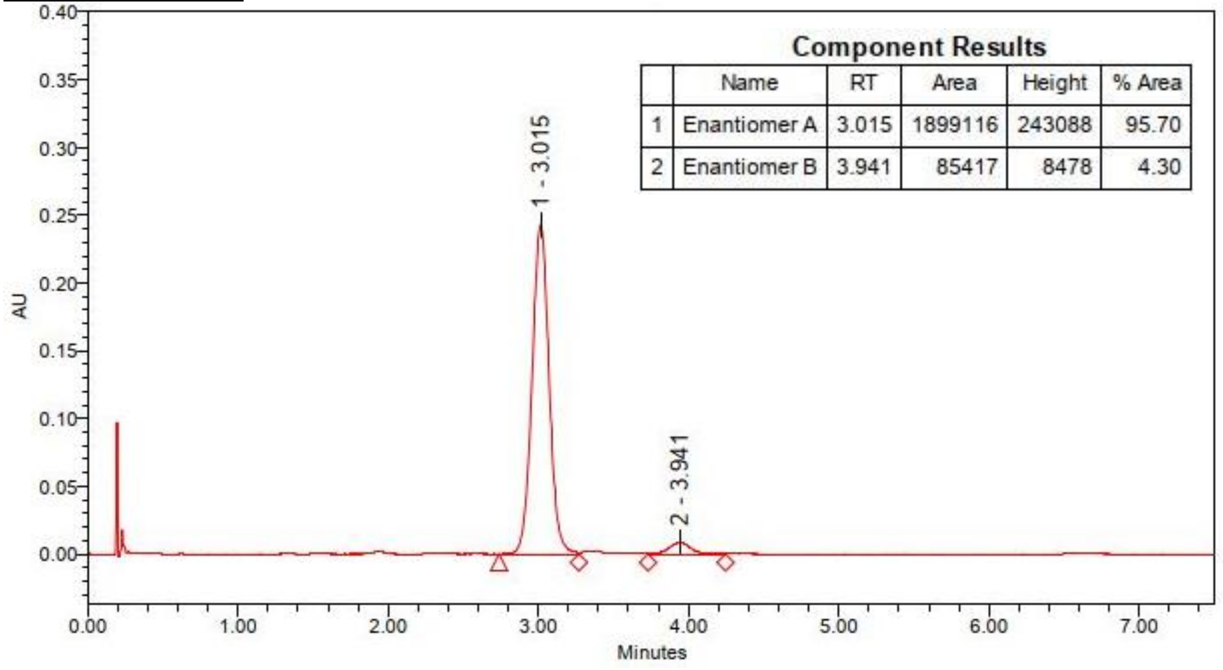

Component Results

	Name	RT	Area	Height	% Area
1	Enantiomer A	3.015	1899116	243088	95.70
2	Enantiomer B	3.941	85417	8478	4.30

10a

Column: CHIRALPAK IA-3 SFC (3.0 mm × 100 mm). Eluent: CO_2:IPA = 80:20

<u>Racemic-</u> 10a

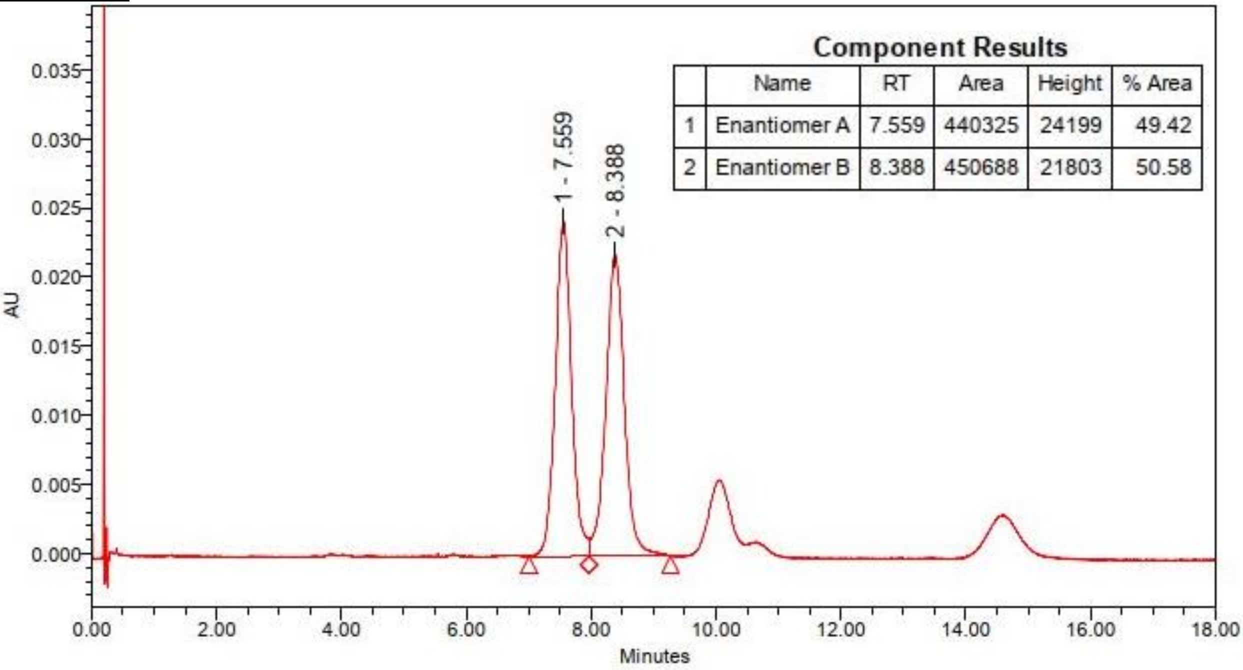

	Name	RT	Area	Height	% Area
1	Enantiomer A	7.559	440325	24199	49.42
2	Enantiomer B	8.388	450688	21803	50.58

<u>Enantioenriched-</u> 10a 90% *ee*

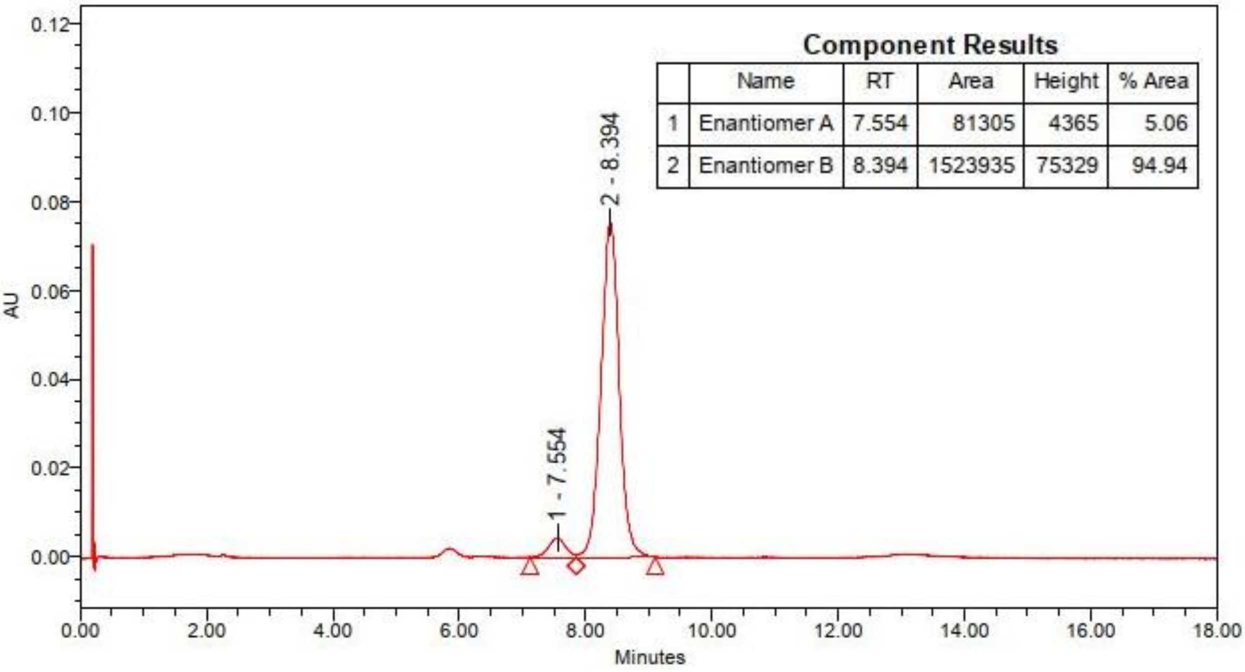

	Name	RT	Area	Height	% Area
1	Enantiomer A	7.554	81305	4365	5.06
2	Enantiomer B	8.394	1523935	75329	94.94

Chiroptical Properties

Circular Dichroism

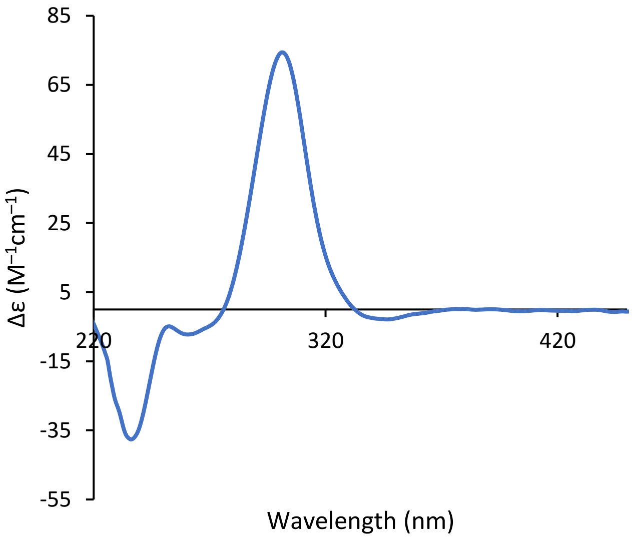

*Figure S 1: Circular dichroism spectrum (10 µM in CH_2Cl_2) of **1a**.*

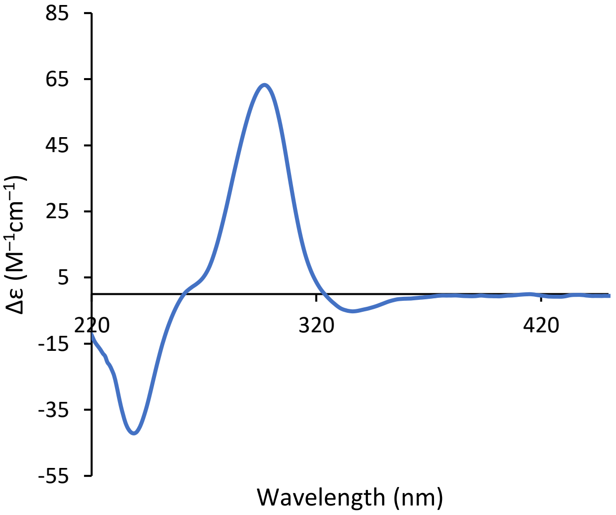

*Figure S 2: Circular dichroism spectrum (10 µM in CH_2Cl_2) of **1b**.*

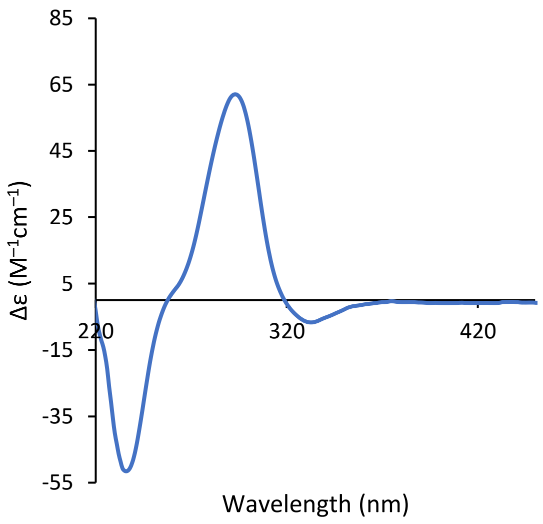

*Figure S 3: Circular dichroism spectrum (10 µM in CH_2Cl_2) of **1c**.*

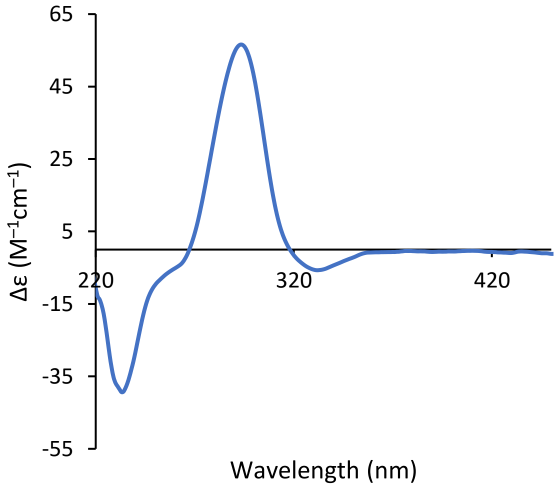

*Figure S 4: Circular dichroism spectrum (10 µM in CH_2Cl_2) of **1d**.*

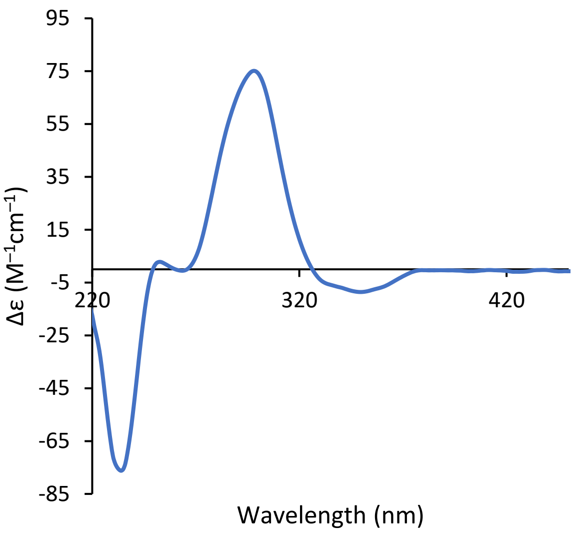

*Figure S 5: Circular dichroism spectrum (10 µM in CH_2Cl_2) of **1e**.*

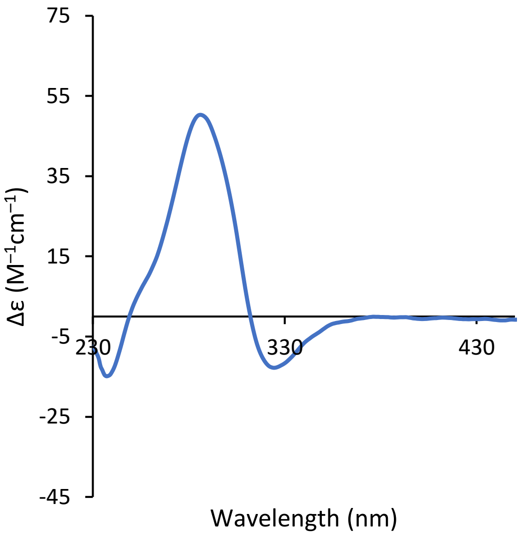

*Figure S 6: Circular dichroism spectrum (10 µM in CH_2Cl_2) of **1f**.*

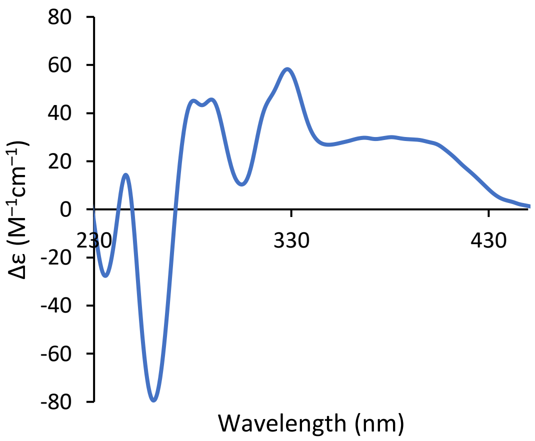

*Figure S 7: Circular dichroism spectrum (10 µM in CH_2Cl_2) of **8a**.*

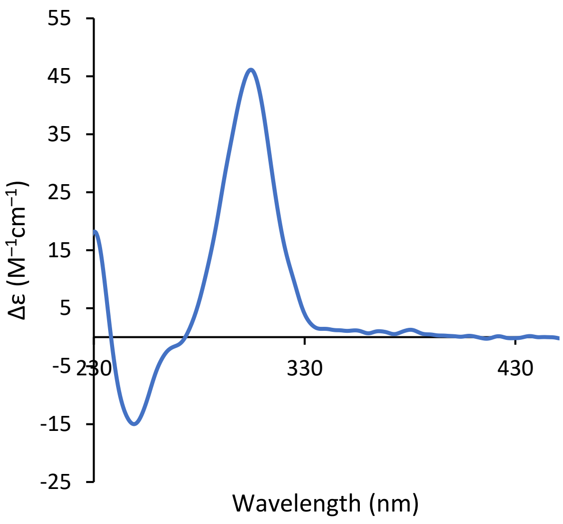

*Figure S 8: Circular dichroism spectrum (10 µM in CH_2Cl_2) of **9a**.*

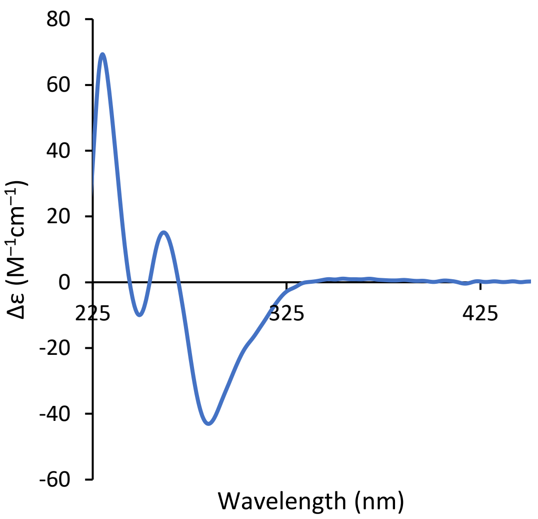

*Figure S 9: Circular dichroism spectrum (10 µM in CH_2Cl_2) of **11a**.*

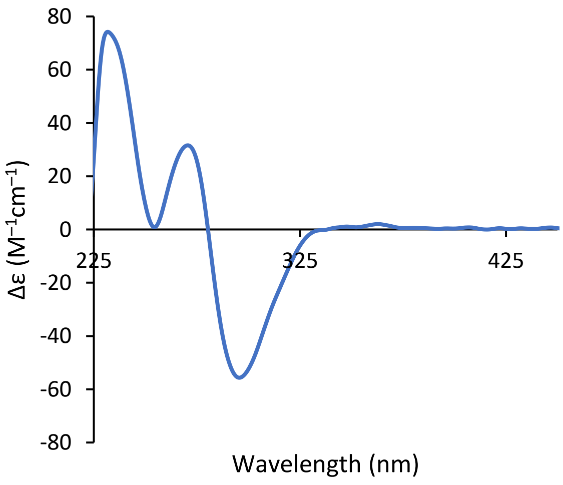

*Figure S 10: Circular dichroism spectrum (10 µM in CH_2Cl_2) of **10a**.*

Circular polarized luminescence spectra

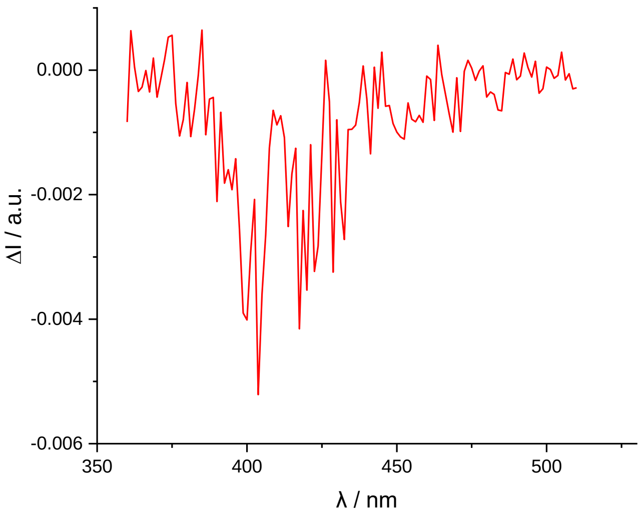

*Figure S 11: CPL spectrum of **1a** (1.0 × 10^{-5} M in CH_2Cl_2).*

X-RAY CRYSTALLOGRAPHIC ANALYSIS

General

Data collection was done on two dual source equiv.uipped *Bruker D8 Venture* four-circle-diffractometer from *Bruker AXS GmbH*; used X-ray sources: microfocus *IµS 2.0* Cu/Mo and microfocus *IµS 3.0* Ag/Mo from *Incoatec GmbH* with mirror optics *HELIOS* and single-hole collimator from *Bruker AXS GmbH;* used detector: *Photon III CE14* (Cu/Mo) and *Photon III HE* (Ag/Mo) from *Bruker AXS GmbH*.

Used programs: *APEX4 Suite* (v2022.1-1) for data collection and therein integrated programs *SAINT* V8.40A (Integration) und *SADABS* 2016/2 (Absorption correction) from *Bruker AXS GmbH*; structure solution was done with *SHELXT*[5], refinement with *SHELXL*-2018/3[5]; OLEX^2 and FinalCif were used for data finalization.

Special Utilities: *SMZ1270* stereomicroscope from *Nikon Metrology GmbH* was used for sample preparation; crystals were mounted on *MicroMounts* or *MicroLoops* from *MiTeGen* in NVH oil; crystals were cooled to given temperature with *Cryostream 800* from *Oxford Cryosystems.*

This supplement contains in the following the refinement detail tables, a figure of the complete asymmetric unit and a picture of the crystal used for data collection. Further details can be obtained from the crystallographic information files (CIFs) uploaded to the *Cambridge Crystallographic Data Centre* (CCDC), where they can be obtained free of charge.

Identifier	CCDC number
**(P)-1e**	2479174
**rac-1e**	2479175
**1f**	2479176
**S2d**	2479180
**3d**	2479173
**5a**	2479177
**9a**	2479178
**10a**	2479179

Refinement details

Compound (P)-1e*◦*DCM

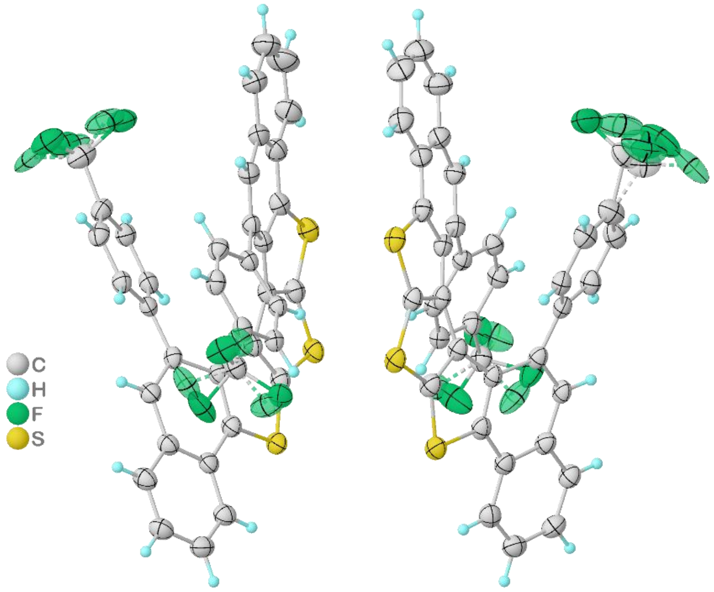

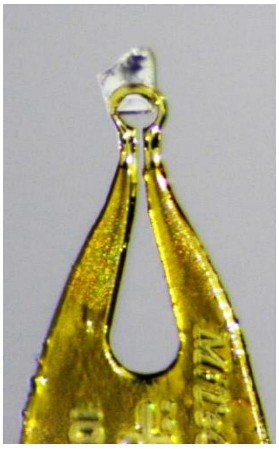

*Figure S 12: Full asymmetric unit of (P)-**1e**◦**0.5** DCM. The severely disordered DCM molecule was omitted for clarity. Displacement ellipsoids are drawn at 50% probability level. Non-merohedral twinning was found with a second domain related by (1 0 -0.005 / 0 -1 0 / -0.356 0 -1) and a refined batch scale factor of 0. 3651(13). The crystals were obtained from a solution of a mixture of dichloromethane and hexane.*

CCDC number	2479174
Empirical formula	$C_{77}H_{38}Cl_2F_{12}S_6$
Formula weight	1454.33
Temperature [K]	100.00
Crystal system	Monoclinic
Space group (number)	$P2_1$ (4)
a [Å]	16.6491(5)
b [Å]	7.9062(2)
c [Å]	24.9766(8)
α [°]	90
β [°]	97.027(2)
γ [°]	90
Volume [Å^3]	3263.00(17)
Z	2
ρ_{calc} [gcm^{-3}]	1.480
μ [mm^{-1}]	3.385
F(000)	1476
Crystal size [mm^3]	0.189×0.157×0.033
Crystal color	Colorless
Crystal shape	Plate
Radiation	CuK_α (λ=1.54178 Å)
2θ range [°]	5.35 to 157.56 (0.79 Å)

Index ranges	$-21 \le h \le 21$<br>$-9 \le k \le 9$<br>$-31 \le l \le 31$
Reflections collected	13383
Independent reflections	13383<br>R_{int} = 0.0637<br>R_{sigma} = 0.0368
Completeness to θ = 67.679°	99.8 %
Data / Restraints / Parameters	13383/357/1119
Absorption correction T_{min}/T_{max} (method)	0.603796/0.754205 (multi-scan)
Goodness-of-fit on F^2	1.032
Final *R* indexes [$I \geq 2\sigma(I)$]	R_1 = 0.0384<br>wR_2 = 0.0994
Final *R* indexes [all data]	R_1 = 0.0410<br>wR_2 = 0.1022
Largest peak/hole [eÅ^{-3}]	0.21/−0.20
Flack X parameter	0.008(9)

Compound rac-1e

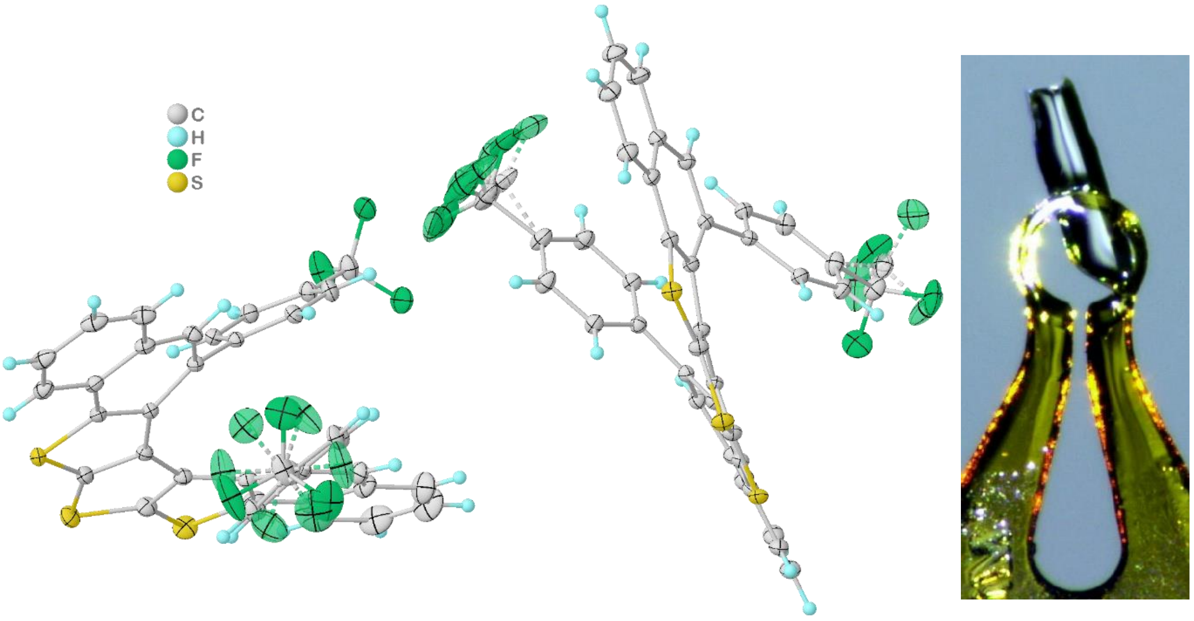

*Figure S 13: Full asymmetric unit (Z'=2) of rac-**1e**. Displacement ellipsoids are drawn at 50% probability level; the minor disorder part is drawn translucent with stippled bonds. The crystals were obtained from a solution of a mixture of ethyl acetate and hexane.*

CCDC number	2479175
Empirical formula	$C_{38}H_{18}F_6S_3$
Formula weight	684.70
Temperature [K]	100.00
Crystal system	Triclinic
Space group (number)	$P\bar{1}$ (2)
a [Å]	13.2828(15)
b [Å]	14.2038(9)
c [Å]	18.067(2)
α [°]	67.511(2)
β [°]	73.705(2)
γ [°]	86.208(3)
Volume [Å^3]	3019.7(5)
Z	4
ρ_{calc} [gcm^{-3}]	1.506
μ [mm^{-1}]	0.312
F(000)	1392
Crystal size [mm^3]	0.596×0.117×0.093
Crystal color	Yellow
Crystal shape	Plank
Radiation	MoK_α (λ=0.71073 Å)
2θ range [°]	4.09 to 65.13 (0.66 Å)

Index ranges	−20 ≤ h ≤ 20<br>−21 ≤ k ≤ 17<br>−27 ≤ l ≤ 27
Reflections collected	231245
Independent reflections	21939<br>R_{int} = 0.0366<br>R_{sigma} = 0.0178
Completeness to θ = 67.679°	100.0 %
Data / Restraints / Parameters	21939/195/1016
Absorption correction T_{min}/T_{max} (method)	0.9122/0.9943 (numerical)
Goodness-of-fit on F^2	1.033
Final *R* indexes [*I*≥2σ(*I*)]	R_1 = 0.0347<br>wR_2 = 0.0886
Final *R* indexes [all data]	R_1 = 0.0435<br>wR_2 = 0.0953
Largest peak/hole [eÅ^{-3}]	0.51/−0.53

Compound rac-1f

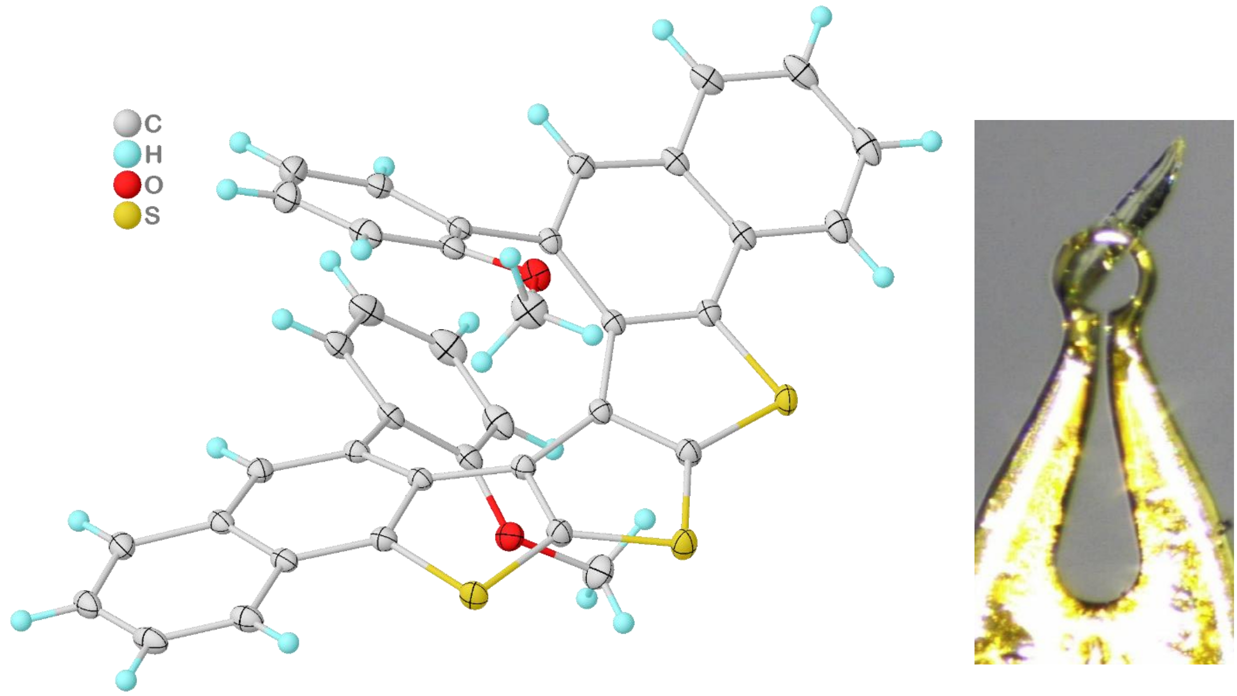

*Figure S 14: Full asymmetric unit (Z'=0.5) of rac-**1f**.* Other half of the symmetric molecule generated by symmetry code 1-X, -+Y, ½+Z. *Displacement ellipsoids are drawn at 50% probability level. The crystals were obtained from a solution of a mixture of acetonitrile and water.*

CCDC number	2479176
Empirical formula	$C_{38}H_{24}O_2S_3$
Formula weight	608.75
Temperature [K]	100.00
Crystal system	Monoclinic
Space group (number)	$C2/c$ (15)
a [Å]	16.2789(18)
b [Å]	15.3827(16)
c [Å]	11.4782(11)
α [°]	90
β [°]	101.692(4)
γ [°]	90
Volume [Å^3]	2814.7(5)
Z	4
ρ_{calc} [gcm^{-3}]	1.437
μ [mm^{-1}]	0.300
F(000)	1264
Crystal size [mm^3]	0.501×0.087×0.064
Crystal color	Colorless
Crystal shape	Needle
Radiation	MoK_α (λ=0.71073 Å)
2θ range [°]	4.79 to 56.72 (0.75 Å)

Index ranges	−21 ≤ h ≤ 21<br>−20 ≤ k ≤ 20<br>−15 ≤ l ≤ 15
Reflections collected	33378
Independent reflections	3504<br>R_{int} = 0.0413<br>R_{sigma} = 0.0197
Completeness to θ = 67.679°	100.0 %
Data / Restraints / Parameters	3504/0/196
Absorption correction T_{min}/T_{max} (method)	0.7922/1.0000 (numerical)
Goodness-of-fit on F^2	1.045
Final *R* indexes [I≥2σ(I)]	R_1 = 0.0332<br>wR_2 = 0.0836
Final *R* indexes [all data]	R_1 = 0.0378<br>wR_2 = 0.0873
Largest peak/hole [eÅ^{-3}]	0.42/−0.25

Compound S2d

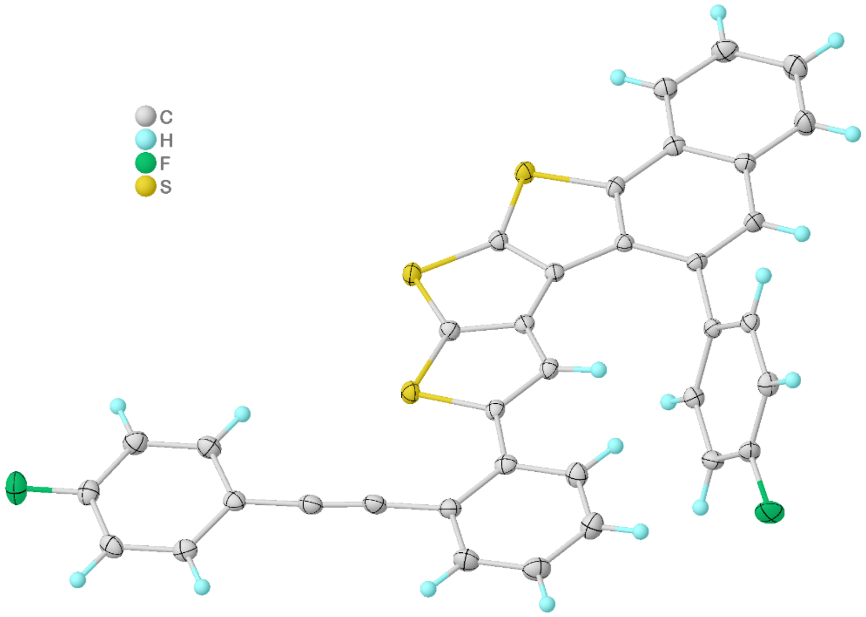

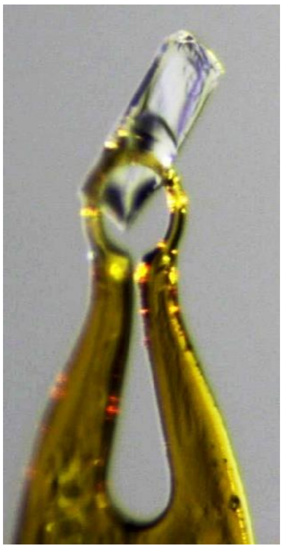

Figure S 15: Full asymmetric unit of ***S2d****. Displacement ellipsoids are drawn at 50% probability level. Non-merohedral twinning was found with a second domain related by (1 0 -0.003 / 0 -1 0 / -0.162 0 -1) and a refined batch scale factor of 0.3234(8). The crystals were obtained from ethyl acetate and hexane directly after collection by column chromatography.*

CCDC number	2479180
Empirical formula	$C_{36}H_{18}F_2S_3$
Formula weight	584.68
Temperature [K]	100.00
Crystal system	Monoclinic
Space group (number)	$P2_1/n$ (14)
a [Å]	6.1063(10)
b [Å]	17.360(3)
c [Å]	24.441(4)
α [°]	90
β [°]	90.808(4)
γ [°]	90
Volume [Å^3]	2590.7(8)
Z	4
ρ_{calc} [gcm^{-3}]	1.499
μ [mm^{-1}]	0.328
F(000)	1200
Crystal size [mm^3]	0.475×0.179×0.136
Crystal color	Colorless
Crystal shape	plate
Radiation	MoK_α (λ=0.71073 Å)
2θ range [°]	4.08 to 61.05 (0.70 Å)

Index ranges	$-8 \le h \le 8$<br>$0 \le k \le 24$<br>$0 \le l \le 34$
Reflections collected	7315
Independent reflections	7315<br>R_{int} = 0.0582<br>R_{sigma} = 0.0381
Completeness to θ = 67.679°	99.6 %
Data / Restraints / Parameters	7315/0/371
Absorption correction T_{min}/T_{max} (method)	0.499766/0.621358 (multi-scan)
Goodness-of-fit on F^2	1.060
Final *R* indexes [$I \ge 2\sigma(I)$]	R_1 = 0.0379<br>wR_2 = 0.0916
Final *R* indexes [all data]	R_1 = 0.0464<br>wR_2 = 0.0979
Largest peak/hole [eÅ^{-3}]	0.38/−0.30

Compound 3d

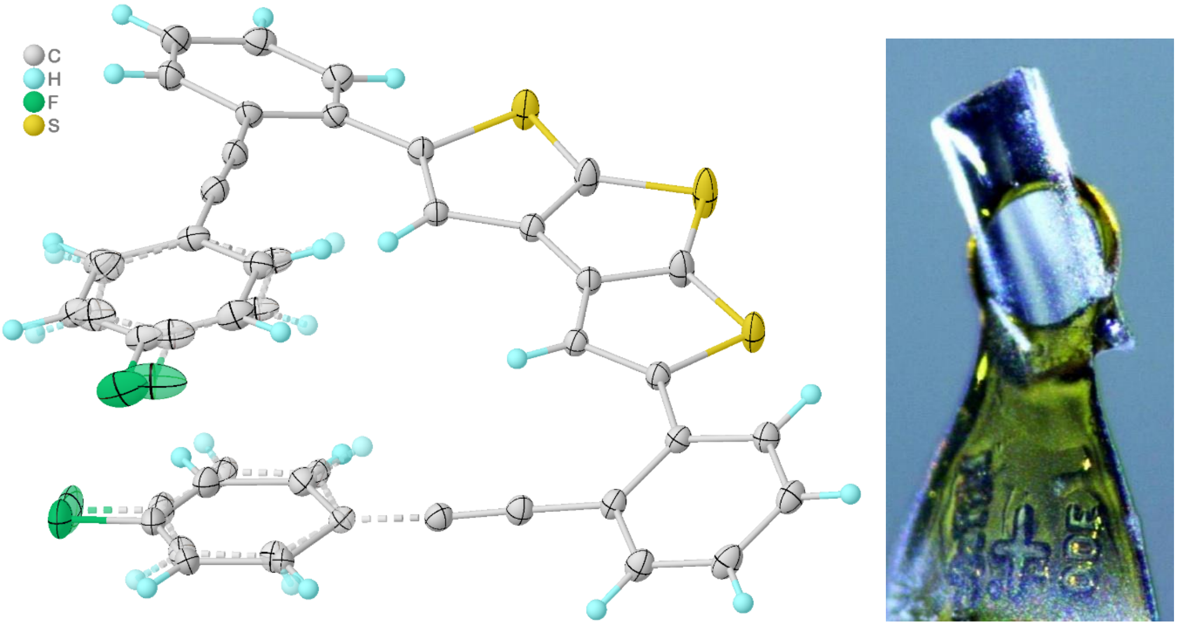

*Figure S 16: Full asymmetric unit (Z'=0.5) of **3d**. Other half of the symmetric molecule generated by symmetry code ½-X, ½-Y,+Z. Displacement ellipsoids are drawn at 50% probability level; the minor disorder part is drawn translucent with stippled bonds. The crystals were obtained from a solution of a mixture of ethyl acetate and hexane.*

CCDC number	2479173
Empirical formula	$C_{36}H_{18}F_2S_3$
Formula weight	584.68
Temperature [K]	100.00
Crystal system	Orthorhombic
Space group (number)	$Fdd2$ (43)
a [Å]	32.560(5)
b [Å]	6.5554(6)
c [Å]	25.584(5)
α [°]	90
β [°]	90
γ [°]	90
Volume [Å^3]	5460.7(14)
Z	8
ρ_{calc} [gcm^{-3}]	1.422
μ [mm^{-1}]	0.311
F(000)	2400
Crystal size [mm^3]	0.629×0.302×0.044
Crystal color	colorless
Crystal shape	plate
Radiation	MoK_α (λ=0.71073 Å)

2θ range [°]	4.05 to 60.96 (0.70 Å)
Index ranges	−46 ≤ h ≤ 46<br>−9 ≤ k ≤ 9<br>−36 ≤ l ≤ 36
Reflections collected	60382
Independent reflections	4165<br>R_{int} = 0.0301<br>R_{sigma} = 0.0119
Completeness to θ = 25.242°	100.0 %
Data / Restraints / Parameters	4165/105/241
Goodness-of-fit on F^2	1.096
Final R indexes [I≥2σ(I)]	R_1 = 0.0214<br>wR_2 = 0.0561
Final R indexes [all data]	R_1 = 0.0220<br>wR_2 = 0.0567
Largest peak/hole [eÅ^{-3}]	0.25/-0.23
Flack X parameter	0.031(11)

Compound 5a◦2 $HCCl_3$

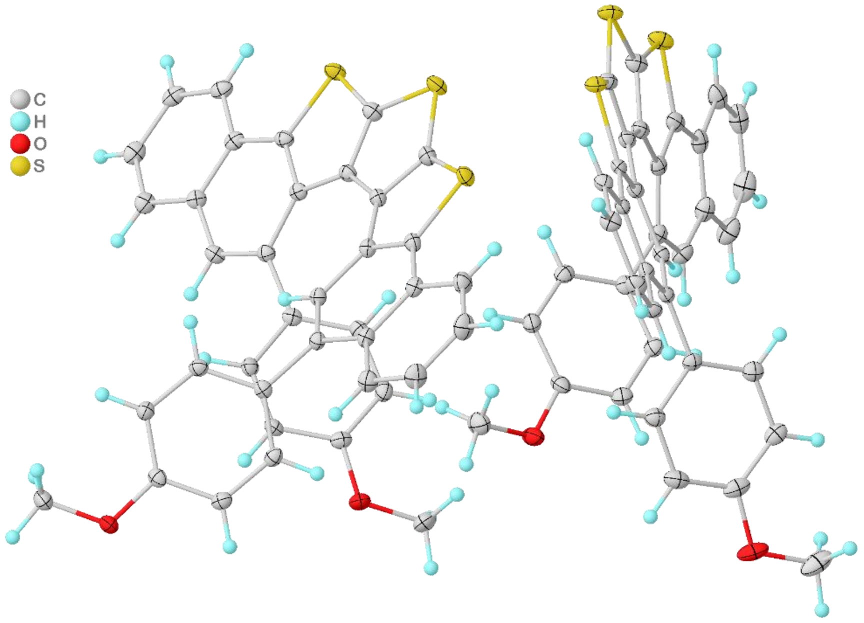

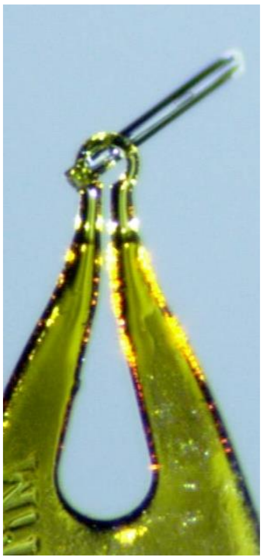

Figure S 17: Full asymmetric unit (Z'=2) of ***5a****◦2 $HCCl_3$. The severely disordered chloroform molecules were omitted for clarity. Displacement ellipsoids are drawn at 50% probability level. The crystals were obtained from chloroform.*

CCDC number	2479177
Empirical formula	$C_{40}H_{26}Cl_6O_2S_3$
Formula weight	847.49
Temperature [K]	100.00
Crystal system	Monoclinic
Space group (number)	$P2_1/n$ (14)
a [Å]	13.8640(18)
b [Å]	21.852(2)
c [Å]	24.844(3)
α [°]	90
β [°]	103.597(4)
γ [°]	90
Volume [Å^3]	7315.8(16)
Z	8
ρ_{calc} [gcm^{-3}]	1.539
μ [mm^{-1}]	0.679
F(000)	3456
Crystal size [mm^3]	0.509×0.047×0.03
Crystal color	Colorless
Crystal shape	Needle
Radiation	MoK_α (λ=0.71073 Å)
2θ range [°]	3.73 to 56.60 (0.75 Å)

Index ranges	$-18 \leq h \leq 18$<br>$-29 \leq k \leq 29$<br>$-33 \leq l \leq 33$
Reflections collected	366677
Independent reflections	18179<br>R_{int} = 0.0706<br>R_{sigma} = 0.0270
Completeness to θ = 67.679°	100.0 %
Data / Restraints / Parameters	18179/136/1060
Absorption correction T_{min}/T_{max} (method)	0.8528/0.9949 (numerical)
Goodness-of-fit on F^2	1.049
Final *R* indexes [$I \geq 2\sigma(I)$]	R_1 = 0.0438<br>wR_2 = 0.0973
Final *R* indexes [all data]	R_1 = 0.0604<br>wR_2 = 0.1063
Largest peak/hole [eÅ^{-3}]	0.88/−0.57

Compound rac-9a

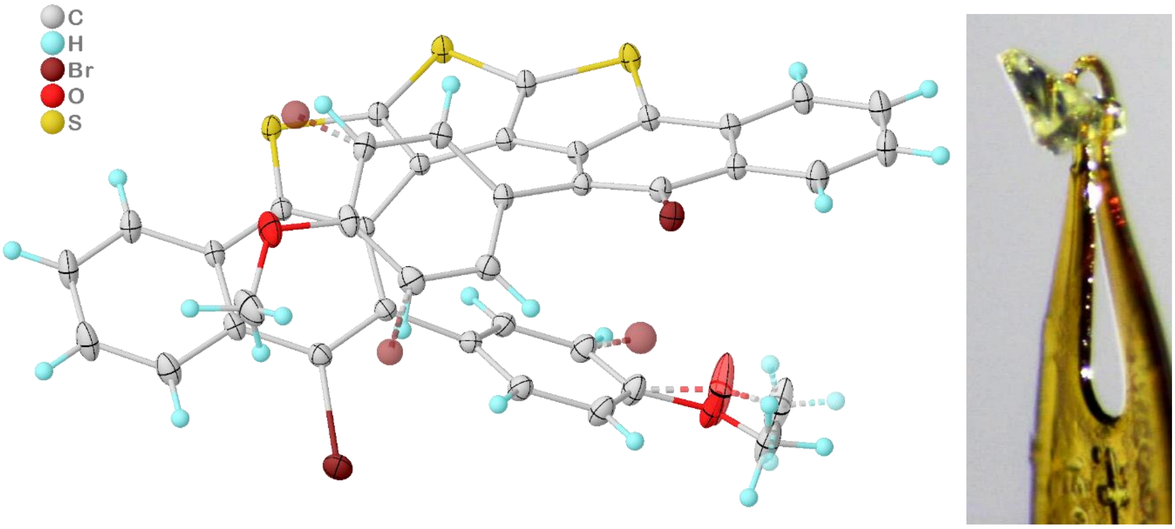

*Figure S 18: Full asymmetric unit of rac-**9a**. Displacement ellipsoids are drawn at 50% probability level; the minor disorder part is drawn translucent with stippled bonds. The three alternative bromine positions were refined with occupancy factor between 0.01 and 0.02 and are likely the result from minor impurities by regiosiomers. The crystals were obtained from a solution of a mixture of ethyl acetate and hexane.*

CCDC number	2479178
Empirical formula	$C_{38}H_{22}Br_2O_2S_3$
Formula weight	766.55
Temperature [K]	100.00
Crystal system	Monoclinic
Space group (number)	$P2_1/n$ (14)
a [Å]	8.9156(4)
b [Å]	25.9710(14)
c [Å]	13.2783(7)
α [°]	90
β [°]	100.9780(10)
γ [°]	90
Volume [Å^3]	3018.3(3)
Z	4
ρ_{calc} [gcm^{-3}]	1.687
μ [mm^{-1}]	2.930
F(000)	1536
Crystal size [mm^3]	0.301×0.194×0.165
Crystal color	Colorless
Crystal shape	Block
Radiation	MoK_α (λ=0.71073 Å)
2θ range [°]	4.43 to 65.25 (0.66 Å)

Index ranges	−10 ≤ h ≤ 13<br>−39 ≤ k ≤ 38<br>−20 ≤ l ≤ 20
Reflections collected	105396
Independent reflections	10903<br>R_{int} = 0.0457<br>R_{sigma} = 0.0222
Completeness to θ = 67.679°	100.0 %
Data / Restraints / Parameters	10903/21/443
Absorption correction T_{min}/T_{max} (method)	0.5429/0.8647 (numerical)
Goodness-of-fit on F^2	1.027
Final *R* indexes [$I \geq 2\sigma(I)$]	R_1 = 0.0293<br>wR_2 = 0.0735
Final *R* indexes [all data]	R_1 = 0.0350<br>wR_2 = 0.0762
Largest peak/hole [eÅ^{-3}]	1.40/−0.79

Compound rac-10a

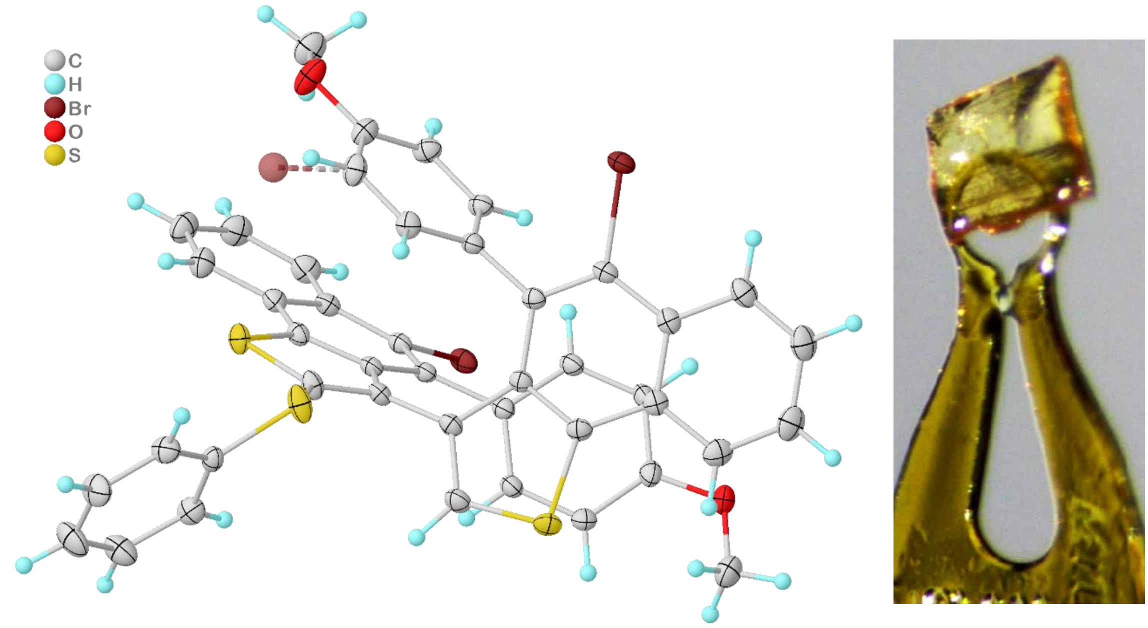

*Figure S 19: Full asymmetric unit of rac-**10a**. Displacement ellipsoids are drawn at 50% probability level; the minor disorder part is drawn translucent with stippled bonds. The alternative bromine position was refined with occupancy factor between 0.0244(11) and is likely the result from minor impurities by regiosiomers. The crystals were obtained from a solution of a mixture of dichloromethane and hexane.*

CCDC number	2479179
Empirical formula	$C_{44}H_{28}Br_{2.03}O_2S_3$
Formula weight	846.66
Temperature [K]	100.00
Crystal system	Triclinic
Space group (number)	$P\bar{1}$ (2)
a [Å]	11.8299(7)
b [Å]	12.3531(10)
c [Å]	13.5200(11)
α [°]	101.759(2)
β [°]	107.913(2)
γ [°]	105.919(2)
Volume [Å^3]	1715.9(2)
Z	2
ρ_{calc} [gcm^{-3}]	1.639
μ [mm^{-1}]	2.615
F(000)	854
Crystal size [mm^3]	0.362×0.284×0.068
Crystal color	Orange
Crystal shape	Plate
Radiation	MoK_α (λ=0.71073 Å)
2θ range [°]	3.88 to 59.36 (0.72 Å)

Index ranges	−16 ≤ h ≤ 13<br>−17 ≤ k ≤ 17<br>−18 ≤ l ≤ 18
Reflections collected	91379
Independent reflections	9693<br>R_{int} = 0.0431<br>R_{sigma} = 0.0221
Completeness to θ = 67.679°	100.0 %
Data / Restraints / Parameters	9693/0/468
Absorption correction T_{min}/T_{max} (method)	0.4538/0.9828 (numerical)
Goodness-of-fit on F^2	1.025
Final *R* indexes [$I \geq 2\sigma(I)$]	R_1 = 0.0287<br>wR_2 = 0.0718
Final *R* indexes [all data]	R_1 = 0.0346<br>wR_2 = 0.0753
Largest peak/hole [eÅ^{-3}]	1.22/−0.47

Computational Studies

Calculations were carried out with Gaussian 16, Rev. A.03, and output files were analyzed with GaussView 6.0.19.[5] Absolute electronic energies for calculation of ΔH, ΔG and ΔS are listed in the table below:

	Electronic Energy (EE) [Hartree]	EE + Thermal Enthalpy Correction [Hartree]	EE + Thermal Free Energy Correction [Hartree]	Entropy [cal/(mol K)]
**1c** Minimum	-2579.241618	-2578.767996	-2578.856418	186.098
**1c** TS	-2579.180967	-2578.709713	-2578.796844	183.381
**6c** Minimum	-2258.499033	-2257.992555	-2258.081443	187.082
**6** TS	-2258.422199	-2257.917988	-2258.005117	183.378

Optimized geometry of minima and transition states (cartesian coordinates) as well as used Gaussian command lines (following #) are supplied.

1c Minimum

opt=tight freq b3lyp def2tzvp empiricaldispersion=gd3bj int=grid=superfinegrid

S	2.93788600	2.84224800	0.04579600
S	-0.00000200	3.98933700	-0.00000600
S	-2.93789000	2.84224600	-0.04579500
C	3.02974300	1.10469300	-0.17867600
C	4.24114300	0.37227500	-0.21939400
C	5.51041900	0.92510900	0.05172600
H	5.59080000	1.97518800	0.30431200
C	6.63689000	0.14090300	0.01943500
H	7.60330500	0.57579800	0.23847900
C	6.54170600	-1.22823100	-0.29474700
H	7.43587800	-1.83728900	-0.31746500
C	5.31976300	-1.78649200	-0.57557700
H	5.24252000	-2.83700200	-0.82812400
C	4.13996500	-1.01038700	-0.54593400
C	2.88162100	-1.54730300	-0.91735200
H	2.86795000	-2.54623900	-1.33231300
C	1.71412100	-0.82588100	-0.87374200
C	0.50144500	-1.36348700	-1.53366300
C	-0.34079800	-0.52751700	-2.27267000
H	-0.11569500	0.52575000	-2.34735400
C	-1.46135600	-1.02833500	-2.92039900
H	-2.10442500	-0.35433600	-3.47116700
C	-1.75800900	-2.38266400	-2.85946400
C	-0.90509700	-3.23503700	-2.16598500
H	-1.11794400	-4.29569000	-2.12023600

C	0.21103300	-2.73156700	-1.51776600
H	0.84916600	-3.40032100	-0.95890100
C	1.77322500	0.51045800	-0.34965900
C	0.71628000	1.45913900	-0.08217900
C	-0.71628300	1.45913900	0.08217700
C	-1.77322700	0.51045800	0.34966000
C	-1.71412100	-0.82588100	0.87374400
C	-0.50144300	-1.36348700	1.53366100
C	0.34080100	-0.52751700	2.27266900
H	0.11569700	0.52574900	2.34735700
C	1.46136200	-1.02833500	2.92039300
H	2.10443000	-0.35433700	3.47116200
C	1.75801700	-2.38266400	2.85945400
C	0.90510500	-3.23503600	2.16597400
H	1.11795400	-4.29568900	2.12022100
C	-0.21102700	-2.73156600	1.51776000
H	-0.84916100	-3.40032000	0.95889400
C	-2.88162000	-1.54730400	0.91735600
H	-2.86794800	-2.54624000	1.33231700
C	-4.13996500	-1.01038900	0.54594000
C	-4.24114500	0.37227200	0.21940000
C	-3.02974600	1.10469100	0.17867800
C	-5.51042200	0.92510500	-0.05171900
H	-5.59080400	1.97518400	-0.30430600
C	-6.63689200	0.14089800	-0.01942500
H	-7.60330800	0.57579200	-0.23846900
C	-6.54170700	-1.22823500	0.29475600
H	-7.43587800	-1.83729400	0.31747600
C	-5.31976200	-1.78649500	0.57558500
H	-5.24251800	-2.83700500	0.82813300
C	-1.21469600	2.75031800	-0.00067600
C	1.21469200	2.75031800	0.00067300
H	-2.63940900	-2.77239900	-3.35149500
H	2.63941900	-2.77239900	3.35148100

1c TS

opt=(calcall,tight,ts,maxstep=6,maxcycle=900,noeigen) freq b3lyp def2tzvp empiricaldispersion=gd3bj int=grid=superfinegrid

S	2.79283100	-2.65702700	1.36330300
S	-0.00000100	-3.07009000	2.51004200
S	-2.79283300	-2.65702500	1.36330400
C	2.92375100	-1.41247700	0.15153500
C	4.00443800	-1.35214700	-0.76570200
C	5.03270400	-2.31414500	-0.83902300
H	5.04540000	-3.13918800	-0.13769200
C	6.00174700	-2.22779100	-1.80874300
H	6.78071500	-2.97707900	-1.86043700

C	5.98387700	-1.17300000	-2.74069400
H	6.75117300	-1.11630900	-3.50166200
C	5.00132600	-0.21609000	-2.67955400
H	4.99284300	0.60783200	-3.38253800
C	3.99195200	-0.27791100	-1.69443400
C	3.06170400	0.76933100	-1.48963100
H	3.26251400	1.70646200	-1.98956800
C	2.07564600	0.73272800	-0.52952200
C	1.67988700	2.04666500	0.03034400
C	1.68087100	2.20449800	1.41942400
H	1.72590700	1.32952500	2.05297000
C	1.69485000	3.46780700	1.99179100
H	1.71318000	3.56443600	3.06977800
C	1.69775200	4.60045600	1.19077200
C	1.64511600	4.45711300	-0.19068800
H	1.60661600	5.33345900	-0.82500200
C	1.62667400	3.19713800	-0.76297900
H	1.58500500	3.10084100	-1.83993300
C	1.84530200	-0.51386800	0.16157200
C	0.73972100	-1.02749800	0.98059700
C	-0.73972100	-1.02749700	0.98059700
C	-1.84530200	-0.51386800	0.16157200
C	-2.07564500	0.73272800	-0.52952400
C	-1.67988500	2.04666500	0.03034200
C	-1.62666800	3.19713700	-0.76298300
H	-1.58499700	3.10083800	-1.83993600
C	-1.64511000	4.45711300	-0.19069300
H	-1.60660800	5.33345800	-0.82500800
C	-1.69775000	4.60045700	1.19076700
C	-1.69485200	3.46780900	1.99178700
H	-1.71318500	3.56443900	3.06977400
C	-1.68087300	2.20449900	1.41942200
H	-1.72591100	1.32952700	2.05296800
C	-3.06170300	0.76933100	-1.48963200
H	-3.26251200	1.70646200	-1.98957000
C	-3.99195100	-0.27791000	-1.69443400
C	-4.00443900	-1.35214500	-0.76570200
C	-2.92375200	-1.41247500	0.15153500
C	-5.03270700	-2.31414200	-0.83902200
H	-5.04540300	-3.13918400	-0.13768900
C	-6.00174900	-2.22778800	-1.80874200
H	-6.78071800	-2.97707500	-1.86043400
C	-5.98387700	-1.17299800	-2.74069400
H	-6.75117300	-1.11630700	-3.50166200
C	-5.00132500	-0.21608900	-2.67955500
H	-4.99284100	0.60783200	-3.38254000
C	-1.19939500	-2.11462800	1.72226300
C	1.19939400	-2.11462900	1.72226200
H	1.71671500	5.58600800	1.63700800
H	-1.71671300	5.58601000	1.63700100

6c Minimum

opt=tight freq b3lyp def2tzvp empiricaldispersion=gd3bj int=grid=superfinegrid

S	-3.09773000	2.67771200	0.10060300
S	3.09772500	2.67772400	-0.10065600
C	-2.95916400	0.97548900	-0.25481700
C	-1.63645800	0.56835200	-0.45139500
C	-1.40278300	-0.70551500	-1.07356100
C	-2.46678100	-1.55985500	-1.21934600
H	-2.31171700	-2.51199300	-1.70967000
C	-3.78647600	-1.22398500	-0.82971800
C	-4.85626600	-2.13849200	-0.94414700
H	-4.64595400	-3.14236000	-1.29245900
C	-6.13861800	-1.76960400	-0.62200300
H	-6.94732000	-2.48349900	-0.70920600
C	-6.40839600	-0.45989800	-0.18105200
H	-7.42190600	-0.17560900	0.06995500
C	-5.39149700	0.45562300	-0.06720700
H	-5.60341800	1.45866800	0.28146500
C	-4.06299700	0.09695900	-0.37756700
C	-0.10773500	-1.06025600	-1.69918000
C	0.61669500	-0.11098700	-2.42622900
H	0.23090500	0.89352200	-2.52237100
C	1.82771100	-0.43688900	-3.01731200
H	2.37852400	0.32096900	-3.55911900
C	2.33694300	-1.72523200	-2.90718900
C	1.60510100	-2.69133000	-2.22819300
H	1.98684900	-3.70065000	-2.14294400
C	0.39406800	-2.36229300	-1.63793600
H	-0.15755200	-3.11543800	-1.09413300
C	-0.70207100	1.62581300	-0.10649700
C	0.70206900	1.62582000	0.10646500
C	1.36376600	2.86354400	-0.03195700
C	0.68749500	4.08664000	-0.06119400
H	1.23576300	5.01635500	-0.12820100
C	-0.68750900	4.08663700	0.06110400
H	-1.23578300	5.01635000	0.12808900
C	-1.36377300	2.86353700	0.03189500
C	1.63646100	0.56837200	0.45138200
C	1.40278500	-0.70548500	1.07357100
C	0.10774100	-1.06019600	1.69921200
C	-0.61666100	-0.11089900	2.42625500
H	-0.23084700	0.89360300	2.52237400
C	-1.82767500	-0.43676600	3.01735800
H	-2.37846700	0.32111100	3.55916100
C	-2.33693600	-1.72510100	2.90726100
C	-1.60512400	-2.69122400	2.22827100
H	-1.98689600	-3.70053700	2.14304200
C	-0.39409000	-2.36222300	1.63799400

H	0.15750800	-3.11538700	1.09419600
C	2.46678000	-1.55982700	1.21936000
H	2.31171700	-2.51195800	1.70969800
C	3.78647500	-1.22396300	0.82972100
C	4.06299700	0.09697700	0.37755800
C	2.95916500	0.97550800	0.25479700
C	5.39149800	0.45563700	0.06719400
H	5.60341900	1.45868000	-0.28148500
C	6.40839500	-0.45988500	0.18104500
H	7.42190600	-0.17559800	-0.06996500
C	6.13861600	-1.76958600	0.62200600
H	6.94731600	-2.48348300	0.70921300
C	4.85626300	-2.13846900	0.94415600
H	4.64595000	-3.14233500	1.29247600
H	-3.29223500	-1.97446500	3.34993300
H	3.29224200	-1.97462300	-3.34984500

6c TS

opt=(calcall,tight,ts,maxstep=6,maxcycle=900,noeigen) freq b3lyp def2tzvp empiricaldispersion=gd3bj int=grid=superfinegrid

S	-2.91778800	-2.32315000	-1.75945800
S	2.91778800	-2.32315000	-1.75945800
C	-2.80193900	-1.38178100	-0.30641800
C	-1.70904600	-0.50918400	-0.30947000
C	-1.83924800	0.63376800	0.56752200
C	-2.65961500	0.50890100	1.66580900
H	-2.80093900	1.36230700	2.31334600
C	-3.53726900	-0.58707900	1.85664600
C	-4.36935000	-0.70965800	2.98951100
H	-4.24008800	-0.01361900	3.80922100
C	-5.33535700	-1.68449700	3.05062100
H	-5.96462100	-1.77034800	3.92690700
C	-5.51694600	-2.57011500	1.97256300
H	-6.28156500	-3.33378800	2.02714400
C	-4.72303000	-2.47357900	0.85477800
H	-4.85425200	-3.17076500	0.03676100
C	-3.71292600	-1.49443600	0.77702900
C	-1.58435000	2.01387200	0.08590500
C	-1.56272200	3.10709400	0.95932800
H	-1.46591100	2.94098300	2.02361600
C	-1.66226200	4.40307700	0.48628400
H	-1.64089200	5.22946700	1.18520900
C	-1.76821000	4.64838000	-0.87801800
C	-1.75133400	3.57837300	-1.76013300
H	-1.81811000	3.75141600	-2.82661200
C	-1.66055900	2.27805200	-1.28513500
H	-1.68973500	1.45564400	-1.98518200
C	-0.72280400	-0.90354700	-1.32821600

C	0.72280400	-0.90354700	-1.32821600
C	1.33990700	-1.78251800	-2.25489600
C	0.68531400	-2.40137300	-3.32201300
H	1.24164100	-2.98595500	-4.04219000
C	-0.68531400	-2.40137300	-3.32201300
H	-1.24164100	-2.98595500	-4.04219000
C	-1.33990700	-1.78251800	-2.25489600
C	1.70904600	-0.50918400	-0.30947000
C	1.83924800	0.63376800	0.56752200
C	1.58435000	2.01387200	0.08590500
C	1.66055900	2.27805200	-1.28513500
H	1.68973500	1.45564400	-1.98518200
C	1.75133400	3.57837300	-1.76013300
H	1.81811000	3.75141600	-2.82661200
C	1.76821000	4.64838000	-0.87801800
C	1.66226200	4.40307700	0.48628400
H	1.64089200	5.22946700	1.18520900
C	1.56272200	3.10709400	0.95932800
H	1.46591100	2.94098300	2.02361600
C	2.65961500	0.50890100	1.66580900
H	2.80093900	1.36230600	2.31334600
C	3.53726900	-0.58707900	1.85664600
C	3.71292600	-1.49443600	0.77702900
C	2.80193900	-1.38178100	-0.30641800
C	4.72303000	-2.47357900	0.85477800
H	4.85425200	-3.17076500	0.03676100
C	5.51694600	-2.57011500	1.97256300
H	6.28156400	-3.33378900	2.02714400
C	5.33535600	-1.68449800	3.05062100
H	5.96462100	-1.77034800	3.92690700
C	4.36934900	-0.70965800	2.98951100
H	4.24008800	-0.01361900	3.80922100
H	1.84608500	5.66255000	-1.24706800
H	-1.84608500	5.66255000	-1.24706800

Racemization Experiments

(*P*)-**1a** (1 mg) was dissolved in 1,2-dichlorobenzene (1.0 mL), and the solution was heated at the indicated temperatures. The time-course of enantiomeric ratio was monitored by CSP-HPLC.

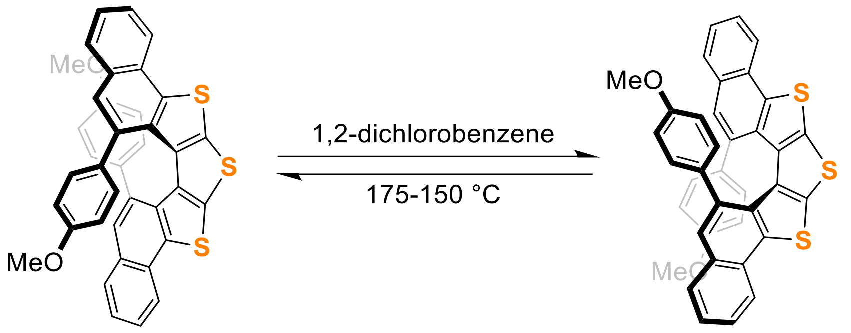

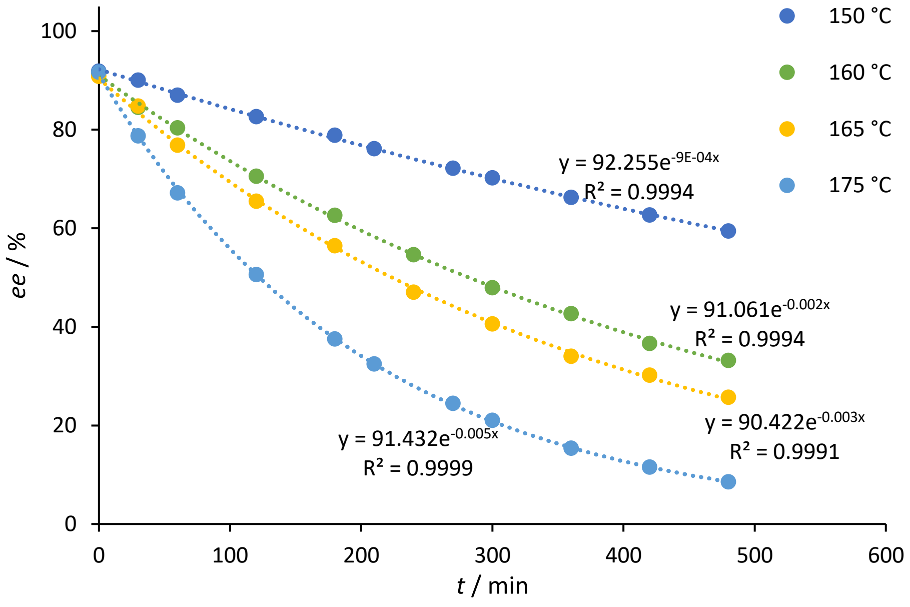

*Figure S 20: Time course of the ee of **1a** over time at different temperatures.*

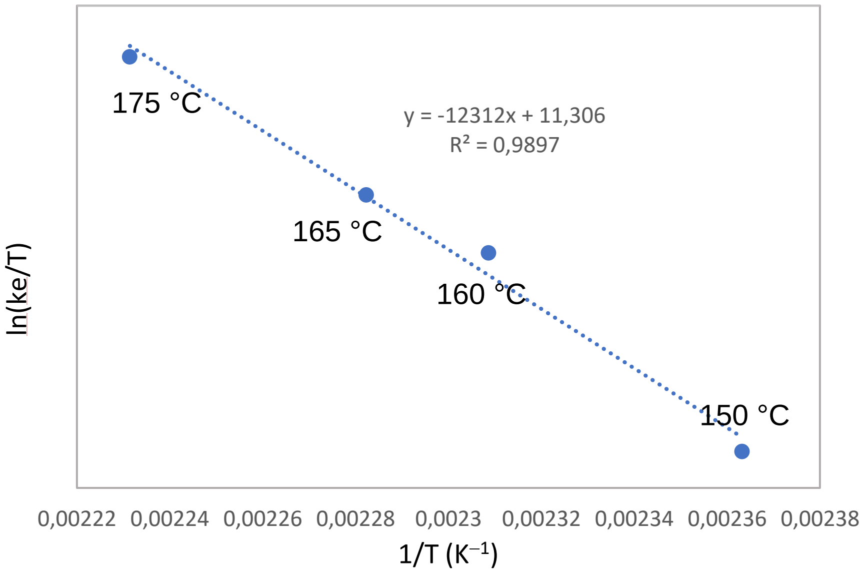

*Figure S 21: Eyring plot for **1a**.*

REFERENCES

[1] E. González-Fernández, L. D. M. Nicholls, L. D. Schaaf, C. Farès, C. W. Lehmann, M. Alcarazo, *J. Am. Chem. Soc.* **2017**, *139*,1428-1431.

[2] T. Hartung, R. Machleid, M. Simon, C. Golz, M. Alcarazo, *Angew. Chem. Int. Ed*. **2020**, *59*, 5660-5664.

[3] W. Fu, V. Pellicciolі, M. von Geyso, P. Redero, C. Böhmer, M. Simon, C. Golz, M. Alcarazo, *Adv. Mat.* **2023**, *35*, 2211279.

[4] H. Tinnermann, L. D. M. Nicholls, T. Johannsen, C. Wille, C. Golz, R. Goddard, M. Alcarazo, *ACS Catal.* **2018**, *8*, 10457–10463.

[5] Gaussian 16, Revision A.03, M. J. Frisch, G. W. Trucks, H. B. Schlegel, G. E. Scuseria, M. A. Robb, J. R. Cheeseman, G. Scalmani, V. Barone, G. A. Petersson, H. Nakatsuji, X. Li, M. Caricato, A. V. Marenich, J. Bloino, B. G. Janesko, R. Gomperts, B. Mennucci, H. P. Hratchian, J. V. Ortiz, A. F. Izmaylov, J. L. Sonnenberg, D. Williams-Young, F. Ding, F. Lipparini, F. Egidi, J. Goings, B. Peng, A. Petrone, T. Henderson, D. Ranasinghe, V. G. Zakrzewski, J. Gao, N. Rega, G. Zheng, W. Liang, M. Hada, M. Ehara, K. Toyota, R. Fukuda, J. Hasegawa, M. Ishida, T. Nakajima, Y. Honda, O. Kitao, H. Nakai, T. Vreven, K. Throssell, J. A. Montgomery, Jr., J. E. Peralta, F. Ogliaro, M. J. Bearpark, J. J. Heyd, E. N. Brothers, K. N. Kudin, V. N. Staroverov, T. A. Keith, R. Kobayashi, J. Normand, K. Raghavachari, A. P. Rendell, J. C. Burant, S. S. Iyengar, J. Tomasi, M. Cossi, J. M. Millam, M. Klene, C. Adamo, R. Cammi, J. W. Ochterski, R. L. Martin, K. Morokuma, O. Farkas, J. B. Foresman, and D. J. Fox, Gaussian, Inc., Wallingford CT, **2016**.
